# Cisplatin resistance in an ovarian cancer model is mediated by microtubule dynamics regulator TPPP3 in synergy with tubulin code rewiring

**DOI:** 10.1016/j.celrep.2026.117414

**Published:** 2026-06-04

**Authors:** Sachi Horibata, Kishore K. Mahalingan, Ruchi Patel, Yu Fan, Jordan M. Hotz, Adriana Ponton-Almodovar, Keith MacRenaris, Yan Li, Daoud Meerzaman, Michael M. Gottesman, Antonina Roll-Mecak

**Affiliations:** 1 Laboratory of Cell Biology, Center for Cancer Research, National Cancer Institute, National Institutes of Health, Bethesda, MD 20892, USA; 2 Precision Health Program, Michigan State University, East Lansing, MI 48824, USA; 3 Cell and Molecular Biology Program, Michigan State University, East Lansing, MI 48824, USA; 4 Department of Pharmacology and Toxicology, College of Human Medicine, Michigan State University, East Lansing, MI 48824, USA; 5 Cell Biology and Biophysics Unit, National Institute of Neurological Disorders and Stroke, National Institutes of Health, Bethesda, MD 20892, USA; 6 Computational Genomics and Bioinformatics Branch, Center for Biomedical Informatics & Information Technology, National Cancer Institute, National Institutes of Health, Rockville, MD 20850, USA; 7 Quantitative Bio Element Analysis and Mapping (QBEAM) Center, Michigan State University, East Lansing, MI 48824, USA; 8 Proteomics Core, National Institute of Neurological Disorders and Stroke, National Institutes of Health, Bethesda, MD 20892, USA; 9 Biochemistry and Biophysics Center, National Heart, Lung and Blood Institute, National Institutes of Health, Bethesda, MD 20892, USA; 10 These authors contributed equally; 11 Lead contact

## Abstract

Cisplatin is a mainstay in cancer treatment, but toxicity and resistance limit its potential. Using RNA sequencing (RNA-seq), we show that ovarian cancer cells undergo broad changes in the microtubule cytoskeleton that correlate with resistance. Consistent with this, we find that cisplatin directly impacts microtubule dynamics *in vitro* and in cells. Paclitaxel counteracts cisplatin and increases resistance. By purifying tubulin from cisplatin-sensitive, -resistant, and -re-sensitized ovarian cancer cells, we demonstrate that resistance acquisition rewires the tubulin code, leading to microtubule stabilization. Furthermore, tubulin polymerization-promoting protein 3 (TPPP3) contributes to resistance. In vitro, TPPP3 synergizes with tubulin isotypes from resistant cells to yield maximal microtubule stabilization in response to cisplatin. Database analysis shows that patients with low TPPP3 levels have improved therapeutic outcome. Our findings implicate TPPP3 and microtubule dysregulation in cisplatin resistance, independent of effects through DNA damage, and have bearing on the etiology of cisplatin-associated neuropathies and ototoxicity.

## In brief

Horibata et al. show that the cancer drug cisplatin destabilizes microtubules by accelerating loss of the GTP cap and identify tubulin polymerization-promoting protein 3 (TPPP3) as a microtubule dynamics regulator that confers cisplatin resistance and synergizes with tubulin code rewiring induced by cisplatin treatment.

## Graphical abstract

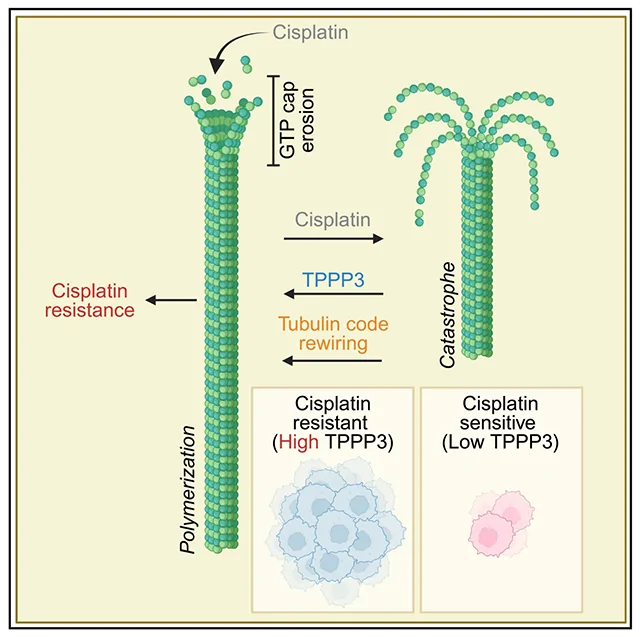

## INTRODUCTION

Cisplatin is a platinum-based chemotherapeutic agent. More than half a century after its discovery, cisplatin is one of the most used anti-cancer drugs in the world, employed to treat a broad spectrum of cancers including ovarian cancer.^1,2^ Cisplatin exerts its cytotoxic effect after cell entry and undergoes aquation reactions that substitute one or both *cis*-chloro groups with water molecules.^3,4^ This creates highly reactive aquated cisplatin that binds to nuclear DNA^5^ (and other macromolecules), and cytoplasmic nucleophiles such as reduced thiols and other cysteine-rich proteins.^6^ Cisplatin forms covalent bonds with DNA, breaking DNA strands, causing DNA damage, and leading to cell death.^7^ Binding of cisplatin to cytoplasmic nucleophiles results in oxidative stress, leading to DNA damage and cell death.^8^ However, toxicity and resistance limit cisplatin potential. Up to 80% of ovarian cancer patients develop tumor recurrence despite aggressive surgical removal and initial positive response to chemotherapy.^9–11^ To find more effective treatments, it is essential to understand how cisplatin resistance occurs.

Cisplatin resistance is multifactorial. The generally agreed-upon dominant resistance mechanisms include (1) reduced intracellular cisplatin accumulation, (2) enhanced DNA repair of platinum-DNA adducts, and (3) increased cisplatin detoxification in the cytoplasm via binding to glutathione and other thiols.^12^ Reduced intracellular accumulation can be due to reduced cisplatin uptake by plasma membrane transporters (e.g., CTR1)^13^ and increased cisplatin efflux via efflux pumps, as has been proposed (i.e., ATP7A/B).^14–16^ If cells are unable to eliminate cisplatin and the cisplatin binds to DNA, cisplatin-resistant cells activate DNA damage repair pathways to repair the DNA lesions, causing cisplatin resistance.^17^ The damaged DNA can be repaired by the nucleotide excision repair (NER) pathway, which incises both strands of the DNA lesions, followed by DNA synthesis to replace the damaged site.^18,19^ In addition, the DNA mismatch repair (MMR) pathway can also recognize and repair DNA specifically for erroneous insertions, deletions, and misincorporations of bases.^20^ These are some of the most studied cisplatin resistance mechanisms, with a primary focus on preventing and repairing DNA damage.^1,12^ However, only 1% of intracellular cisplatin binds to nuclear DNA, with the majority of the drug bound to proteins and RNA.^21^ Moreover, enucleated cells are also sensitive to cisplatin,^22^ warranting further investigation of non-DNA-mediated anti-cancer effects of cisplatin and mechanisms of resistance.

Cisplatin has been shown to covalently bind to glutathione, one of the most abundant thiols in cells, and this cisplatin-glutathione complex can be exported out of cells via multidrug resistance protein (MRP1 or MRP2).^1,12,23^ Metallothioneins, cysteine-rich proteins that can detoxify heavy metals, inactivate cisplatin, which limits the amount of cisplatin available to bind to DNA and thus DNA damage.^1,24–27^ More recently, cisplatin has been shown to bind to RNA and cause apoptosis and enhancement of pro-inflammatory signatures.^28^ However, whether RNA damage is involved in cisplatin resistance is unknown, and little is still known about non-DNA-mediated anti-cancer effects of cisplatin and mechanisms of resistance. Cisplatin resistance is also accompanied by changes in the expression of microtubule cytoskeleton genes^29,30^; however, the mechanistic basis for these changes is not known.

By performing microscopy-based *in vitro* microtubule dynamics assays, we show that clinical concentrations of cisplatin destabilize microtubule ends directly, promoting catastrophe. By purifying tubulin from ovarian cancer cells sensitive, resistant, and re-sensitized to cisplatin, we find that cisplatin-resistant cells change their tubulin isotype repertoire (tubulin isoforms and posttranslational modifications), i.e., their “tubulin code,”^31,32^ and that these changes stabilize microtubules, directly counteracting the effects of cisplatin in resistant cells but not in cisplatin-sensitive or -re-sensitized cells. Furthermore, we find that paclitaxel, a microtubule-stabilizing agent, counteracts the effects of cisplatin on microtubules and increases cisplatin resistance in an *in vitro* ovarian cancer model. Using RNA sequencing (RNA-seq) analysis coupled with functional analyses, we show that a novel microtubule dynamics regulator, tubulin polymerization-promoting protein 3 (TPPP3), one of the top ten genes overexpressed in cisplatin-resistant cells, causes cisplatin resistance in two different ovarian cancer cell lines. High levels of TPPP3 strongly correlate with cisplatin resistance and unfavorable clinical outcomes in cancer patients. We demonstrate that the tubulin repertoire in the cisplatin-resistant cells, but not in the sensitive or re-sensitized cells, synergizes with TPPP3 for maximal stabilization of the microtubule cytoskeleton in response to cisplatin, counter-acting its destabilizing effects. Our studies reveal a cisplatin-induced cellular rewiring of the tubulin code both at the level of tubulin substrate and effector and shed light on mechanisms of cisplatin cytotoxicity not induced by DNA damage. In addition, our findings hold potential clinical implications especially in the setting of ovarian cancer as ovarian cancer patients are administered a platinum agent in combination with paclitaxel.

## RESULTS

### Broad cytoskeletal changes correlate with cisplatin resistance

**Figure 1.**
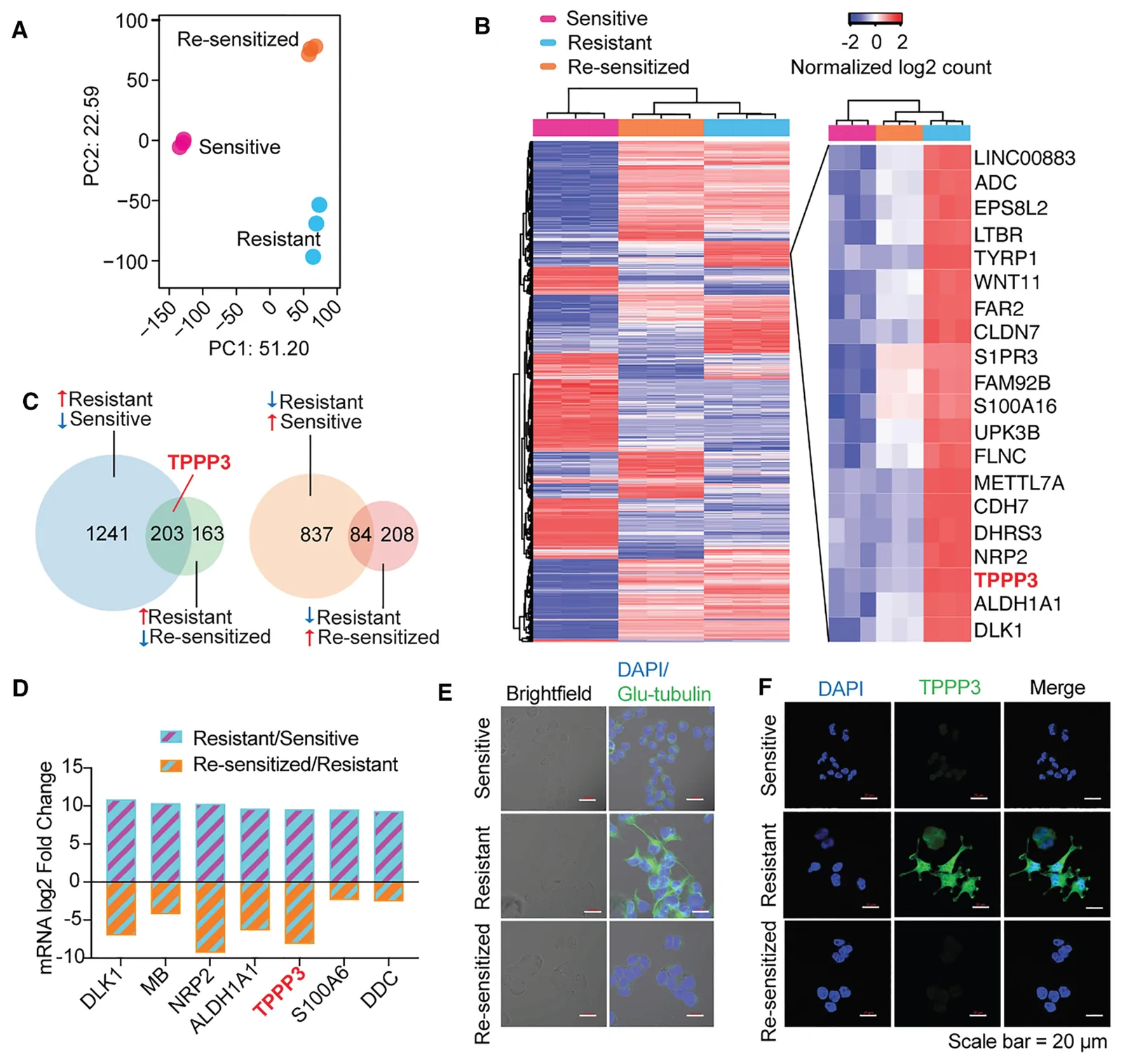
Transcriptional signature in cisplatin-resistant cells shows broad changes in the microtubule cytoskeleton (A) Principal-component analysis based on the RNA-seq data from cisplatin-sensitive, -resistant, and -re-sensitized ovarian cancer cells reveal distinct transcriptional signatures among the three cell lines. (B) Heatmap representing differentially expressed genes in the three cell lines (FDR < 0.01, absolute log FC > 2) identifies TPPP3 as one of the top genes up-regulated in resistant cells compared to sensitive or re-sensitized cells. (C) Venn diagrams depicting the number of genes differentially expressed. (D) Log2 fold-change difference between the mRNA levels of the resistant versus sensitive group and re-sensitized versus resistant group. (E) Bright-field and immunofluorescence images of sensitive, resistant, and re-sensitized ovarian cancer cells stained with DAPI and Glu-tubulin. Scale bars, 20 μm. (F) Immunofluorescence images of sensitive, resistant, and re-sensitized ovarian cancer cells stained with DAPI and TPPP3 show cytoplasmic localization of TPPP3. Scale bars, 20 μm.

We performed transcriptomic studies on *in vitro*-derived cisplatin-sensitive (1A9), -resistant (1A9CP80), and -re-sensitized (1A9CP80R) ovarian cancer cells (Figures 1A–1D, and S1A; see STAR Methods). These cells were derived from a patient with no prior exposure to anti-cancer agents, making them a powerful model system to study drug response and identify genes involved in resistance.^33^ The cisplatin sensitivity of these cells was verified (IC50 ~3 μM; Figures S1B–S1D), and unbiased hierarchical clustering categorized sensitive, resistant, and re-sensitized cells in distantly related groups (Figure 1A). The re-sensitized cells are originally resistant cells (IC50 ~88 μM) that regained partial sensitivity (IC50 ~;35 μM) after cisplatin withdrawal. For simplicity, these partially re-sensitized cells will be named re-sensitized cells hereafter. However, even though the re-sensitized cells regained partial cisplatin sensitivity, their transcriptional profile did not revert to the one close to the sensitive cells, indicating a new transcriptional state after cisplatin removal. Inductively coupled plasma mass spectrometric (ICP-MS) analyses show similar cisplatin accumulation among the three cell lines (Figure S1E; see STAR Methods), indicating that resistance is not due to reduced cisplatin uptake. Furthermore, unlike the sensitive cells, the resistant and resensitized cells do not show either p53 induction, regardless of cisplatin concentration (Figure S2A), or a marked increase in the phosphorylation of the H2AX histone variant upon cisplatin exposure (Figure S2B), both indicators of DNA damage. Thus, two major causes of resistance to cisplatin, reduced uptake of cisplatin and repair of DNA damage, are not solely responsible for the observed resistance, and re-sensitization to cisplatin is driven by additional mechanisms not related to DNA damage or uptake.

RNA-seq identified 2,365 genes differentially transcribed in resistant cells compared to sensitive cells (false discovery rate [FDR] < 0.01, absolute log fold change [FC] > 2), 1,444 of which were transcribed more highly in resistant cells and 921 that were transcribed more highly in sensitive cells (Figures 1B, 1C, and S3A; Data S1). Among the top 20 upregulated genes in resistant cells compared to sensitive cells were genes previously implicated in cisplatin resistance across multiple cancers, including neuropilin-2 (*NRP2*), *S100A10*, and *IGFBP2* (Figure S3A; Data S1). When we compared gene expression signatures of re-sensitized and resistant cells to identify genes that may be involved in regaining cisplatin sensitivity, we identified 658 genes (FDR < 0.01, absolute log FC > 2), 292 of which were transcribed more highly in re-sensitized cells and 366 that were transcribed more highly in resistant cells (Figures 1B, 1C, and S3B; Data S2). *DLK1*, *MB*, *NRP2*, *ALDH1A1*, *TPPP3*, *S100A6*, and *DDC* were the top genes upregulated in resistant cells compared to the sensitive or re-sensitized cells (Figures 1D, S3A, and S3B). Among these, only NRP2 and TPPP3 reverted to expression levels in the re-sensitized cells similar to those in the sensitive cells (Figure 1D; Data S2). NRP2, the VEGF receptor, has been previously reported to promote DNA repair as a mechanism for cisplatin resistance.^34^ TPPP3 has no known involvement in cisplatin resistance. Moreover, our transcriptomics analysis revealed that cisplatin resistance is accompanied by broad changes in the expression of microtubule cytoskeleton genes (Data S3 and S4), consistent with differences in cellular morphology in the resistant cells (Figure S1A), which have protrusions enriched in detyrosinated microtubules (Figure 1E). Microtubule-rich protrusions have been reported in ovarian cancer cells and are thought to be linked to increased metastatic potential.^35^ TPPP3 showed the highest changes in expression between the resistant and sensitive cells, and between the resistant and re-sensitized cells, out of the 203 genes whose expression tracked with cisplatin resistance (Figures 1B–1D; Data S1 and S2). TPPP3 is upregulated at both the mRNA and protein levels, with the resistant cells expressing ~30-fold higher levels of TPPP3 protein compared to the sensitive cells (Figures S3C–S3E). Immunofluorescence shows the cytoplasmic distribution of TPPP3 in the resistant cells (Figure 1F). Analysis of data from a recent survey revealed that TPPP3 expression is increased in many cisplatin-resistant ovarian cell lines.^36^

### Cisplatin at therapeutic concentrations promotes microtubule catastrophe

**Figure 2.**
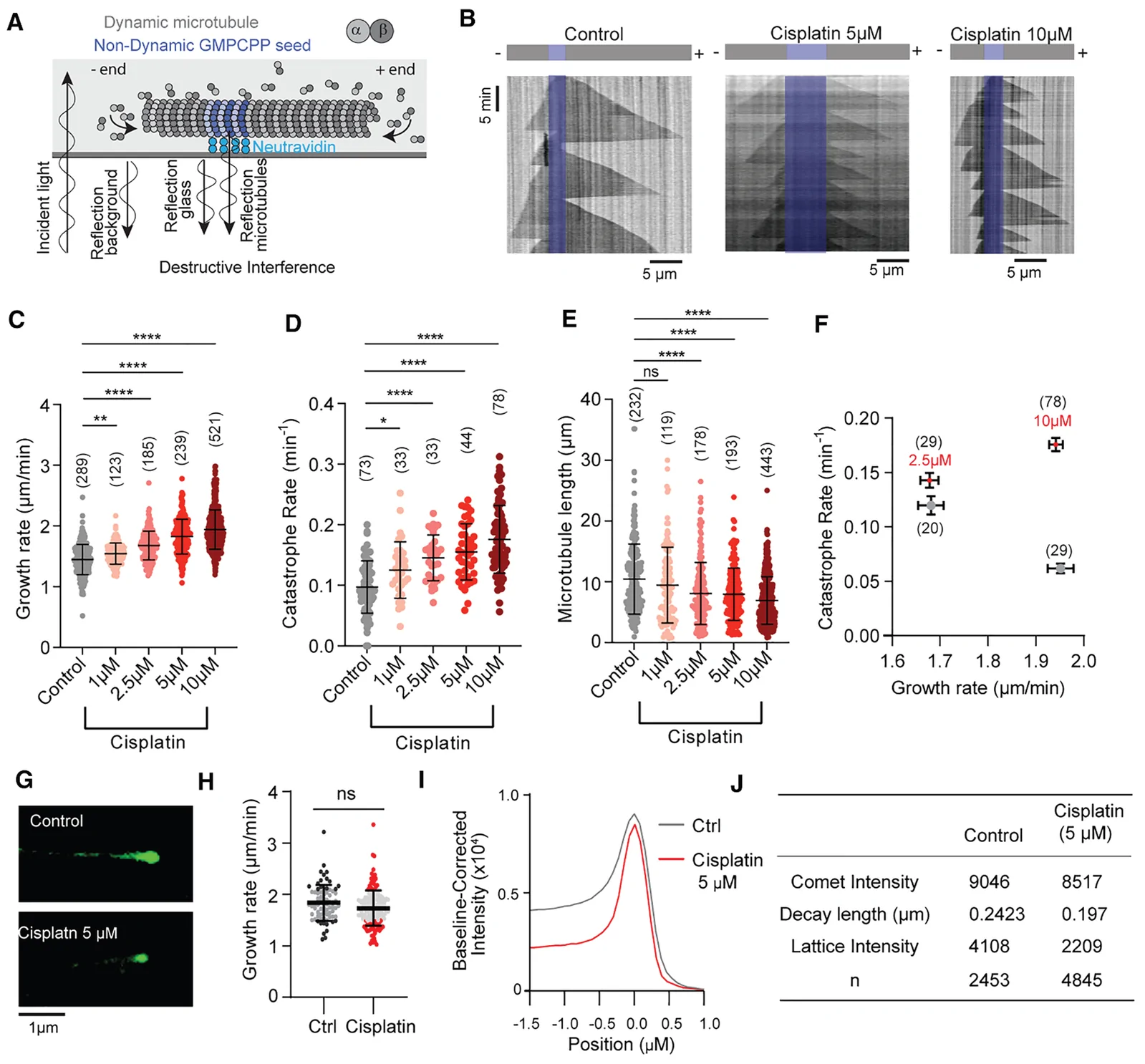
Cisplatin at therapeutic concentrations induces catastrophes by eroding the GTP cap (A) Schematic of interference reflection microscopy (IRM)-based microtubule dynamics assay. (B) Kymographs showing microtubule groth at 10 μM brain tubulin in the presence of 5 and 10 μM cisplatin; GMPCPP-stabilized seeds highlighted in blue. Scale bars, 5 μm (horizontal) and 5 min (vertical). (C–E) Microtubule plus-end growth rates (C), microtubule plus-end catastrophe rates (D), and microtubule lengths (E) at 10 μM porcine brain tubulin with different cisplatin concentrations. Error bars, SD. (F) Catastrophe rates as a function of microtubule growth rates in the absence (gray) or presence (red) of cisplatin; cisplatin concentration indicated above data points. Error bars, SEM; for (C) through (F) *n* values indicated in parentheses throughout from 8, 4, 5, 7, and 6 independent experiments on different days for 0, 1, 2.5, 5, and 10 μM cisplatin, respectively; ****p < 0.0001, **p < 0.01, *p < 0.05, and ns, non-significant, as determined by one-way ANOVA using Tukey’s multiple comparison test. (G) TIRF microscopy images of EB1-GFP (300 nM, green) on brain microtubules in the absence or presence of 5 μM cisplatin. Scale bars, 1 μm. (H) Plus-end growth rates of microtubules with and without cisplatin in the presence of EB1-GFP. The growth rates of microtubules within 20% of the control mean are highlighted in gray and were used in the comet profile analysis. (I) Averaged fluorescent intensity profiles of EB1-GFP comets on microtubules with and without cisplatin. (J) Comet profile parameters. Comet profiles were fitted using a Gaussian (−1.5 to 0 μm) and a single exponential function (0–1.5 μm) (STAR Methods).

The changes in microtubule cytoskeleton genes revealed by our analyses prompted us to investigate the direct effect of cisplatin itself on microtubule dynamics. Microtubules assemble through the addition of tubulin subunits at their ends. They exhibit a behavior known as “dynamic instability,” i.e., they grow and shrink stochastically. This behavior is crucial for microtubule-based processes such as chromosome capture and polarity establishment.^37,38^ Pharmacokinetic studies show that in cancer patients undergoing cisplatin-based chemotherapy, the maximum blood plasma cisplatin concentration achieved is ~33 μM and persists at ~5–10 μM over 24 h.^39,40^ Cell culture experiments show free intracellular cisplatin concentrations in the low μM range.^41^ At these cisplatin concentrations, microscopy-based single-microtubule *in vitro* dynamics assays with tubulin isolated from brain tissue (Figure 2A; see STAR Methods) revealed that cisplatin increases microtubule growth rates in a dose-dependent manner, resulting in increases of 7%, 16%, 27%, and 35% at 1, 2.5, 5, and 10 μM cisplatin, respectively (Figures 2B and 2C; Video S1). These effects were visible within a minute of exposure to cisplatin. Strikingly, this increase in growth rate is accompanied by an increase in the frequency at which microtubules switch from a state of growth to one of depolymerization, known as catastrophe (Figures 2B and 2D). Catastrophe rates increase 28%, 49%, 58% and 79% at 1, 2.5, 5 and 10 μM cisplatin, respectively. The magnitude of the effects observed are comparable to those reported for vinblastine and paclitaxel at therapeutic concentrations^42,43^ or for microtubule regulators^44–46^ or tubulin disease mutants.^47,48^ As a result of this destabilizing effect of cisplatin, the mean length of microtubules decreases steadily with cisplatin concentration (Figure 2E). The effect of cisplatin on microtubule dynamics can also be expressed using a parameter called “dynamicity,” which sums the absolute values of the magnitude of growth and shrinkage per unit time to reveal an increased dynamicity of 3.6 and 3.7 μm/ min at 5 and 10 μM cisplatin, respectively, compared to 1.8 μm/min in the control. Thus, when exposed to cisplatin, microtubules grow faster but have shorter periods of sustained growth. Interestingly, carboplatin, another platinum-based chemotherapy drug, also increases microtubule catastrophe rates when used at therapeutic concentrations,^49,50^ but unlike cisplatin, it does this while decreasing microtubule growth rates (Figure S4). The mechanistic basis for the difference in response between these two different platinum-based drugs will be an interesting area of future study.

Microtubules grow by the addition of GTP-tubulin subunits at their ends. Once GTP-tubulin is incorporated into the microtubule, the GTP is hydrolyzed to GDP. Growing microtubules are protected against spontaneous depolymerization by a layer of GTP-tubulin, a “GTP cap” at their ends.^51–54^ The cap is formed because the rate of addition of GTP-tubulin subunits at the microtubule end is faster than the GTP hydrolysis rate. The microtubule depolymerizes when the GTP cap is lost. Thus, the faster the microtubule grows, the larger the GTP cap and catastrophe rates decrease. However, in the presence of cisplatin, microtubule catastrophe rates increase steadily even as growth rates increase (Figures 2C and 2D). When comparing microtubules with similar growth rates by growing them at different tubulin concentrations (see STAR Methods), catastrophe rates are ~2.8-fold higher in the presence of 10 μM cisplatin than in the growth-matched control without cisplatin (Figures 2F and S5A–S5C). At 20 μM cisplatin, catastrophe rates are 0.82 min^−1^, almost 7-fold higher than in the growth-matched control without cisplatin (Figures 2F and S5D–S5F). These results are consistent with a faster erosion of the GTP cap in the presence of cisplatin. Consistent with this, we also see that cisplatin affects the profile of end-binding protein 1 (EB1) at microtubule ends. EB1 preferentially binds to GTP or GDP-Pi tubulin.^54,55^ EB1 detaches when the tubulin at the microtubule end switches from a GTP-bound to a GDP-bound state during polymerization, creating the characteristic comet-like signal at the growing tip (Figure 2G). The comet decay rate is a readout of the rate of GTP hydrolysis. We therefore analyzed EB1 comet profiles (STAR Methods) in the absence or presence of cisplatin, at matched microtubule growth speeds (Figure 2H). This analysis shows a significantly steeper decay of the comet profile in the presence of cisplatin, indicative of faster GTP hydrolysis rate (Figures 2I and 2J). *In toto*, our experiments show that cisplatin destabilizes microtubule ends and accelerates catastrophe by eroding the GTP cap.

### Paclitaxel counteracts cisplatin and increases resistance

**Figure 3.**
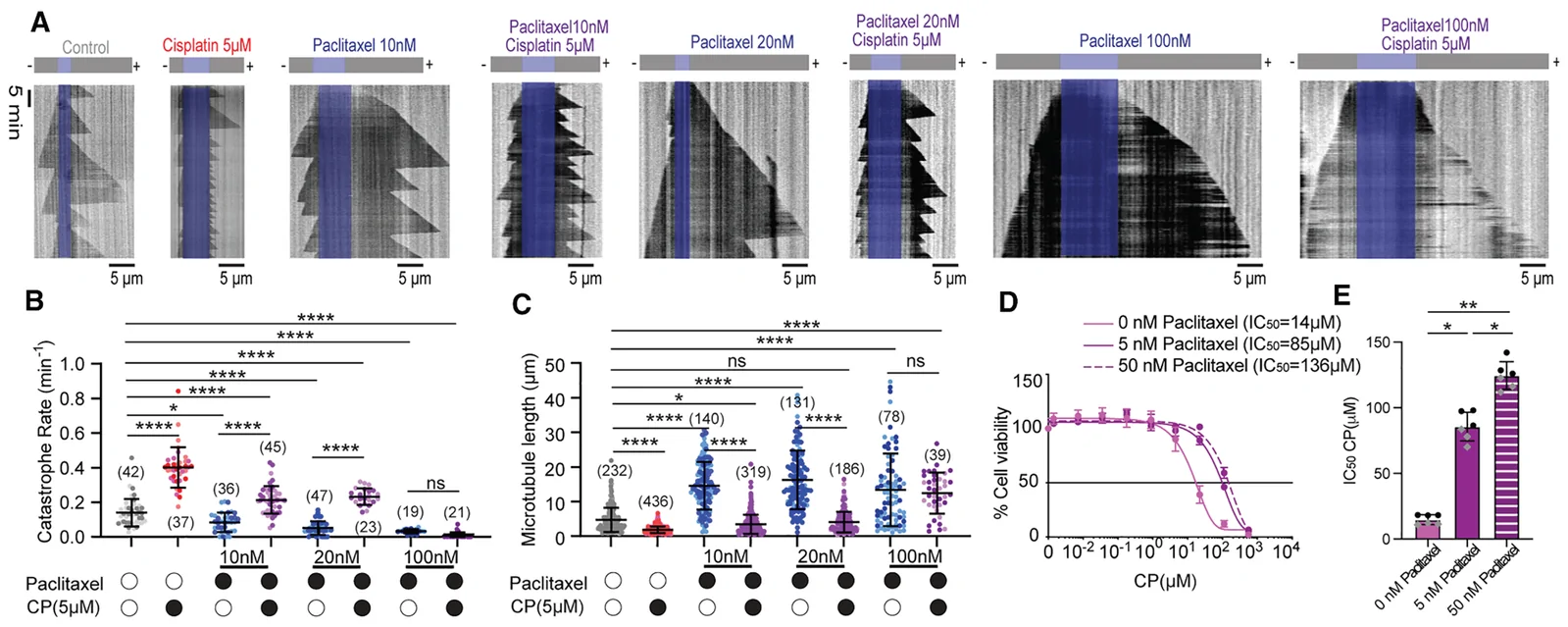
Cisplatin and paclitaxel have antagonistic effects **(A)** Kymographs showing microtubule growth at 6 μM porcine brain tubulin in the presence of paclitaxel alone, cisplatin alone, and in combination with paclitaxel at different concentrations; GMPCPP-stabilized seed highlighted in blue. Scale bars, 5 μm (horizontal) and 5 min (vertical). (B and C) Microtubule plus-end catastrophe rates (B) and lengths (C) at 6 μM of porcine brain tubulin in the presence of paclitaxel alone, cisplatin alone, and in combination with paclitaxel at different concentrations. Error bars, SD; *n* values shown in parentheses throughout, growth events for (B) and microtubules for (C) from 2, 3, 2, 2, 2, 2, and 2 independent experiments on different days for control, 5 μM cisplatin, 10 nM paclitaxel, 10 nM paclitaxel and 5 μM cisplatin, 20 nM paclitaxel, 20 nM paclitaxel and 5 μM cisplatin, 100 nM paclitaxel, and 100 nM paclitaxel and 5 μM cisplatin, respectively. ****p < 0.0001, **p < 0.01, *p < 0.1, and ns, non-significant, as determined by one-way ANOVA using Tukey’s multiple comparison test. (D) Cytotoxicity assay of sensitive cells treated with increasing concentration of cisplatin in the presence of 0, 5, or 50 nM paclitaxel, *n* = 2 independent experiments on different days. (E) IC50 values for the data shown in (D). *p < 0.05 and **p < 0.005, as determined by one-way ANOVA using Tukey’s multiple comparison test.

Since our results show that cisplatin destabilizes microtubules, we tested the effect of the microtubule-stabilizing agent paclitaxel when combined with cisplatin because the standard ovarian cancer treatment is platinum-based chemotherapy in combination with paclitaxel, a regime that has remained unchanged for the past three decades.^56^ We found that paclitaxel counteracts the effects of cisplatin by decreasing catastrophe rates (Figures 3A, 3B, S6A, and S6B). As a result, the average length of microtubules treated with 5 μM cisplatin is 1.8 μm, while those treated with a combination of cisplatin and 10 nM paclitaxel are 3.5 μm and those treated with 20 nM paclitaxel are 4.1 μm (Figure 3C). At 100 nM, paclitaxel completely overcomes the destabilizing effects of cisplatin. We then tested the effects of 5 and 50 nM paclitaxel on the response to cisplatin of the sensitive ovarian cancer cells. Previous studies have shown that treatment of cultured cells at these paclitaxel concentrations results in clinically relevant intracellular drug concentrations.^57^ Motivated by our *in vitro* microtubule dynamics results, we examined cell viability as a function of increasing cisplatin concentration in the presence of paclitaxel (Figure 3D). We found that paclitaxel does indeed increase resistance to cisplatin (Figure 3E), suggesting an involvement of increased microtubule stability in cisplatin resistance. Importantly, we note that the presence of paclitaxel alone at these concentrations causes cellular death (Figure S6C).

### Tubulin isotypes and intrinsic microtubule dynamics change with cisplatin resistance

Given the destabilizing effects of cisplatin on microtubules, we hypothesized that cisplatin-resistant cells evolved mechanisms to counteract this effect. Traditionally, the effects of microtubule regulators or drugs have been investigated on tubulin purified from brain tissue because of its abundance and ease of purification.^31^ However, the isotype composition (isoforms and posttranslational modifications) of brain tubulin bears little resemblance to that of most other cell types,^58^ and studies have shown that tubulin isoforms regulate microtubule dynamics directly.^59–63^ This tubulin diversity is part of a tubulin code that fine-tunes the microtubule cytoskeleton for specialized functions.^31,32^ Moreover, our transcriptomics analysis revealed that cisplatin resistance is accompanied by broad changes in the expression of several tubulin isoforms (e.g., *TUBB4A*, *TUBB3*, *TUBA1A*, and *TUBB2B*) as well as tubulin posttranslational modification enzymes (*TTLL7* and *AGTPBP1*)*. TUBB4A*, *TUBB3*, *TUBA1A*, *and TTLL7* are upregulated in resistant cells compared to sensitive and re-sensitized cells (FDR < 0.01, absolute log FC > 2), while *TUBB2B and AGTPBP1* are downregulated (FDR < 0.01, absolute log FC > 2) (Data S1). A previous proteomics and data mining study also revealed that cytoskeleton proteins accounted for more than 22% of the genes differentially expressed in cisplatin-resistant neuroblastoma cells.^64^

Tubulin isoform changes are well documented in several drug-resistant cancers^30,65,66^; however, because the tubulins have not been directly examined, it has remained unknown whether these changes impact microtubule properties, and if they do, whether the effect is either direct or in *trans* by affecting the interaction of microtubules with molecular effectors. Using methodological advances in the isolation of tubulin from non-brain tissue,^67^ we purified tubulin from the sensitive, resistant, and re-sensitized cells using an affinity purification strategy with a tumor overexpressed gene (TOG) domain-functionalized resin^68^ (STAR Methods) to analyze both its intrinsic polymerization properties and how it interacts with TPPP3. Mass spectrometric analysis confirmed the purity of the samples and that indeed tubulin isoforms in these cells are different (Figures S7A–S7C). Consistent with our RNA-seq analysis, tandem mass spectroscopy revealed that the levels of tubulin isotypes and tubulin posttranslational modifications were different in the three cell lines (Figures S7 and S8; Data S1). Notably, several tubulin isoforms, both α- and β-tubulin, and tubulin modifications tracked with cisplatin resistance (Figures S8A–S8D; Table S1). Tubulin isoforms βIII and βIVA were significantly enriched in the resistant cells compared to the sensitive and re-sensitized cells (Figures S8A–S8D), consistent with our RNA-seq analysis (Data S3 and S4). Our results align well with previous reports of changes in β-tubulin isoforms in response to platinum-based chemotherapy,^29,69–71^ in particular the correlation of the βIII isoform with cisplatin resistance.^69^

**Figure 4.**
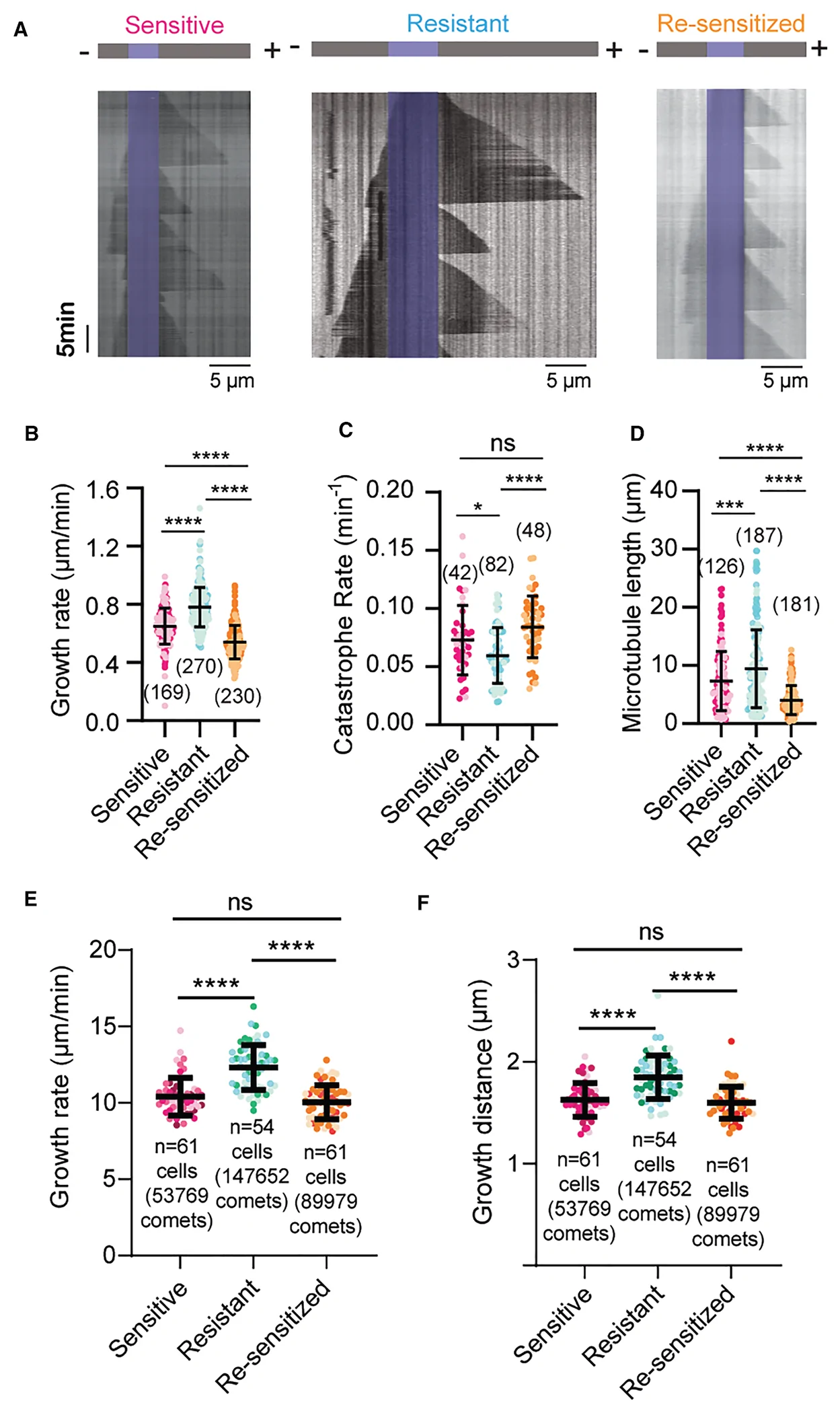
Dynamics of microtubules assembled from tubulin purified from cisplatin-sensitive, -resistant, and -re-sensitized cells show that microtubule stability tracks with resistance (A) Kymographs showing microtubule growth at 6 μM tubulin purified from cisplatin-sensitive, -resistant, and -re-sensitized cells; GMPCPP-stabilized seeds highlighted in blue. Scale bars, 5 μm (horizontal) and 5 min (vertical). (B–D) Microtubule plus-end growth rates (B), microtubule plus-end catastrophe rates (C), and microtubule lengths (D) at 6 μM of tubulin purified from sensitive (magenta), resistant (cyan), and re-sensitized (orange) cells. Error bars, SD. *n* values indicated in parentheses throughout from 2 independent experiments on different days for each condition. Tubulin purified from an independent second preparation shows similar dynamic parameters (Figures S6D–S6F). Data points collected from independent experiments shown in different shades. ****p < 0.0001, ***p = 0.01, *p < 0.05, and ns, non-significant, as determined by one-way ANOVA using Tukey’s multiple comparison test. (E and F) Scatterplots of microtubule growth rate (E) and microtubule growth distance (F) obtained by tracking EB3-mScarlet in cisplatin-sensitive, -resistant, and -re-sensitized cells. Number of EB3 comets and cells analyzed indicated on the graphs. *n* 3 independent experiments on different days. Data points collected on different days and from independent experiments are shown in different shades. Error bars, SD. ****p < 0.0001 and ns, non-significant, as determined by one-way ANOVA using Tukey’s multiple comparison test.

To establish the functional effects of these broad changes in tubulin repertoire, we reconstituted microtubules dynamics *in vitro* by nucleating microtubules from GMPCPP-stabilized seeds and imaged the microtubules by label-free interference reflection microscopy (Figure 4A). These showed that the tubulin purified from the three lines has markedly different dynamic parameters (Figures 4A–4D). These differences were consistent between independent cell growths and tubulin preparations (Figures S7D–S7F). Microtubules assembled from tubulin purified from the resistant cells grow faster and depolymerize less frequently (Figures 4A–4C; Video S2), resulting in an overall increase in mean microtubule length compared to microtubules assembled from tubulin purified from the sensitive or re-sensitized cells, 9.4 μm versus 7.3 and 4 μm, respectively (Figure 4D). Interestingly, in addition to the changes in the isoform repertoire, several tubulin posttranslational modifications that we track with cisplatin resistance have been associated with microtubule stabilization. Notably, phosphorylation at multiple Ser residues on the tubulin C-terminal tail, which increases in the cisplatin-resistant cells and decreases in the re-sensitized cells (Figure S8C), was previously linked to increased microtubule assembly and stability in cells.^31,72–74^ We also observed a pronounced increase in βIII and βIIA/IIB tubulin glutamylation in the resistant cells compared to the sensitive cells (Table S1). Increased tubulin glutamylation in the resistant cells is also consistent with our transcriptional analysis, which revealed upregulation of TTLL glutamylase expression and down-regulation of CCP deglutamylases. Glutamylation does not stabilize microtubules directly^75^ but regulates the association of microtubule-associated proteins,^76–78^ can contribute to increased microtubule stability in cells,^79^ and promotes metastasis.^79^ Thus, acquisition of cisplatin resistance elicits broad changes in the microtubule isotype repertoire that directly increases microtubule stability as demonstrated by our *in vitro* reconstitution experiments (Figure 4A), counteracting the destabilizing effects of cisplatin.

We then extended our *in vitro* microtubule dynamics studies in cisplatin-sensitive, -resistant, and -re-sensitized cells by tracking end-binding protein 3 (EB3) comets. As in our *in vitro* experiments, we observed significantly higher microtubule growth rates and comet growth distances in cisplatin-resistant cells compared to sensitive or re-sensitized cells (Figures 4E and 4F). Therefore, our *in vitro* and cellular studies show that cisplatin resistance is accompanied by an increase in tubulin polymerization, and our *in vitro* studies with purified tubulin demonstrate that this effect is, at least partially, directly due to the different intrinsic properties of the different tubulin isoforms found in the sensitive, resistant, and re-sensitized cells.

### TPPP3 synergizes with tubulin isotypes to counteract cisplatin in resistant cells

We next focused on TPPP3 since its protein levels are upregulated ~30-fold in the resistant cells (Figures S3D and S3E). TPPP3 belongs to the TPPP family of microtubule-associated proteins.^80,81^ Despite its name, the physiological functions of TPPP3 are unknown. We found that TPPP3 binds brain microtubules with an apparent K_D_ of ~2.3 μM (Figure S9A). However, unlike its close homolog TPPP1,^80^ and despite its name, TPPP3 does not nucleate microtubules even at high concentrations (Figures S9B and S9C). We then used CRISPR-Cas9 to knock out TPPP3 in the resistant cells (Figures S9D–S9F) and examined the effects of TPPP3 loss on microtubule dynamics by tracking EB3. These showed that loss of TPPP3 in both knockout cell lines (TPPP3-KO1 and KO2) impairs microtubule growth (Figures S9G and S9H). Therefore, both our *in vitro* and cellular data show that TPPP3 is a microtubule regulator.

**Figure 5.**
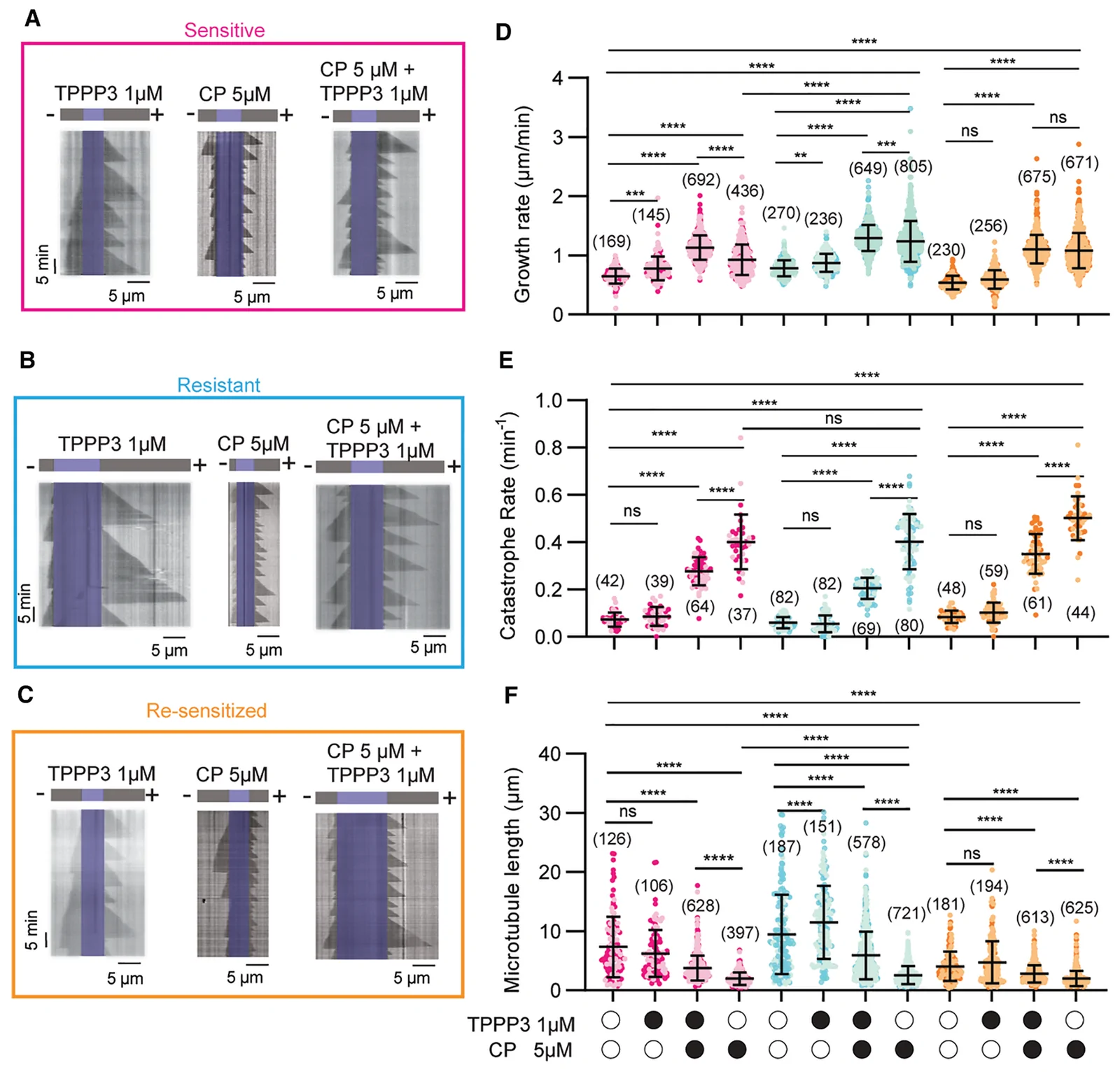
TPPP3 synergizes with tubulin isotypes from cisplatin-resistant cells to stabilize microtubules and counteract the toxic effects of cisplatin *in vitro* (A–C) Kymographs at 6 μM tubulin purified from cisplatin (CP)-sensitive (A), -resistant (B), and -re-sensitized (C) cells in the presence of 1 μM TPPP3, 5 μM CP, and 1 μM TPPP3 with 5 μM CP. GMPCPP-stabilized seeds highlighted in blue. Scale bars, 5 μm (horizontal) and 5 min (vertical). (D–F) Microtubule plus-end growth rates (D), microtubule plus-end catastrophe rates (E), and microtubule lengths (F) at 6 μM tubulin purified from CP-sensitive (magenta), -resistant (cyan), and -re-sensitized (orange) cells in the presence of 1 μM TPPP3, μM CP, and 1 μM TPPP3 with 5 μM CP. Error bars, SD; *n* values indicated in parentheses throughout from 2 independent experiments on different days for each condition. Data points collected from independent experiments shown in different shades. ****p < 0.0001, ***p < 0.001, **p < 0.01, and ns, non-significant, as determined by one-way ANOVA using Tukey’s multiple comparison test.

We next investigated the effects of TPPP3 on the dynamics of tubulin purified from the sensitive, resistant, and re-sensitized cell lines. We found that the addition of TPPP3 at 1 μM, the cellular concentration of TPPP3 in the cisplatin-resistant cells (Figure S9D), increases growth rates of sensitive and resistant microtubules by 20% and 13%, respectively (Figures 5A–5D), while having no appreciable effect on catastrophe rates (Figure 5E) for all microtubule types. Cisplatin increases catastrophe rates for all microtubule types, but the addition of TPPP3 significantly reduces catastrophe rates in the presence of cisplatin only. Notably, TPPP3 has the most pronounced stabilizing effect with the tubulin isolated from the resistant cells (Figures 5E and 5F). Addition of 1 μM TPPP3 to tubulin purified from resistant cells reduced the catastrophe rate by 2-fold, from 0.4 to 0.2 min^−1^ (Figure 5E). In contrast, catastrophe rates are reduced by only 30% and 29% with tubulin purified from the sensitive and re-sensitized cells, respectively. Thus, TPPP3 antagonizes maximally the destabilizing effects of cisplatin with the tubulin isotype repertoire found in resistant cells and not the sensitive or re-sensitized ones. This differential effect results in an average microtubule length of 6 μm even in the presence of 5 μM cisplatin, compared to ~3.7 and ~2.8 μm for the sensitive and re-sensitized tubulin, respectively, and is close to the average length of microtubules from sensitive cells in the absence of cisplatin, ~6 μm (Figure 5F; Video S3). Together, our *in vitro* microtubule dynamics reconstitution reveals that TPPP3 synergizes with the tubulin isotype repertoire in resistant cells to maximally stabilize microtubules against the toxic destabilizing effects of cisplatin. Consistent with this synergy, total internal reflection (TIRF) microscopy-based microtubule binding assays with purified TPPP3-GFP (STAR Methods) show increased binding of TPPP3-GFP to microtubules assembled from the tubulin isoform repertoire found in resistant cells, compared to that found in the sensitive or re-sensitized cells (Figures S10A–S10C).

### TPPP3 depletion increases cisplatin sensitivity in resistant cells

**Figure 6.**
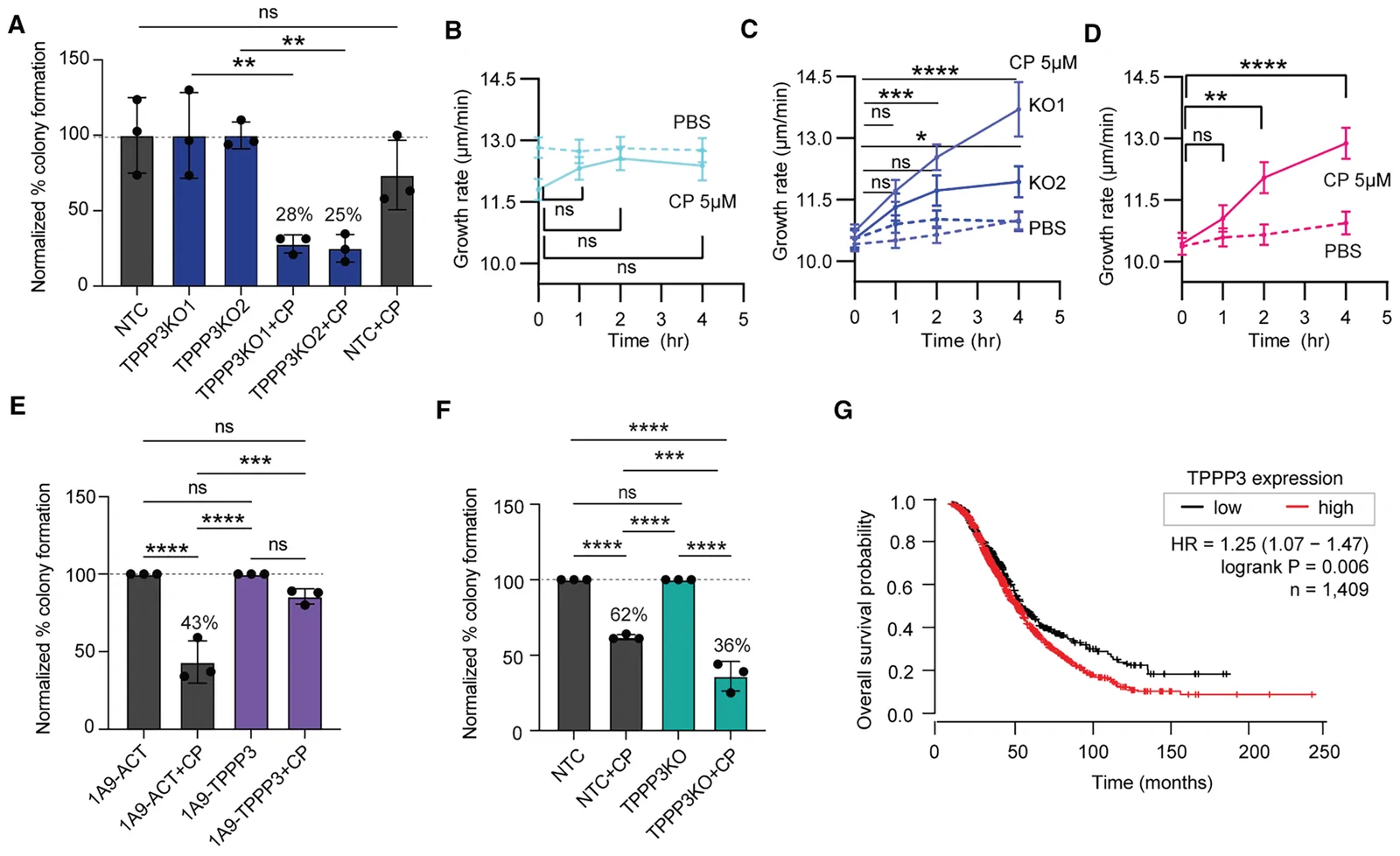
TPPP3 contributes to cisplatin resistance in cellular models and is a predictive biomarker of cisplatin resistance (A) Soft-agar cytotoxicity assay of non-targeting CRISPR-resistant cells (NTCs) and TPPP3-KO cells treated with 25.6 μM cisplatin shows re-sensitization of cisplatin-resistant 1A9CP80 cells to cisplatin when TPPP3 is knocked out; *n* = 3 independent experiments for each KO line, **p < 0.005, and ns, non-significant, as determined by one-way ANOVA using Tukey’s multiple comparison test. (B–D) Microtubule growth rates in response to cisplatin addition for cisplatin-resistant cells (B), TPPP3-KO cells (C), and cisplatin-sensitive cells (D). PBS buffer control shown as a discontinuous line. For cisplatin treatment, *n* = 31, 30, 29, and 24 resistant cells at 0, 1, 2, and 4 h, respectively (75,609, 68,043, 56,605, and 30,388 EB3 comets analyzed); *n* = 31, 31, 30, and 17 TPPP3-KO1 cells at 0, 1, 2, and 4 h, respectively (66,725, 43,038, 34,115, and 18,002 EB3 comets analyzed); *n* = 31, 31, 30, and 17 cells for TPPP3-KO2 cells at 0, 1, 2, and 4 h, respectively (54,462, 46,797, 42,793, and 32,512 EB3 comets analyzed); *n* = 25, 25, 21, and 17 sensitive cells at 0, 1, 2, and 4 h, respectively (29,755, 28,145, 22,020, and 13632 EB3 comets analyzed). For the PBS control, *n* = 30, 30, 30, and 30 resistant cells at 0, 1, 2, and 4 h, respectively (70,276, 70877, 64,407, and 50,741 EB3 comets analyzed); *n* = 37, 37, 35, and 33 TPPP3-KO1 cells at 0, 1, 2, and 4 h, respectively (62,200, 57,133, 40,895, and 34,201 EB3 comets analyzed); *n* = 36, 36, 32, and 32 TPPP3-KO2 cells at 0, 1, 2, and 4 h, respectively (74,188, 73,525, 63,872, and 53,925 EB3 comets); *n* = 27, 27, 27, and 25 sensitive cells at 0, 1, 2, and 4 h, respectively (25,122, 24,807, 23,841, and 19,983 EB3 comets analyzed); n.s., nonsignificant, *p < 0.05, **p < 0.01, ***p < 0.001, ****p < 0.0001, as determined by Mann-Whitney test. (E) Soft-agar cytotoxicity assay of 1A9-activation (1A9-ACT) and 1A9-TPPP3 activated (1A9-TPPP3) cells treated with 2.6 μM cisplatin reveals increased resistance of cisplatin-sensitive cells to cisplatin when TPPP3 is overexpressed. No cisplatin-sensitive cells survive at concentrations higher than 2.6 μM cisplatin. *n* = 3 independent experiments on different days. ***p < 0.0005, ****p < 0.0001, and ns, non-significant, as determined by one-way ANOVA using Tukey’s multiple comparison test. (F) Soft-agar cytotoxicity assay of non-targeting CRISPR-resistant cells (NTCs) and TPPP3-KO cells treated with 2.56 μM cisplatin shows re-sensitization of cisplatin-resistant OVCAR8-CP5 cells to cisplatin when TPPP3 is knockedout. *n* = 3 independent experiments. ****p < 0.0001, ***p < 0.0005, ns, not significant, as determined by one-way ANOVA using Tukey’s multiple comparison test. (G) Overall survival probability curves of 1,409 ovarian cancer patients treated with chemotherapy show poor overall survival probability in patients with high TPPP3 expression. Red and black lines indicate patients with high and low TPPP3 expression, respectivly.

Next, we investigated the effect of TPPP3 on cisplatin resistance in the two TPPP3 knockout cell lines (TPPP3-KO1 and KO2). These showed that TPPP3 depletion eliminates ~75% of the resistance to cisplatin in the resistant cells and reduces colony growth (Figures 6A and S11A–S11C). This is a dramatic effect given that it is likely that several other mechanisms of resistance to cisplatin are operational in these cells. We did not observe any significant change in H2AX (S139) phosphorylation after cisplatin treatment, indicating no change in DNA damage levels upon TPPP3 knockout (Figures S11D and S11E) and suggesting that TPPP3’s role in cisplatin resistance is through a pathway other than DNA repair. Interestingly, we observed a marked reduction in cellular protrusions upon TPPP3 knockout in the resistant cells (Figure S11A), indicating that the changes we observed in microtubule dynamics (Figures S9G and S9H) and cellular morphology are primarily induced by TPPP3. Furthermore, while cisplatin exposure of resistant cells (Figure 6B) or resistant cells expressing the non-targeting guide RNA (gRNA) has small effects on microtubule growth (Figure S12A), the TPPP3-KO cells show pronounced response to the addition of 5 μM cisplatin in the medium (Figure 6C), as do the sensitive (Figure 6D) and re-sensitized cells (Figure S12B). Importantly, ICP-MS analyses show that DNA adducts are present at similar levels in both the control and the TPPP3-KO cells (Figure S12C), indicating that the TPPP3 contribution to resistance is not mediated through direct protection of DNA from cisplatin damage. Furthermore, CRISPR-dCas9 activation of TPPP3 in cisplatin-sensitive cells generates protrusions like those seen in the resistant cells (Figures S13A and S13B) and results in increased colony formation even in the presence of cisplatin (Figure 6E), indicating that expression of TPPP3 alone in sensitive cells can confer cisplatin resistance. We note that increased expression of TPPP3 increases proliferative potential (Figure S13C) but that in addition to this, its expression also increases cisplatin resistance when controlling for proliferation (STAR Methods). The level of resistance induced upon CRISPR-dCas9 activation in the sensitive cells is not the same as in the resistant cells, likely because the TPPP3 expression levels achieved are lower than in the resistant cells, the tubulin isoform repertoire is different, and other mechanisms of resistance are also operational in the resistant cells. We note that higher levels of TPPP3 overexpression were not achievable in the sensitive cells without triggering cell death, suggesting that high TPPP3 levels are not tolerated in the sensitive cells without the broad cytoskeletal pathway rewiring that the resistant cells undergo.

To test the broader applicability of our findings, we also investigated the effects of TPPP3 loss in another cisplatin-resistant ovarian cancer cell line model, OVCAR8-CP5, selected under lower cisplatin concentrations than our original selection scheme (5 μM). OVCAR8-CP5 cells are cisplatin-resistant cells generated from OVCAR8 cells, which are derived from high-grade serous ovarian cancer, the most common type of ovarian cancer. In these cells as well, TPPP3 knockout (Figure S13D; STAR Methods) reduces resistance to cisplatin in a colony growth assay (Figures 6F and S13E). Thus, our TPPP3 activation and ablation results in two different cell line models support the contribution of TPPP3 to the development of cisplatin resistance.

The recovery of cisplatin sensitivity upon TPPP3 depletion suggests that tumors with lower TPPP3 expression levels might show better response to cisplatin therapy. Indeed, through analysis of clinical outcomes of serous ovarian cancer patients,^82,83^ we find that high TPPP3 expression correlates with reduced overall survival probability compared to patients with low TPPP3 expression when treated with platinum-based chemotherapy (*p* = 0.006, *n* = 1,409) (Figure 6G), providing clinical evidence that TPPP3 upregulation correlates with cisplatin resistance in ovarian cancer. Notably, recent single-cell RNA-seq analyses of tissue samples from high-grade serous ovarian cancer tissue obtained before and after platinum-based chemotherapy also associate increased TPPP3 expression with an increased selective advantage during chemotherapy,^84^ consistent with our findings and supporting the potential clinical implications of our work.

## DISCUSSION

Platinum-based chemotherapy was established as the standard of care for ovarian cancer in the mid-1980s.^85,86^ Currently cisplatin is used to treat testicular, ovarian, stomach, and head and neck cancers. While initially effective, cisplatin side effects are severe, in many cases prompting cessation of treatment, and the development of resistance eventually limits its effectiveness. Cisplatin resistance is a multifactorial process, consistent with the high reactivity of cisplatin. Contributing known mechanisms include reduced drug uptake, enhanced DNA repair, as well as emerging evidence suggesting an RNA damage pathway. By reconstituting microtubule dynamics *in vitro*, we now show that cisplatin directly affects microtubule dynamics by increasing tubulin incorporation rates, while at the same time promoting microtubule catastrophe by accelerating the loss of the GTP cap at microtubule ends (Figure 2). The magnitude of the effect of cisplatin on microtubule dynamics is on par with that observed for microtubule-targeting agents. The mechanism of accelerating GTP hydrolysis on tubulin is unclear and could be mediated through changes either to tubulin, to GTP, or both. Cisplatin prefers to bind nitrogen rather than sulfur, and GTP contains an N7 nitrogen that is susceptible to platinum attack as it is in DNA. An early ^195^Pt NMR study using the diaqua form of cisplatin showed that it interacts with the N7 nitrogen of the GTP in the presence of tubulin and proposed that the platinum-bound chelate in the GTP center stimulates hydrolysis.^87^ This proposed mechanism is compatible with our finding that cisplatin increases the rate at which the GTP cap erodes, visualized by examining the decay profile of EB1 comets at microtubule ends (Figures 2G–2J). Future studies will be needed to further understand this process, including whether there are additional interactions of cisplatin with tubulin. Interestingly carboplatin also promotes microtubule catastrophe but without the stimulatory effect on growth rates that cisplatin elicits (Figure S4). These observations should catalyze future mechanistic studies aimed at understanding the effects of different platinum-based compounds on tubulin.

Previous studies using bulk assays arrived at contradictory conclusions regarding the effects of cisplatin on microtubules. Cisplatin was shown to inhibit tubulin incorporation into microtubules^87,88^; however, these studies were conducted at high cisplatin concentrations, exceeding 50 μM.^88^ A later study advanced a model whereby cisplatin inhibits tubulin polymerization by inducing large tubulin aggregates,^87^ while another arrived at the opposite conclusion, namely that cisplatin stabilizes microtubules by protecting them from cold-induced depolymerization, acting through a mechanism similar to Taxol.^89^ However, none of these studies examined microtubule dynamics. Our quantitative measurements of *in vitro* microtubule dynamics now reveal that cisplatin increases microtubule growth rates (and does not decrease them by interfering with tubulin addition as previously suggested^87^), inconsistent with an aggregated tubulin species, while increasing catastrophe rates. By purifying tubulin from ovarian cancer cells, we show that cisplatin-resistant cells stabilize their microtubules (Figure 4) by rewiring their tubulin code through the regulation of tubulin isoforms and post-translational modifications (Figure S8). Furthermore, we show that the tubulin repertoire in the cisplatin-resistant cells (but not in the sensitive or re-sensitized cells) evolved to synergize with TPPP3 to counteract the destabilizing effects of cisplatin (Figures 5 and S10). While changes in tubulin isoforms and post-translational modifications have been known to associate with tumors resistant to microtubule-targeting agents,^65^ this is a direct demonstration that drug-induced changes in the tubulin repertoire are directly responsible for alterations in microtubule dynamics and for opposing the effects of the drug. In addition to connecting changes in microtubule dynamics and cisplatin resistance, our results also underscore the importance of knowing the tubulin isotypes of the cells used for cell biological investigations and of examining the function of microtubule effectors with the relevant tubulin substrate.

High TPPP3 levels correlate with lower survival probability in ovarian cancer patients. Here, we show that in *in vitro* ovarian cancer models, loss of TPPP3 restores partial cisplatin sensitivity, without affecting the DNA damage response (Figures 6, S2, S11D, and S11E). We also show that TPPP3 binds microtubules (Figure S9A) and that its loss impairs microtubule polymerization dynamics (Figures S9G and S9H) and renders microtubules in the resistant cells more sensitive to the effects of cisplatin (Figure 6C). While it remains possible that the effects of TPPP3 on cisplatin resistance are not mediated through its microtubule-binding function, or only partially through it, our findings should catalyze future studies focused on the role of TPPP3 and microtubule-based pathways in cisplatin resistance in ovarian and other cancers. They also suggest TPPP3 degradation as a potential strategy to improve therapeutic outcomes in ovarian cancers (e.g., proteolysis-targeting chimera [PROTAC]^90^ approaches). Notably, TPPP3 is one of the most highly expressed proteins in metastatic fallopian-high-grade serous ovarian tumors.^91^ Therefore, future studies utilizing patient-derived xenograft and animal models are warranted to explore the role of TPPP3 and microtubule-mediated drug resistance in contributing to patient treatment outcomes.

The antagonism between cisplatin and paclitaxel on microtubule dynamics and cisplatin resistance provides a possible mechanism underlying the reported reduced efficacy of these drugs when administered in close succession^92–94^ and the observation that addition of paclitaxel can attenuate the toxicity of platinum compounds used to treat ovarian cancer.^95^ Thus, our results should catalyze a re-examination of the optimal timing in the administration of cisplatin and paclitaxel and the development of treatment resistance. Lastly, the effect of cisplatin on microtubule dynamics that we demonstrate might also have implications for the widespread neuropathy^96–98^ and ototoxicity^99–101^ associated with cisplatin treatment, given the dependence of neurons and the kinocilium on stable and morphologically complex microtubule arrays.

### Limitations of the study

While the precise contribution of TPPP3 to cisplatin resistance through its effect on microtubule dynamics in the context of three-dimensional cell growth will require further investigation, our comprehensive analyses suggest that there is more to be discovered in the field of cisplatin resistance and the drug’s mechanism of action, despite decades of study following the initial characterization of its mechanism of action on DNA. Notably, our data support the existence of a new non-DNA target of cisplatin involved in resistance, opening new avenues for resistance mechanisms involving alternative cellular pathways. Our demonstration that cisplatin directly affects microtubule dynamics, coupled with our transcriptomics analyses showing broad changes in microtubule cytoskeleton-associated genes, suggests that cisplatin imposes selective pressure on the cell through dysregulation of microtubule dynamics. We propose that targeting microtubule effectors in cisplatin resistance, as well as studying how they intersect with other secondary mechanisms of resistance, is worth further investigation.

### RESOURCE AVAILABILITY

#### Lead contact

Further information and requests for resources and reagents should be directed to and will be fulfilled by the lead contact, Sachi Horibata (horibat2@msu.edu).

#### Materials availability

Plasmids and cell lines generated for this study are available upon request from Sachi Horibata (horibat2@msu.edu).

## STAR★METHODS

### EXPERIMENTAL MODEL DETAILS 

1A9 (sensitive) is a single cell clone derived from the A2780 cell line. 1A9CP80 (resistant) cells were generated with gradual exposure to cisplatin up to a final concentration of 80 μM as previously described.^33^ 1A9CP80R (re-sensitized) cells were generated from 1A9CP80 cells removed from cisplatin treatment for approximately one year. The cisplatin-sensitive, -resistant, and partially re-sensitized cells were generous gifts from Dr. Antonio (Tito) Fojo, Columbia University. In this study, we term cisplatin-sensitive, -resistant, and -re-sensitized cells as sensitive, resistant, and re-sensitized cells, respectively. We also used the cisplatin resistant OVCAR8-CP5 cells that were generated with gradual exposure to cisplatin up to a final concentration of 5 μM as previously described.^102^ All cell lines were authenticated as correct and free of mycoplasma contamination by IDEXX BioAnalytics and/or ATCC STR profiling services. Additional mycoplasma testing was conducted in our laboratory every two months using Lonza’s MycoAlert Mycoplasma Detection Assays (Lonza, cat. no. LT07-118) to ensure that cell lines used in our study are free of mycoplasma.

### METHOD DETAILS

#### Cell culture

Cells were cultured in RPMI 1640 containing L-glutamine supplemented with 10% FBS (Atlanta biologicals, cat no. S12450), and 1% penicillin streptomycin (Gibco, cat no. 15140–148). 1A9 cells that were resistant to cisplatin were cultured in the presence of 80 μM cisplatin and OVCAR8-CP5 cells were cultured in the presence of 5 μM cisplatin (*cis-*Diamineplatinum (II) dichloride; CDDP) (Millipore Sigma, cat no. 479306).

#### Cell viability assay

Cell viability assays (Promega, cat no. G7570) were carried out in 96-well white opaque tissue culture plates (FALCON, cat no. 353296) at a seeding density of 5,000 cells per well. The day after seeding, cells were treated with PBS (vehicle control) or with increasing doses of cisplatin for 72 h. It is important to note that we did not dissolve cisplatin in DMSO, as this would inactivate the compound.^111^ For this assay, cisplatin was initially dissolved in PBS at a concentration of 500 μg/mL and was further diluted for the dose-response curves (see Figures S1B and S1C). For Figure 3, in addition to cisplatin, paclitaxel (Millipore Sigma, cat no. T7402) was added at a concentration of 0, 5, or 50 nM. The same amount of DMSO was added for the 0, 5, or 50 nM paclitaxel treatment conditions. While the amount of DMSO was kept at a minimum, because DMSO could interfere with cisplatin activity, it is particularly important to keep the DMSO constant for all treatment conditions, including the 0 nM paclitaxel treatment. This is to ensure that the changes observed are due to changes in paclitaxel concentration and not due to changes in DMSO concentration. The presence of DMSO explains the higher IC_50_ in Figures 3D and 3E compared to Figures S1B and S1C. After 72 h, CellTiter-Glo reagent was added to each well and the cells were incubated for 20 min in the dark at room temperature with gentle shaking. Luminescence was measured using the TECAN infinite M200 PRO reader. CellTiter-Glo measures the level of ATP production of the cells to assess cell viability.

#### CRISPR-knockout

CRISPR-KO of TPPP3 for the 1A9 cell line model was performed using the CRISPR/Cas9 KO Plasmid and HDR plasmid transfection according to manufacturer’s protocol (Santa Cruz). Briefly, 2.5 × 10^6^ resistant cells were plated in 6-well tissue culture plate with 3 mL of RPMI with 10% FBS without any antibiotics. After 24 h, pre-mixed 3 μg of TPPP3 CRISPR/Cas9 KO plasmid (sc-405113), which includes a pool of three different guide RNAs (GCTGACGGAAAGTCCGTGAC; ATCTGCTGGTGTCCGTCAGC; TGCTCGGGTCATCAACTATG), and TPPP3 HDR Plasmid (sc-405113-HRD) or non-targeting control CRISPR/Cas9 plasmid (sc-418922) were transfected into resistant cells using 10 μL of UltraCruz transfection reagent (sc-395739). The TPPP3 CRISPR/Cas9 KO plasmid expressed GFP and the TPPP3 HDR contains puromycin selection. 72 h after transfection cells were selected with 2 μg/mL of puromycin. After puromycin selection, cells were plated at low density and were grown to form colonies in 2D culture. Three different TPPP3-knockout colonies (KO1 and KO2) were then picked and further expanded to be verified using qRT-PCR and Western blot. These showed successful knockout of TPPP3 in KO1 and KO2. Genomic DNA was isolated using the Wizard Genomic DNA Purification Kit (Promega, cat no. A1120). PCR amplification with primers (forward primer GGCTGGGATGTTCTGGGCTTGG, reverse primer TCTTGTAGGCGCTCACGTAGCC) followed by Sanger sequencing showed that the KO1 has complete removal of exon 3 and partially deleted exons 2 and 4, while KO2 has partially deleted exons 3 and 4. The CRISPR mediated TPPP3 KO cell pool for the OVCAR8-CP5 cell line model was generated by Synthego using a single-guide knockout system targeting exon 2 (guide RNA cut location chr16:67, 390, 997). The guide RNA sequence was GAACUGGGCCAAGCUGUGCA. The indel analysis showed successful TPPP3 knockout (score of 96). PCR sequencing primers used: FOR Primer (5′-3′): CCCTGGAAGAACGACCTTCG; REV Primer (5′-3′): CCCAGGTCTTGCAGGTTTGA.

#### CRISPR/dCas9 lentiviral activation

CRISPR/dCas9 lentiviral activation of TPPP3 was performed using the CRISPR/dCas9 lentiviral activation particles according to manufacturer’s protocol (Santa Cruz). Briefly, 2.5 × 10^6^ resistant cells were plated in 6-well tissue culture plate with 3 mL of RPMI with 10% FBS without any antibiotics. After 24 h, 20 μL of TPPP3 lentiviral activation particles (sc-405113-LAC) or control lentiviral activation particles (sc-437282) were transduced into sensitive cells in the presence of polybrene (sc-134220). After 72 h from transduction, cells were selected with 2 μg/mL of puromycin. The pooled population was used for the experiment.

#### Soft agar colony formation assay

Soft agar assays also performed to assess the tumorigenic properties of the cancer cells in the presence or absence of cisplatin. Cisplatin-sensitive, -resistant, and -re-sensitized 1A9 cells were grown in 2-hydroxyethyl agarose (Agarose VII) (Millipore Sigma, cat no. 39346-81-1) as previously described.^112^ Briefly, cisplatin-sensitive, -resistant, and -re-sensitized cells were grown in RPMI 1640 containing L-glutamine supplemented with 10% FBS, 1% penicillin streptomycin, 80 μM of cisplatin, and 0.3% agarose layer grown on top of media containing 0.6% agarose layer in 6-well plate (see Figure S1D). Cells were grown in soft agar matrix suspension for ~3 weeks to measure for their anchorage-independent growth (tumorigenicity).

Non-targeting resistant control (NTC) and TPPP3 knockout cells (TPPP3KO1 and TPPP3KO2) were seeded at 500 cells/well in a 24-well plate in 200 μL mixture comprised of RPMI 1640 media (with L-glutamine, 10% FBS, 1% penicillin streptomycin) and 0.3% agar (Becton Dickinson, cat no. 214010) on top of 350 μL layer comprised of RPMI 1640 media (with L-glutamine, 10% FBS, 1% penicillin streptomycin) and 0.5% agar. The cells were plated in the presence of 25.6 μM cisplatin (*cis*-Diamineplatinum (II) dichloride; CDDP) (Millipore Sigma, cat no. P4394) and were incubated at 37°C for 10 days (see Figures 7A and S10E). This concentration was selected based on the dose-response curve using soft agar (see Figure S10F). After the incubation period, the soft agar plates were fixed using 70% ethanol for 30 min and stained with 200 μL of 0.01% crystal violet. Colonies formed were counted using the Biotek Cytation 3 Imaging Reader (Software program: Gen5 v3.04) within a size range of 50–190 μm and a circularity >0.7.

For 1A9-ACT and 1A9-TPPP3, cells were also seeded at 500 cells/well in a 24-well plate in RPMI 1640 media (with L-glutamine, 10% FBS, 1% PenStrep) and 0.3% agar layer on top of media containing 0.5% agar layer. The cells were plated in the presence of 0 and 2.6 μM cisplatin and incubated at 37°C for 12 days. A 10-fold lower cisplatin concentration was used in this assay because cisplatin-sensitive cells have lower cisplatin sensitivity compared to cisplatin-resistant cells. As shown in Figure S1C, the IC_50_ of cisplatin-sensitive cells is 3 μM when treated in 2D culture for 3 days. This 3D assay was performed for 12 days in the presence of cisplatin and therefore, the lower cisplatin dose was used. After the incubation period, the soft agar plates were fixed using 70% ethanol and stained with 0.01% crystal violet. The object sum area obtained from the Biotek Cytation 3 Imaging Reader (Software program: Gen5 v3.04) was used to calculate colony growth.

Non-targeting control (NTC) and TPPP3 knockout OVCAR8-CP5 cells (TPPP3KO) were seeded at 500 cells/well in a 24-well plate in 200 μL mixture comprised of RPMI 1640 media (with L-glutamine, 10% FBS, 1% penicillin streptomycin) and 0.3% agar (Becton Dickinson, cat no. 214010) on top of 350 μL layer comprised of RPMI 1640 media (with L-glutamine, 10% FBS, 1% penicillin streptomycin) and 0.5% agar. The cells were plated in the presence of 2.56 μM cisplatin (*cis*-Diamineplatinum (II) dichloride; CDDP) (Millipore Sigma, cat no. P4394) and were incubated at 37°C for 12 days. After the incubation period, the soft agar plates were fixed using 70% ethanol for 30 min and stained with 200 μL of 0.01% crystal violet. Colonies formed were counted using the Biotek Cytation 3 Imaging Reader (Software program: Gen5 v3.04) within a size range of 50–190 μm and a circularity >0.7.

#### RNA isolation and sequencing

RNA was isolated from the cells using a RNeasy Kit (Qiagen, cat no. 74104) according to the manufacturer’s protocol. The quality of the RNA was determined using an Agilent D1000 ScreenTape System Bioanalyzer. Only the samples with RNA Integrity Number (RIN) 10 were used for the RNA-seq. All samples were performed in three biological replicates. RNA-seq samples were pooled and sequenced for mRNA on HiSeq3000 with Illumina v4 chemistry using an Illumina TruSeq mRNA Prep Kit and paired-end sequencing (150 base-pair read). Samples were analyzed at the Frederick Sequencing Facility – Center for Cancer Research (CCR), National Cancer Institute (NCI).

#### RNA-seq analysis

Statistical analyses were conducted using R (v3.5). CCBR pipeliner developed by the Center for Cancer Research Collaborative Bioinformatics Resource was used to generate counts file (https://ccbr.github.io/). After applying quantile normalization for log2 transformed expression values, principal component analysis (PCA) showed consistency between replicates as well as distinct separation among the three groups. Differential expression analysis was performed by fitting the generalized linear models (GLM, Gaussian) using *glm* function from R package rms.^109^ The FDR (false discovery rate) was calculated by the Benjamini-Hochberg method for each gene. Genes with FDR <0.01 and |log2 fold-change| > 2 were considered as significantly differentially expressed. Three comparisons were made between sensitive, resistant, and re-sensitized groups, and genes with significant differential expression from all comparisons are shown in the heatmap (Figure 1B) which was generated using the *heatmap.2* function from R package gplots (v3.0.1).^110^ The raw and analyzed data can be found at GEO: GSE320433.

#### Quantitative real-time PCR (qPCR)

RNA was isolated using the RNeasy Mini Kit (Qiagen, cat no. 74104) following manufacturer’s protocol. For polymerase chain reaction (PCR) analysis, 1 μg of total RNA was reverse transcribed using the High-Capacity cDNA Reverse Transcription Kit (Applied Biosystems, cat no. 4368814) to obtain the cDNA. Diluted cDNAs were analyzed by quantitative real-time PCR using the TaqMan primers for TPPP3 (ThermoFisher, Assay ID: Hs00372228_g1) and GAPDH (ThermoFisher, Assay ID: Hs99999905_m1). ΔΔCt method was used for all qPCR analyses. GAPDH was used as a control (see Figures S3C and S9B). For Figure S12A, the diluted cDNAs were analyzed by quantitative real-time PCR using PowerSYBR Green PCR Master Mix (Applied Biosystems, cat no. 4367659). The primer sequences were: TGGGGTTATGGTCCCTGTAA and GTAGAGACCTCCCCACCACA for TPPP3, and TGCACCACCTGCTTAGC and GGCATGGACTGTGGTCATGAG for GAPDH. The gene expression level was normalized to GAPDH.

#### Immunofluorescence

Cells were fixed with 4% paraformaldehyde for 15 min and were permeabilized and blocked with PBS containing 0.1% Triton X-100 and 10% bovine serum albumin. Cells were incubated overnight at 4^◦^C with TPPP3 primary antibodies (Proteintech, cat no. 15057-1-AP) at 1:100 dilution and anti-detyrosinated tubulin (Glu-tubulin) antibodies (Millipore, cat. no. 633710). Cells were rinsed in PBS several times and were incubated with secondary antibody (LI-COR, cat no. 926–32211) containing DAPI. Cells were rinsed and mounted for imaging.

#### Immunoblot assays

Sensitive, resistant, and re-sensitized cells were grown in the absence of cisplatin for at least 2 passages to test the baseline level of TPPP3. Cells were harvested and were re-suspended in lysis buffer containing 50 mM Tris-HCl pH 7.4, 150 mM NaCl, 1% NP-40, 1% protease inhibitor cocktail (Bimake, cat no. B14001) and were sonicated and centrifuged to remove cell debris. Supernatants that contained the cell lysis protein were loaded into a 4–12% gel (ThermoFisher, cat no. NP0321PK2) and subjected to electrophoresis, and transferred to nitrocellulose membrane (VitaScientific, cat no. DBOC80004). The membrane was blocked with Odyssey PBS Blocking Buffer (LICOR, cat no. 927–40000) for 1 h at room temperate and incubated with 1:1000 dilution of anti-TPPP3 antibody (Proteintech, cat no. 15057-1-AP) and 1:1000 dilution of anti-GAPDH antibody (American Research Products, Inc., cat no. 05-50118). GAPDH was used as a loading control.

Cisplatin-sensitive, resistant, and re-sensitized cells were treated with 5 μM cisplatin. Cells were harvested at different time points (0, 2, 4 and 8h) and were re-suspended in lysis buffer containing 50 mM Tris-HCl pH 7.4, 200 mM NaCl, 0.1% Triton X-100, 1mM PMSF, 1% protease inhibitor cocktail and centrifuged to remove cell debris. Supernatants were loaded into a 4–12% gel (ThermoFisher, cat no. NP0321PK2) and were transferred to nitrocellulose membrane. The membranes were treated with 5% non-fat dry milk blocking solution for 1 h at room temperate and were incubated with 1:1000 dilution of mouse monoclonal anti-p53 antibody (Santacruz biotechnology, cat no. SC-126) and 1:1000 dilution of anti-GAPDH antibody (Cell Signaling, cat no.2118L). Cisplatin-sensitive, resistant, re-sensitized, and TPPP3 knock out cisplatin resistant cells were treated with 5 μM cisplatin. Cells were harvested at different time points (0, 2, 4, 8 and 24h) and were re-suspended in lysis buffer containing 50 mM Tris-HCl pH 7.4, 200 mM NaCl, 0.1% Triton X-100, 1mM PMSF, 1% protease inhibitor cocktail and centrifuged to remove cell debris. Supernatants were loaded into a 4–12% gel (ThermoFisher, cat no. NP0321PK2) and were transferred to nitrocellulose membrane. The membranes were treated with 5% non-fat dry milk blocking solution for 1 h at room temperate and were incubated with 1:1000 dilution of rabbit monoclonal anti-phospho H2AX S139 antibody (Cell Signaling, cat no. 9718S) and 1:1000 dilution of anti-GAPDH antibody (Cell Signaling, cat no.97166S). The IRDye 800CW Goat anti-Mouse IgG (LI-COR Biosciences, 926–32210) and IRDye 680LT Goat anti-rabbit (LI-COR Biosciences, 926–68021) secondary antibodies (1:10000 dilution) were used to detect the primary mouse and rabbit antibodies, respectively. Densitometric analyses and quantifications were performed with Image Studio Lite (LI-COR Biosciences) and plotted using GraphPad Prism.

For quantification of TPPP3, 500 ng of recombinant TPPP3 were loaded as a positive control and were run along with lysates from cisplatin-sensitive, -resistant, and -re-sensitized cells. Anti-TPPP3 antibody (1:1000 dilution) was used as a primary antibody as described above. The levels of TPPP3 in cisplatin-sensitive, -resistant, and -re-sensitized cells were calculated based on band intensity referenced to the known concentration of recombinant TPPP3.

#### Inductively coupled mass spectrometry

Inductively coupled mass spectrometry on cell lysates and DNA were performed. Cisplatin-sensitive, resistant, and re-sensitized cells were plated overnight. After 24 h, the cells were treated with 80 μM of cisplatin except for the time value of 0 h. Cell pellets were collected after 2, 4, and 8 h. Cells were lysed using RIPA buffer (Abcam, cat no. ab156034) containing complete mini EDTA free protease inhibitor (Millipore Sigma, cat no. 11836170001). Quantitation of platinum was performed using ICP-MS and normalized in proportion to total protein concentration.

DNA was isolated from cisplatin-resistant non-targeting control (NTC) and TPPP3-KO cells using the Wizard Genomic DNA Purification Kit according to the manufacturer’s protocol (Promega, cat no. A1120). Absorbance at 260nm was used to quantify DNA concentration. The same DNA concentration (20 μg) was used to measure platinum concentration using ICP-MS.

Quantitation of platinum was performed using an Element 2 high resolution ICP-MS (HR-ICP-MS, ThermoFisher Scientific, MA, USA) coupled to a CETAC ASX-520 autosampler (Teladyne/CETAC, NE, USA). Prior to HR-ICP-MS analysis, samples were transferred to 15 mL metal-free conical tubes (Labcon, CA, USA) and digested using 0.125 mL Optima grade nitric acid (70% v/v, Fisher Scientific, MA, USA) for 4 h at 70°C. Following digestion 0.05 mL trace metal grade hydrochloric acid (37% v/v, Fisher Scientific, MA, USA) was added and the samples were diluted with 18.2 MΩ-cm water to up to 3 mL. Platinum standards were made by serially diluting a certified Pt standard (Inorganic Ventures, VA, USA) to 50, 25, 12.5, 6.25, 3.125, 1.5625, and 0.78125 ppb Pt in 4.1% (v/v) Optima grade nitric acid (70% v/v) and 1.7% (v/v) trace metal grade hydrochloric acid matching the acid matrix of all samples and blanks. An internal standard solution was also prepared for in-line internal standardization by diluting a certified multi-element standard containing ^6^Li, Sc, Y, In, Tb, and Bi (IV-ICPMS-71D, Inorganic Ventures, VA, USA) to 2 ppb in the sample matrix. HR-ICP-MS is optimized through manual tuning and validated using a daily performance check and passing manufacturers suggested specifications. After quality control checks, the method for Pt analysis was developed in low resolution (LR) mode by selecting ^195^Pt and ^209^Bi isotopes for analysis (^209^Bi was used for internal standardization), 0.01 s sampling time, 50 samples per peak, Escan, with 4 runs and 4 passes. After sample runs, raw ^195^Pt intensities were normalized to ^209^Bi internal standard intensities and blank subtracted. These corrected intensities were then plotted on the linear regression analysis of the ^195^Pt calibration standards to convert to ppb Pt and normalized in proportion to the total protein concentration.

#### Purification of recombinant TPPP3

*Homo sapiens* TPPP3 was synthesized by Epoch Life Sciences and cloned into pET24a vector for bacterial expression. TPPP3 with a cleavable N-terminal 8xHis tag was expressed in *Escherichia coli* BL21 (DE3) cells. Cultures were grown at 37°C to an OD_600_ of 0.6. Protein expression was induced with 0.5mM IPTG at 16°C for 24 h. Cells were homogenized in ice-cold lysis buffer (50 mM HEPES pH 7.0, 300 mM NaCl, 25 mM Imidazole, and 1mM β-mercaptoethanol) and cell lysate was clarified by centrifugation at 30000xg for 45 min. Cleared cell lysate was loaded onto a Ni^2+^-NTA column and eluted with lysis buffer containing 300 mM imidazole. The eluted protein was treated with TEV protease to remove the His-tag and was dialyzed against a buffer solution containing 50 mM HEPES pH 7.0, 300 mM NaCl, and 1mM DTT at 4°C for 16 h. TPPP3 was further purified on a GE Hiload Superdex 200 gel filtration column (GE Healthcare, cat no. 28-9893-35). Peak fractions were pooled, concentrated, supplemented with glycerol to a final concentration of 15%, flash frozen in liquid nitrogen and stored at −80°C for future use. *Homo sapiens* GFP TPPP1 was synthesized by Epoch Life Sciences and cloned into a pET24A vector for bacterial expression. GFP-TPPP1 with an N-terminal 8xHis tag was expressed and purified like TPPP3. The purified protein was stored at −80° in a buffer solution containing 50 mM HEPES pH 7.0, 300 mM NaCl, 1mM DTT, and 15% glycerol, for future use.

#### Affinity purification of tubulin

Due to the low yield of tubulin isolation from sources other than brain tissue, at least 50 T175 culture flasks were needed for each cell line. Tubulin from the three cancer cells (CP-sensitive, CP-resistant, and CP-re-sensitized) was purified using a TOG domain functionalized resin as previously described.^58,68^ Cells were lysed by sonification in 1XBRB80, pH 6.8 (80 mM PIPES, 1 mM MgCl_2_, 1mM EGTA), 1 mM dithiothreitol (DTT), and 25 μg/mL benzonase. The cell lysate was then cleared by ultracentrifugation at 444,000 x g for 15 min at 4°C. The clarified cell-lysate was loaded on to an N-hydroxysuccinimide (NHS)-column (GE Healthcare, cat no. 17-0716-01) covalently linked to tumor over-expressed gene 1 (TOG1) protein. Tubulin was eluted with 1XBRB80 supplemented with 0.5 M ammonium sulfate. The eluted tubulin was immediately buffer exchanged using a PD-10 column (GE Healthcare, cat no. 17-0851-01) into 1XBRB80, 10% glycerol, and 20 μM GTP, and was flash frozen in liquid nitrogen. The tubulin was further purified by cycling.^113^The cycled tubulin was buffer exchanged using a PD-10 column in 1XBRB80 and 20 μM GTP, flash frozen in liquid nitrogen and stored at −80°C until use. The yield was 0.5 mg for cisplatin-sensitive, 0.4 mg for cisplatin-resistant, and 0.65 mg for cisplatin-resensitized cells.

#### Intact mass spectrometry of purified tubulin

1 μg of purified tubulin mixed with equal volume of 20% acetonitrile (Fisher Scientific, cat no. MAX01566) with 0.1% tri-fluoro acetic acid (TFA) was separated on a Zorbax 300SB C18 column (Agilent, cat no. 5064–8264) using a 0–70% acetonitrile gradient in 0.05% TFA at a flow rate of 0.2 mL/min. The column was attached in line with a 6224 ESI-TOF LC/MS (Agilent), and data were analyzed using the Mass Hunter Workstation platform.

#### Tandem mass spectrometry

50 μg purified tubulin dissolved in 1XBRB80 (80mM Pipes, pH 6.9, 1 mM MgCl2, 1mM EGTA), 10% Glycerol, 10 μM GTP were reduced with 5 mM Tris(2-carboxyethyl)phosphine hydrochloride at RT for 1hr, alkylated with 10 mM N-Ethylmaleimide for 10 min. Half of each sample was digested with trypsin (Trypsin Gold, Mass Spectrometry Grade, Promega, cat no. V5280) and the other half was digested with AspN (Sequencing Grade, Roche, cat no. 11054589001). Trypsin digestion was performed with 1:50 trypsin:sample (w/w) at 37°C for 5 h, followed by 1:25 trypsin:sample (w/w) treatment overnight. AspN digestion was performed with 1:40 AspN:sample (w/w) at 30°C for 5 h, followed by 1:40 AspN:sample (w/w) overnight. Digested samples were desalted using 10mg HLB plate (Waters). The desalted and dried sample was resuspended in 25 μL loading buffer and 1 μL of the sample was injected into HPLC during each LC-MS/MS run. Five technical replicates were acquired for each sample. The data acquisition was performed on a system where an Ultimate 3000 HPLC (Thermo Scientific) was coupled to an Orbitrap Lumos mass spectrometer (Thermo Scientific) via an Easy-Spray ion source (Thermo Scientific). Peptides were separated on an ES911 Easy-Spray column (Thermo Scientific). The composition of mobile phases A and B was 0.1% formic acid in HPLC water, and0.1% formic acid in HPLC acetonitrile, respectively. The mobile B amount was increased from 3% to 22%in 60 min at a flowrate of 300 nL/min. Thermo Scientific Orbitrap Lumos mass spectrometer was operated in data-dependent mode. The MS1 scans were performed in orbitrap with a resolution of 120K at 200 m/z and a mass range of 375–1500 m/z, and an automatic gain control (AGC) value of 2 x 1e5. TheMS2 scans were conducted in ion trap with an AGC target of 3 x 1e4. The MS2 isolation width was 1.6 m/z, and the dynamic exclusion window was 12 s. Proteome Discoverer software version 2.4 was used for protein identification and quantitation. Database search was performed using Sequest HT against Sprot Human database. The mass tolerances for precursor and fragment were set to 5 ppm and 0.4 Da, respectively. Up to 3 missed cleavages were allowed for data obtained from trypsin digestion, and up to 6 missed cleavages were allowed for AspN data. N-Ethylmaleimide (NEM, Millipore Sigma, cat no. 04259) on cysteines was set as fixed modification. Variable modifications include Oxidation (MW), Met-loss (Protein N-term) and Acetyl (Protein N-term). Peptides were validated based on q-values using percolator algorithm. The search results were filtered by a false discovery rate of 1% at the protein level. The protein abundances were calculated by summing abundance of the connected peptides. 1A9 was used as reference during the ratio calculation. Protein ratios were calculated by comparing the median abundance of each group without normalization. The maximum allowed fold change value was 100. ANOVA (Individual Proteins) method was used for hypothesis test. For posttranslational modification analysis, the following variable modifications were added in addition to the modifications mentioned above: Phospho (STY), Gly (E), Glu (E), GluGlu(E), Acetyl (K), DeTyr(C-term). All possible modified peptides found by database search were manually curated.

#### *In vitro* microtubule dynamics assays

GMPCPP-stabilized microtubule seeds (Jena Bioscience, cat no. NU-405) were prepared as previously described.^114^ Neutravidin (Life Technologies, cat no. A-2666) was used to immobilize the microtubule seeds in flow chambers. To study the effect of cisplatin on microtubule dynamics, 10 μM of porcine brain tubulin (Cytoskeleton, cat no. T238P) was treated with varying concentrations of cisplatin (0, 1 μM, 2.5 μM, 5 μM, 10 μM, and 50 μM) and introduced into the flow chambers. The final imaging buffer was BRB80 (80 mM PIPES, pH 6.8, 1 mM MgCl2, 1mM EGTA) supplemented with 50 mM KCl, 1mM GTP, 0.1% methyl cellulose 4,000 cP (Millipore Sigma, cat no. M0512), 1% pluronic F-127 (Invitrogen, cat no. P6866) and 0.1 mg/mL casein (Millipore Sigma, cat no. C5890). An objective heater (Bioptechs) was used to warm the chamber to 30°C and all chambers were sealed and left on the microscope stage for 5 min before imaging. Interference Reflection Microscopy (IRM) images were acquired on a Nikon Eclipse Ti-E equipped with a Hamamatsu ORCA Flash4.0 Vc sCMOS camera every 5s for 40 or 50 min. To determine the effects of carboplatin on microtubule dynamics, 10 μM porcine brain tubulin was incubated with varying concentrations of carboplatin (50 μM and 100 μM carboplatin; Millipore Sigma, cat no. C2538). IRM images were obtained as described above.

The dynamics of microtubules polymerized from tubulin purified from the cisplatin-sensitive, -resistant and -re-sensitized cells were measured at 6 μM tubulin concentration and the IRM images were obtained as described above. Microtubule dynamics measurements were performed in the presence of 1 μM TPPP3 and 5 μM cisplatin, separately and in combination. Dynamicity was determined as the sum of total growth and shortening lengths divided by total time as defined in.^115^ To determine the effects of cisplatin and paclitaxel, 6 μM of porcine brain tubulin was treated with varying concentrations of paclitaxel (10 nM, 20 nM, and 100nM; Millipore Sigma, T7402), separately and in combination with 5 μM cisplatin. IRM images were obtained as described above.

#### Quantification of microtubule dynamics

The dynamic parameters of microtubules were measured in ImageJ^104^ as previously described.^59,108^ Briefly, kymographs were generated from the IRM images and hand traced to obtain dynamic parameters. Growth rates were determined from the slope of the growing microtubules. Catastrophe rates were calculated as the number of catastrophes observed divided by the total time spent in the growth phase. Rescue events were calculated as the number of rescues observed divided by the total time spent in the depolymerization phase. Microtubule length is microtubule length before a catastrophe.

#### Live cell EB3 dynamics

Live cells expressing EB3-mScarlet treated with oxyrace (Oxyrace, Inc, Mansfield, OH, cat no. OF-0005) were observed with a Nikon Ti-E microscope equipped with a 60× objective, 1.49 numerical aperture lens coupled to a Yokogawa CSU-X1 spinning disc confocal scan head. Images were acquired using a Hamamatsu ORCA-Flash4.0 version 2 camera. Time-lapse sequences of EB3-mScarlet were collected with an exposure time of 100ms at an interval of 2 s for a total time of 10 min. The EB3 comets (Addgene, 98826)^103^ on the microtubule plus-ends were detected and tracked using the comet detection and plus-end tracking modules in the open source, uTrack v2.1.3 software.^106,107^ For this purpose, the pixel size (120 nm), frame interval (2 s), numerical aperture (1.49 NA), camera depth (16) was specified. Default parameters were used for band-pass filter and watershed segmentation. Identical image acquisition settings and parameters were used for all datasets quantified. Microtubule tracking was performed using the MT plus-end tracing module of uTrack, which links the comets of consecutive frames. The growth rate and the track lengths measured by the module was plotted using GraphPad Prism 9.

#### Microtubule nucleation assay

Flow chambers were made using silanized coverglass and double stick tape.^116^ Microtubule nucleation was observed by introducing 10 μM of porcine brain tubulin in the presence of either 5 μM TPPP1 or 20 μM TPPP3. The final imaging buffer is BRB80 (80 mM PIPES, pH 6.8, 1 mM MgCl_2_, 1mM EGTA) supplemented with 50 mM KCl, 1mM GTP, 0.1% methylcellulose 4,000 cP, 1% pluronic F-127 and 0.1 mg/mL casein. The chambers were warmed to 30°C using an objective heater (Bioptechs) before imaging. Interference Reflection Microscopy (IRM) images were acquired on a Nikon Eclipse Ti-E equipped with a Hamamatsu ORCA Flash4.0 Vc sCMOS camera every 5s for 20 min.

#### Microtubule co-sedimentation assay

Sedimentation assays were performed in BRB80 buffer (80 mM PIPES, pH 6.8, 1 mM MgCl_2_, 1mM EGTA) and 1 mM DTT. 10μM TPPP3 was added to different concentrations of paclitaxel-stabilized porcine brain microtubules. The mixture was incubated for 20 min and then centrifuged at 100000 g for 10 min at 30°C. The supernatant was collected, and the pellets were resolubilized in 1X BRB80 buffer. Equivalent amounts of the supernatant and pellet fractions were analyzed by SDS-PAGE. Images of the Coomassie blue stained gels were analyzed using Image StudioLite software package from Licor and the relative amounts of protein in the supernatant and pellet fractions were quantified. The fraction of the protein bound to the microtubules were plotted as a function of microtubule concentration. The K_d_ values were obtained by fitting the data to a one-site binding parameter in GraphPad Prism 9.

#### *In vitro* TIRF with EB1 and comet analysis

Microtubules were assembled and polymerized using GMPCPP stabilized microtubule seeds at 13 μM tubulin in the presence of 300 nM EB1-GFP, with and without cisplatin. The final imaging buffer contained 1XBRB80, pH 6.8, supplemented with 50 mM KCl, 1 mM GTP, 1% pluronic F-127, 100 μg/mL κ-casein, 1 mM β-mercaptoethanol, and oxygen scavengers. An objective heater (Bioptechs) was used to heat the chamber to 30°C. Synchronous TIRF/IRM imaging was performed on a Nikon Eclipse Ti-E equipped with a LED light source, 488-laser and EMCCD cameras (Andor) with an exposure time of 200 ms per frame. Images were collected at 1 frame per second for 10 min. The fluorescence signal of microtubule plus-end comets was analyzed as previously described.^58,117^ Briefly, kymographs of growing plus-end microtubules were drawn in ImageJ. A custom-written MATLAB script (https://github.com/RollmecakLab) was used to identify the maximum intensity in each line of the kymograph. Then an exponential fit to the line profile covering 1.5 μm of the lattice and a Gaussian fit to the line profile extending 1.5 μm beyond the microtubule tip was performed. For all exponential and Gaussian fitting, *R*^2^ > 0.99. This was repeated for all kymographs. All fitted comet profiles were aligned, binned and their average values were calculated. The averaged comet profile was then fitted to a single exponential fit from the comet peak to the end of the comet with an offset that represents the lattice intensity. These comet profiles were then plotted in Prism and background corrected by subtracting the minimum background value. The reported comet intensity is the averaged peak value after background correction, and the lattice intensity is the background-corrected offset value, determined by the exponential fitting of comet tail.

#### TIRF-based microtubule binding assays

Paclitaxel-stabilized cisplatin sensitive (CP-S) microtubules containing 97% CP-S tubulin, 1% biotin brain tubulin, and 2% 647-Hilyte tubulin (Cytoskeleton, cat no. TL670M-B) and paclitaxel-stabilized cisplatin resistant (CP-R) microtubules containing 99% CP-R tubulin, 1% biotin brain tubulin were immobilized on a glass surface using neutravidin (Life Technologies, A-2666). Similarly, paclitaxel-stabilized cisplatin resensitized (CP-RS) microtubules containing 97% CP-RS tubulin, 1% biotin brain tubulin, and 2% 647-Hilyte tubulin and paclitaxel-stabilized cisplatin resistant (CP-R) microtubules containing 99% CP-R tubulin, 1% biotin brain tubulin (Cytoskeleton, cat no. T333P) were immobilized on a glass surface using neutravidin. TPPP3 at 200nM containing 20% TPPP3-GFP was perfused into the chamber in assay buffer (80 mM PIPES6.9, 1 mM MgCl_2_, 1 mM EGTA, 50 mM KCl) supplemented with 1% F127 pluronic acid, 20 μM paclitaxel and oxygen scavengers (7.5 U/μL catalase (Millipore Sigma, cat. no 9001-05-02), 0.15 U/μL glucose oxidase (Millipore Sigma, cat no. G7141-250KU), 20 mM glucose (Millipore Sigma, cat no. G8270-1KG)^116^ to remove free oxygen from the solution. An inverted TIRF microscope (Nikon Ti-E with TIRF attachment) was used for image acquisition. The excitation light was provided by a 640 nm Coherent CUBE for Hylite-647 and a 488 nm Coherent CUBE for TPPP3-GFP both operating at 20 mW before being coupled to the TIRF attachment via a fiber optic. The excitation light was focused at the back focal plane of a 100X 1.49 NA objective (Nikon CFI Apo TIRF 100X). The excitation light was split from the emission light using a 405/488/532/635 quad band filter (Semrock DiO3-405/488/532/635) and directed to the sample. The emitted light was collected by the same objective and split using an Andor TuCAM equipped with a dichroic mirror (Semrock FF640-FDi02). EMCCD cameras (Andor iXON3-897) equipped with either an FF01-550/88 (Semrock) or a BLP01-647R (Semrock) were used to image the 488 and 640 channels respectively. Microtubule images were obtained using interference reflection microscopy on an ORCA Flash4.0 V2 sCMOS camera (Hamamatsu) before acquiring fluorescent images. Multiple fields of view were imaged. Background corrected line scan intensities were measured using FIJI^105^ and intensities were normalized to microtubule length. To eliminate any confounding effects of 647-Hilyte tubulin on TPPP3 binding, the experiments were performed with cisplatin resistant microtubules containing 2% 647-Hilyte tubulin, and unlabeled CP-S and CP-RS microtubules. The data were collected and analyzed as described above.

#### Overall patient survival probability

Gene expression and survival information of ovarian cancer patients from the Gene Expression Omnibus and The Cancer Genome Atlas (Affymetrix HG-U133A, HG-U133A 2.0, and HG-U133 Plus 2.0 microarrays) incorporated in the online tool, www.kmplot.com/ovar,^83^ were used to calculate the overall survival probability of ovarian cancer patients based on TPPP3 expression (Affymetrix ID: 218876_at). Patients were selected based on those treated with platinum-containing chemotherapy (*n* = 1,435) of which, 1,409 were available for analysis. Patients were split using the auto select best cutoff^82^ based on TPPP3 expression and were not restricted based on their histology stage, grade TP53 mutation status, or debulking procedure.

### QUANTIFICATION AND STATISTICAL ANALYSIS

Statistical parameters such as independent experiments (n), standard deviation (mean ± SD), standard error of the mean (mean ± SEM), statistical significance (p-value or FDR) and statistical tests used are either shown in the figure or indicated in the figure legends. Statistical analyses were performed with GraphPad PRISM 9.

## Supplementary Material

1

2

3

4

5

6

7

8

Supplemental information can be found online at https://doi.org/10.1016/j.celrep.2026.117414.

## Figures and Tables

**Table:** KEY RESOURCES TABLE

REAGENT or RESOURCE	SOURCE	IDENTIFIER
Antibodies		
Rabbit anti-TPPP3 polyclonal antibody	Proteintech	Cat#15057-1-AP; Lot#0014931
Rabbit anti-Tubulin, detyrosinated, polyclonal antibody	Millipore	Cat#633710
Mouse anti-GAPDH monoclonal antibody	American Research Products	Cat#05-50118
Mouse anti-GAPDH monoclonal antibody	Cell Signaling	Cat#97166S
Rabbit anti-GAPDH monoclonal antibody	Cell Signaling	Cat#2118L
Rabbit anti-phospho H2AX S139 monoclonal antibody	Cell Signaling	Cat#9718S
Mouse anti-p53 monoclonal antibody	Santa Cruz Biotechnology	Cat#sc-126
IRDye 800CW Goat anti-Rabbit IgG	LI-COR Biosciences	Cat#926-32211
IRDye 680LT Goat anti-Rabbit IgG	LI-COR Biosciences	Cat# 926-68021
IRDye 800CW Goat anti-Mouse IgG	LI-COR Biosciences	Cat# 926-32210
Bacterial and virus strains		
TPPP3 lentiviral activation particles	Santa Cruz Technology	Cat#sc-405113-LAC
Control lentiviral activation particles	Santa Cruz Technology	Cat#sc-437282
Chemicals, peptides, and recombinant proteins		
Cisplatin (*cis*-Diamineplatinum (II) dichloride; CDDP)	Millipore Sigma	Cat#479306
Paclitaxel	Millipore Sigma	Cat#T7402 (Alt: Cat#T1912)
Carboplatin	Millipore Sigma	Cat#C2538
2-hydroxyethyl agarose (Agarose VII)	Millipore Sigma	Cat#39346-81-1
Porcine brain tubulin	Cytoskeleton	Cat#T238P
Tubulin 647-HiLyte	Cytoskeleton	Cat#TL670M-B
Biotin Labeled tubulin	Cytoskeleton	Cat#T333P
Trypsin Gold, Mass Spectrometry Grade	Promega	Cat# V5280
AspN, Sequencing Grade	Roche	Cat#11054589001
Oxyrace	Oxyrace, Inc, Mansfield, OH	Cat#OF-0005
Neutravidin	Life Technologies	Cat#A-2666 (Alt: Cat#31000)
Acetonitrile (LC-MS grade)	Fisher Scientific	Cat#MAX01566
N-Ethylmaleimide (NEM)	Millipore Sigma	Cat#04259
Glucose Oxidase from *Aspergillus niger*	Millipore Sigma	Cat#G7141-250KU
Catalase from bovine liver	Millipore Sigma	Cat#9001-05-02
D-(+)-Glucose	Millipore Sigma	Cat#G8270-1KG
Methyl cellulose 4000 cp	Sigma Aldrich	Cat#M0512
Pluronic F127	Invitrogen	Cat#P6866
GMPCPP	Jena Bioscience	Cat# NU-405
Casein	Sigma-Aldrich	Cat# C5890
Critical commercial assays		
CellTiter-Glo Luminescent Cell Viability Assay	Promega	Cat#G7570
Wizard Genomic DNA Purification Kit	Promega	Cat#A1120
RNeasy Kit	Qiagen	Cat#74104
Deposited data		
Raw and analyzed data	This paper	GEO: GSE320433
Experimental models: Cell lines		
1A9, cisplatin-sensitive	Laboratory of Antonio Fojo (Vandier et al.^33^ 2000)	N/A
1A9CP80, cisplatin-resistant	Laboratory of Antonio Fojo (Vandier et al.^33^ 2000)	N/A
1A9CP80R, cisplatin-re-sensitized	Laboratory of Antonio Fojo	N/A
OVCAR8	National Cancer Institute	NCI-60
OVCAR8-CP5	Laboratory of Michael Gottesman (Patel et al.^102^ 2021)	N/A
Oligonucleotides		
Amplification Primer: Forward: GGCTGGGATGTTCTGGGCTTGG; Reverse: TCTTGTAGGCGCTCACGTAGCC	This paper	N/A
Primer: Forward: CCCTGGAAGAACGACCTTCG; Reverse: CCCAGGTCTTGCAGGTTTGA	This paper	N/A
TPPP3 TaqMan Primer	ThermoFisher	Assay ID: Hs00372228_g1
GAPDH TaqMan Primer	ThermoFisher	Assay ID: Hs99999905_m1
TPPP3 Primer Pairs for PowerSYBR Green: TGGGGTTATGGTCCCTGTAA; GTAGAGACCTCCCCACCACA	This paper	N/A
GAPDH Primer Pairs PowerSYBR Green: TGCACCACCTGCTTAGC; GGCATGGACTGTGGTCATGAG	This paper	N/A
Recombinant DNA		
TPPP3 CRISPR/Cas9 KO plasmid (h)	Santa Cruz Biotechnology	Cat#sc-405113
TPPP3 HDR Plasmid (h)	Santa Cruz Biotechnology	Cat#sc-405113-HDR
Control CRISPR/Cas9 Plasmid	Santa Cruz Biotechnology	Cat#sc-418922
Human TPPP3-GFP	Gene Synthesized	N/A
Human TPPP1-GFP	Gene Synthesized	N/A
EB3-mscarlet	Chertkova et al.^103^ 2020	Addgene Plasmid #98826
Software and algorithms		
Biotek Gen5 v3.04	N/A	N/A
GraphPad Prism v.11.0.0	N/A	N/A
ImageJ	Schneider et al.^104^ 2012	https://www.imagej.net
FIJI	Schindelin et al.^105^ 2012	https://www.imagej.net
Image StudioLite	LICORbio	https://www.licorbio.com/image-studio
uTrack v2.1.3 software	Applegate et al.^106^ 2011 Jaqaman et al.^107^ 2008	https://github.com/DanuserLab/u-track
EB comet analysis	Chen et al.^108^ 2021	(https://github.com/RollmecakLab)
Regression modeling strategies (rms)	Harrell Jr, F.^109^ 2026	https://cran.r-project.org/web/packages/rms/
gplots	Warnes et al.^110^ 2025	https://github.com/talgalili/gplots
Ccbrpipeliner	CCR Collaborative Bioinformatics Resource	https://ccbr.github.io/
Proteome Discoverer Software	Thermo Fischer	N/A
Other		
GE Hiload Superdex 200 gel filtration column	GE Healthcare	Cat#28-9893-35
N-hydroxysuccinimide (NHS)-column	GE Healthcare	Cat#17-0716-01
PD-10 column	GE Healthcare	Cat#17-0851-01
Zorbax 300SB C18 column	Agilent	Cat#5064-8264

## Data Availability

The raw and analyzed RNA-seq count data were deposited in GEO: GSE320433. Differential gene expression data are provided in Data S1, S2, S3, and S4. EB comet analysis uses custom code, which is freely available at https://github.com/RollmecakLab. All other analysis programs are publicly available, as outlined in the key resources table.

## Highlights

- Cisplatin increases microtubule growth rates and promotes catastrophe
- Cisplatin resistance rewires the tubulin code to stabilize microtubules
- Cisplatin counteracts microtubule stabilization by paclitaxel, leading to resistance
- TPPP3 synergizes with tubulin isotypes and contributes to resistance

## References

[1] Galluzzi L, Senovilla L, Vitale I, Michels J, Martins I, Kepp O, Castedo M, and Kroemer G (2012). Molecular mechanisms of cisplatin resistance. Oncogene 31, 1869–1883. 10.1038/onc.2011.384. 21892204

[2] Hall MD, Okabe M, Shen DW, Liang XJ, and Gottesman MM. (2008). The role of cellular accumulation in determining sensitivity to platinum-based chemotherapy. Annu. Rev. Pharmacol. Toxicol. 48, 495–535. 10.1146/annurev.pharmtox.48.080907.180426. 17937596

[3] el-Khateeb M, Appleton TG, Gahan LR, Charles BG, Berners-Price SJ, and Bolton AM (1999). Reactions of cisplatin hydrolytes with methionine, cysteine, and plasma ultrafiltrate studied by a combination of HPLC and NMR techniques. J. Inorg. Biochem. 77, 13–21. 10.1016/s0162-0134(99)00146-4. 10626348

[4] Kelland LR. (2000). Preclinical perspectives on platinum resistance. Drugs 59, 1–38, [discussion: 37-38]. 10.2165/00003495-200059004-00001. 10864225

[5] Eastman A. (1987). The formation, isolation and characterization of DNA adducts produced by anticancer platinum complexes. Pharmacol. Ther. 34, 155–166. 10.1016/0163-7258(87)90009-x. 3317449

[6] Timerbaev AR, Hartinger CG, Aleksenko SS, and Keppler BK. (2006). Interactions of antitumor metallodrugs with serum proteins: advances in characterization using modern analytical methodology. Chem. Rev. 106, 2224–2248. 10.1021/cr040704h. 16771448

[7] Dasari S, and Tchounwou PB. (2014). Cisplatin in cancer therapy: molecular mechanisms of action. Eur. J. Pharmacol. 740, 364–378. 10.1016/j.ejphar.2014.07.025. PMC4146684 25058905

[8] Köoberle B, Tomicic MT, Usanova S, and Kaina B. (2010). Cisplatin resistance: preclinical findings and clinical implications. Biochim. Biophys. Acta 1806, 172–182. 10.1016/j.bbcan.2010.07.004. 20647037

[9] Agarwal R, and Kaye SB. (2003). Ovarian cancer: strategies for overcoming resistance to chemotherapy. Nat. Rev. Cancer 3, 502–516. 10.1038/nrc1123. 12835670

[10] Kim A, Ueda Y, Naka T, and Enomoto T. (2012). Therapeutic strategies in epithelial ovarian cancer. J. Exp. Clin. Cancer Res. 31, 14. 10.1186/1756-9966-31-14. PMC3309949 22330607

[11] Wang X, Wang E, Kavanagh JJ, and Freedman RS. (2005). Ovarian cancer, the coagulation pathway, and inflammation. J. Transl. Med. 3, 25. 10.1186/1479-5876-3-25. PMC1182397 15969748

[12] Shen DW, Pouliot LM, Hall MD, and Gottesman MM. (2012). Cisplatin resistance: a cellular self-defense mechanism resulting from multiple epigenetic and genetic changes. Pharmacol. Rev. 64, 706–721. 10.1124/pr.111.005637. PMC3400836 22659329

[13] Holzer AK, Samimi G, Katano K, Naerdemann W, Lin X, Safaei R, and Howell SB. (2004). The copper influx transporter human copper transport protein 1 regulates the uptake of cisplatin in human ovarian carcinoma cells. Mol. Pharmacol. 66, 817–823. 10.1124/mol.104.001198. 15229296

[14] Mangala LS, Zuzel V, Schmandt R, Leshane ES, Halder JB, Armaiz-Pena GN, Spannuth WA, Tanaka T, Shahzad MMK, Lin YG, (2009). Therapeutic Targeting of ATP7B in Ovarian Carcinoma. Clin. Cancer Res. 15, 3770–3780. 10.1158/1078-0432.CCR-08-2306. PMC2752981 19470734

[15] Safaei R, Otani S, Larson BJ, Rasmussen ML, and Howell SB. (2008). Transport of cisplatin by the copper efflux transporter ATP7B. Mol. Pharmacol. 73, 461–468. 10.1124/mol.107.040980. 17978167

[16] Samimi G, Safaei R, Katano K, Holzer AK, Rochdi M, Tomioka M, Goodman M, and Howell SB. (2004). Increased expression of the copper efflux transporter ATP7A mediates resistance to cisplatin, carboplatin, and oxaliplatin in ovarian cancer cells. Clin. Cancer Res. 10, 4661–4669. 10.1158/1078-0432.CCR-04-0137. 15269138

[17] Zamble DB, and Lippard SJ. (1995). Cisplatin and DNA repair in cancer chemotherapy. Trends Biochem. Sci. 20, 435–439. 10.1016/s0968-0004(00)89095-7. 8533159

[18] Fox M, and Roberts JJ. (1987). Drug resistance and DNA repair. Cancer Metastasis Rev. 6, 261–281. 10.1007/BF00144267. 3319272

[19] Furuta T, Ueda T, Aune G, Sarasin A, Kraemer KH, and Pommier Y. (2002). Transcription-coupled nucleotide excision repair as a determinant of cisplatin sensitivity of human cells. Cancer Res. 62, 4899–4902. 12208738

[20] Kunkel TA, and Erie DA. (2005). DNA mismatch repair. Annu. Rev. Biochem. 74, 681–710. 10.1146/annurev.biochem.74.082803.133243. 15952900

[21] Fuertes MA, Castilla J, Alonso C, and Pérez JM. (2002). Novel concepts in the development of platinum antitumor drugs. Curr. Med. Chem. Anticancer. Agents 2, 539–551. 10.2174/1568011023353958. 12678734

[22] Gonzalez VM, Fuertes MA, Alonso C, and Perez JM. (2001). Is cisplatin-induced cell death always produced by apoptosis? Mol. Pharmacol. 59, 657–663. 10.1124/mol.59.4.657. 11259608

[23] Chen HH, and Kuo MT. (2010). Role of glutathione in the regulation of Cisplatin resistance in cancer chemotherapy. Met Based Drugs 2010, 430939. 10.1155/2010/430939. PMC2946579 20885916

[24] Berners-Price SJ, and Kuchel PW. (1990). Reaction of cis- and trans-[PtCl2(NH3)2] with reduced glutathione inside human red blood cells, studied by 1H and 15N-[1H] DEPT NMR. J. Inorg. Biochem. 38, 327–345. 10.1016/0162-0134(90)80006-j. 2332767

[25] Bose RN, Ghosh SK, and Moghaddas S. (1997). Kinetic analysis of the cis-diamminedichloroplatinum(II)–cysteine reaction: implications to the extent of platinum–DNA binding. J. Inorg. Biochem. 65, 199–205. 10.1016/s0162-0134(96)00133-x. 9025271

[26] Dabrowiak JC, Goodisman J, and Souid AK. (2002). Kinetic study of the reaction of cisplatin with thiols. Drug Metab. Dispos. 30, 1378–1384. 10.1124/dmd.30.12.1378. 12433807

[27] Si M, and Lang J. (2018). The roles of metallothioneins in carcinogenesis. J. Hematol. Oncol. 11, 107. 10.1186/s13045-018-0645-x. PMC6108115 30139373

[28] Rose F, Köoberle B, Honnen S, Bay C, Burhenne J, Weiss J, Haefeli WE, and Theile D. (2024). RNA is a pro-apoptotic target of cisplatin in cancer cell lines and C. elegans. Biomed. Pharmacother. 173, 116450. 10.1016/j.biopha.2024.116450. 38503239

[29] Fitzpatrick DPG, You JS, Bemis KG, Wery JP, Ludwig JR, and Wang M. (2007). Searching for potential biomarkers of cisplatin resistance in human ovarian cancer using a label-free LC/MS-based protein quantification method. Proteomics. Clin. Appl. 1, 246–263. 10.1002/prca.200600768. 21136676

[30] Parker AL, Teo WS, McCarroll JA, and Kavallaris M. (2017). An Emerging Role for Tubulin Isotypes in Modulating Cancer Biology and Chemotherapy Resistance. Int. J. Mol. Sci. 18, 1434. 10.3390/ijms18071434. PMC5535925 28677634

[31] Roll-Mecak A. (2020). The Tubulin Code in Microtubule Dynamics and Information Encoding. Dev. Cell 54, 7–20. 10.1016/j.devcel.2020.06.008. PMC11042690 32634400

[32] MacTaggart B, and Kashina A. (2021). Posttranslational modifications of the cytoskeleton. Cytoskeleton (Hoboken) 78, 142–173. 10.1002/cm.21679. PMC8856578 34152688

[33] Vandier D, Calvez V, Massade L, Gouyette A, Mickley L, Fojo T, and Rixe O. (2000). Transactivation of the metallothionein promoter in cisplatin-resistant cancer cells: a specific gene therapy strategy. J. Natl. Cancer Inst. 92, 642–647. 10.1093/jnci/92.8.642. 10772682

[34] Elaimy AL, Amante JJ, Zhu LJ, Wang M, Walmsley CS, FitzGerald TJ, Goel HL, and Mercurio AM. (2019). The VEGF receptor neuropilin 2 promotes homologous recombination by stimulating YAP/TAZ-mediated Rad51 expression. Proc. Natl. Acad. Sci. USA 116, 14174–14180. 10.1073/pnas.1821194116. PMC6628806 31235595

[35] Reader JC, Fan C, Ory ECH, Ju J, Lee R, Vitolo MI, Smith P, Wu S, Ching MMN, Asiedu EB, (2022). Microtentacle Formation in Ovarian Carcinoma. Cancers (Basel) 14, 800. 10.3390/cancers14030800. PMC8834106 35159067

[36] Nusinow DP, Szpyt J, Ghandi M, Rose CM, McDonald ER 3rd, Kalocsay M, Jané-Valbuena J, Gelfand E, Schweppe DK, Jedrychowski M, (2020). Quantitative Proteomics of the Cancer Cell Line Encyclopedia. Cell 180, 387–402.e16. 10.1016/j.cell.2019.12.023. PMC7339254 31978347

[37] Kaverina I, and Straube A. (2011). Regulation of cell migration by dynamic microtubules. Semin. Cell Dev. Biol. 22, 968–974. 10.1016/j.semcdb.2011.09.017. PMC3256984 22001384

[38] Heald R, and Khodjakov A. (2015). Thirty years of search and capture: The complex simplicity of mitotic spindle assembly. J. Cell Biol. 211, 1103–1111. 10.1083/jcb.201510015. PMC4687881 26668328

[39] Rajkumar P, Mathew BS, Das S, Isaiah R, John S, Prabha R, and Fleming DH. (2016). Cisplatin Concentrations in Long and Short Duration Infusion: Implications for the Optimal Time of Radiation Delivery. J. Clin. Diagn. Res. 10, XC01–XC04. 10.7860/JCDR/2016/18181.8126. PMC5020194 27630935

[40] Liston DR, and Davis M. (2017). Clinically Relevant Concentrations of Anticancer Drugs: A Guide for Nonclinical Studies. Clin. Cancer Res. 23, 3489–3498. 10.1158/1078-0432.CCR-16-3083. PMC5511563 28364015

[41] Falta T, Heffeter P, Mohamed A, Berger W, Hann S, and Koellensperger G. (2011). Quantitative determination of intact free cisplatin in cell models by LC-ICP-MS. J. Anal. At. Spectrom. 26, 109–115.

[42] Dhamodharan R, Jordan MA, Thrower D, Wilson L, and Wadsworth P. (1995). Vinblastine suppresses dynamics of individual microtubules in living interphase cells. Mol. Biol. Cell 6, 1215–1229. 10.1091/mbc.6.9.1215. PMC301278 8534917

[43] Yvon AM, Wadsworth P, and Jordan MA. (1999). Taxol suppresses dynamics of individual microtubules in living human tumor cells. Mol. Biol. Cell 10, 947–959. 10.1091/mbc.10.4.947. PMC25218 10198049

[44] Bringmann H, Skiniotis G, Spilker A, Kandels-Lewis S, Vernos I, and Surrey T. (2004). A kinesin-like motor inhibits microtubule dynamic instability. Science 303, 1519–1522. 10.1126/science.1094838. 15001780

[45] Dhamodharan R, and Wadsworth P. (1995). Modulation of microtubule dynamic instability in vivo by brain microtubule associated proteins. J. Cell Sci. 108, 1679–1689. 10.1242/jcs.108.4.1679. 7615685

[46] Lawrence EJ, Arpag G, Arnaiz C, and Zanic M. (2021). SSNA1 stabilizes dynamic microtubules and detects microtubule damage. Elife 10, e67282. 10.7554/eLife.67282. PMC8798045 34970964

[47] Hoff KJ, Aiken JE, Gutierrez MA, Franco SJ, and Moore JK. (2022). TUBA1A tubulinopathy mutants disrupt neuron morphogenesis and override XMAP215/Stu2 regulation of microtubule dynamics. Elife 11, e76189. 10.7554/eLife.76189. PMC9236607 35511030

[48] Tischfield MA, Baris HN, Wu C, Rudolph G, Van Maldergem L, He W, Chan WM, Andrews C, Demer JL, Robertson RL, (2010). Human TUBB3 mutations perturb microtubule dynamics, kinesin interactions, and axon guidance. Cell 140, 74–87. 10.1016/j.cell.2009.12.011. PMC3164117 20074521

[49] Oguri S, Sakakibara T, Mase H, Shimizu T, Ishikawa K, Kimura K, and Smyth RD. (1988). Clinical pharmacokinetics of carboplatin. J. Clin. Pharmacol. 28, 208–215. 10.1002/j.1552-4604.1988.tb03134.x. 3283185

[50] Wang S, Zhang H, Malfatti M, de Vere White R, Lara PN Jr., Turteltaub K, Henderson P, and Pan C.x. (2010). Gemcitabine causes minimal modulation of carboplatin-DNA monoadduct formation and repair in bladder cancer cells. Chem. Res. Toxicol. 23, 1653–1655. 10.1021/tx1003547. PMC2987236 21028869

[51] Mitchison T, and Kirschner M. (1984). Dynamic instability of microtubule growth. Nature 312, 237–242. 10.1038/312237a0. 6504138

[52] Carlier MF, and Pantaloni D. (1982). Assembly of microtubule protein: role of guanosine di- and triphosphate nucleotides. Biochemistry 21, 1215–1224. 10.1021/bi00535a017. 7074077

[53] Saltarelli D, and Pantaloni D. (1982). Polymerization of the tubulin-colchicine complex and guanosine 5’-triphosphate hydrolysis. Biochemistry 21, 2996–3006. 10.1021/bi00541a030. 7104309

[54] Roostalu J, Thomas C, Cade NI, Kunzelmann S, Taylor IA, and Surrey T. (2020). The speed of GTP hydrolysis determines GTP cap size and controls microtubule stability. Elife 9, e51992. 10.7554/eLife.51992. PMC7018511 32053491

[55] Maurer SP, Fourniol FJ, Bohner G, Moores CA, and Surrey T. (2012). EBs recognize a nucleotide-dependent structural cap at growing microtubule ends. Cell 149, 371–382. 10.1016/j.cell.2012.02.049. PMC3368265 22500803

[56] Parmar MKB, Ledermann JA, Colombo N, du Bois A, Delaloye JF, Kristensen GB, Wheeler S, Swart AM, Qian W, Torri V, (2003). Paclitaxel plus platinum-based chemotherapy versus conventional platinum-based chemotherapy in women with relapsed ovarian cancer: the ICON4/AGO-OVAR-2.2 trial. Lancet 361, 2099–2106. 10.1016/s0140-6736(03)13718-x. 12826431

[57] Weaver BA. (2014). How Taxol/paclitaxel kills cancer cells. Mol. Biol. Cell 25, 2677–2681. 10.1091/mbc.E14-04-0916. PMC4161504 25213191

[58] Vemu A, Atherton J, Spector JO, Moores CA, and Roll-Mecak A. (2017). Tubulin isoform composition tunes microtubule dynamics. Mol. Biol. Cell 28, 3564–3572. 10.1091/mbc.E17-02-0124. PMC5706985 29021343

[59] Vemu A, Atherton J, Spector JO, Szyk A, Moores CA, and Roll-Mecak A. (2016). Structure and Dynamics of Single-isoform Recombinant Neuronal Human Tubulin. J. Biol. Chem. 291, 12907–12915. 10.1074/jbc.C116.731133. PMC4933209 27129203

[60] Pamula MC, Ti SC, and Kapoor TM. (2016). The structured core of human beta tubulin confers isotype-specific polymerization properties. J. Cell Biol. 213, 425–433. 10.1083/jcb.201603050. PMC4878094 27185835

[61] Diao L, Liu MY, Song YL, Zhang X, Liang X, and Bao L. (2022). alpha1A and alpha1C form microtubules to display distinct properties mainly mediated by their C-terminal tails. J. Mol. Cell Biol. 13, 864–875. 10.1093/jmcb/mjab062. PMC8800519 34609491

[62] Hirst WG, Biswas A, Mahalingan KK, and Reber S. (2020). Differences in Intrinsic Tubulin Dynamic Properties Contribute to Spindle Length Control in Xenopus Species. Curr. Biol. 30, 2184–2190.e5. 10.1016/j.cub.2020.03.067. 32386526

[63] Panda D, Miller HP, Banerjee A, Luduena RF, and Wilson L. (1994). Microtubule dynamics in vitro are regulated by the tubulin isotype composition. Proc. Natl. Acad. Sci. USA 91, 11358–11362. 10.1073/pnas.91.24.11358. PMC45230 7972064

[64] D’Aguanno S, D’Alessandro A, Pieroni L, Roveri A, Zaccarin M, Marzano V, De Canio M, Bernardini S, Federici G, and Urbani A. (2011). New insights into neuroblastoma cisplatin resistance: a comparative proteomic and meta-mining investigation. J. Proteome Res. 10, 416–428. 10.1021/pr100457n. 21128686

[65] Gan PP, McCarroll JA, Byrne FL, Garner J, and Kavallaris M. (2011). Specific beta-tubulin isotypes can functionally enhance or diminish epothilone B sensitivity in non-small cell lung cancer cells. PLoS One 6, e21717. 10.1371/journal.pone.0021717. PMC3126859 21738778

[66] Huzil JT, Chen K, Kurgan L, and Tuszynski JA. (2007). The roles of beta-tubulin mutations and isotype expression in acquired drug resistance. Cancer Inform. 3, 159–181. PMC2675838 19455242

[67] Widlund PO, Podolski M, Reber S, Alper J, Storch M, Hyman AA, Howard J, and Drechsel DN. (2012). One-step purification of assembly-competent tubulin from diverse eukaryotic sources. Mol. Biol. Cell 23, 4393–4401. 10.1091/mbc.E12-06-0444. PMC3496613 22993214

[68] Vemu A, Garnham CP, Lee DY, and Roll-Mecak A. (2014). Generation of differentially modified microtubules using in vitro enzymatic approaches. Methods Enzymol. 540, 149–166. 10.1016/B978-0-12-397924-7.00009-1. PMC7416453 24630106

[69] Gan PP, Pasquier E, and Kavallaris M. (2007). Class III beta-tubulin mediates sensitivity to chemotherapeutic drugs in non small cell lung cancer. Cancer Res. 67, 9356–9363. 10.1158/0008-5472.CAN-07-0509. 17909044

[70] McCarroll JA, Gan PP, Liu M, and Kavallaris M. (2010). betaIII-tubulin is a multifunctional protein involved in drug sensitivity and tumorigenesis in non-small cell lung cancer. Cancer Res. 70, 4995–5003. 10.1158/0008-5472.CAN-09-4487. 20501838

[71] Ting S, Mairinger FD, Hager T, Welter S, Eberhardt WE, Wohls-chlaeger J, Schmid KW, and Christoph DC. (2013). ERCC1, MLH1, MSH2, MSH6, and betaIII-tubulin: resistance proteins associated with response and outcome to platinum-based chemotherapy in malignant pleural mesothelioma. Clin. Lung Cancer 14, 558–567.e553. 10.1016/j.cllc.2013.04.013. 23810210

[72] Carman CV, Som T, Kim CM, and Benovic JL. (1998). Binding and phosphorylation of tubulin by G protein-coupled receptor kinases. J. Biol. Chem. 273, 20308–20316. 10.1074/jbc.273.32.20308. 9685381

[73] Díaz-Nido J, Serrano L, López-Otín C, Vandekerckhove J, and Avila J. (1990). Phosphorylation of a neuronal-specific beta-tubulin isotype. J. Biol. Chem. 265, 13949–13954. 2199448

[74] Khan IA, and Ludueña RF. (1996). Phosphorylation of beta III-tubulin. Biochemistry 35, 3704–3711. 10.1021/bi951247p. 8619990

[75] Chen J, and Roll-Mecak A. (2023). Glutamylation is a negative regulator of microtubule growth. Mol. Biol. Cell 34, ar70. 10.1091/mbc.E23-01-0030. PMC10295482 37074962

[76] Genova M, Grycova L, Puttrich V, Magiera MM, Lansky Z, Janke C, and Braun M. (2023). Tubulin polyglutamylation differentially regulates microtubule-interacting proteins. EMBO J 42, e112101. 10.15252/embj.2022112101. PMC9975938 36636822

[77] Szczesna E, Zehr EA, Cummings SW, Szyk A, Mahalingan KK, Li Y, and Roll-Mecak A. (2022). Combinatorial and antagonistic effects of tubulin glutamylation and glycylation on katanin microtubule severing. Dev. Cell 57, 2497–2513.e6. 10.1016/j.devcel.2022.10.003. PMC9665884 36347241

[78] Valenstein ML, and Roll-Mecak A. (2016). Graded Control of Microtubule Severing by Tubulin Glutamylation. Cell 164, 911–921. 10.1016/j.cell.2016.01.019. PMC6459029 26875866

[79] Torrino S, Grasset EM, Audebert S, Belhadj I, Lacoux C, Haynes M, Pisano S, Abélanet S, Brau F, Chan SY, (2021). Mechano-induced cell metabolism promotes microtubule glutamylation to force metastasis. Cell Metab. 33, 1342–1357.e10. 10.1016/j.cmet.2021.05.009. 34102109

[80] Fu MM, McAlear TS, Nguyen H, Oses-Prieto JA, Valenzuela A, Shi RD, Perrino JJ, Huang TT, Burlingame AL, Bechstedt S, and Barres BA. (2019). The Golgi Outpost Protein TPPP Nucleates Microtubules and Is Critical for Myelination. Cell 179, 132–146.e14. 10.1016/j.cell.2019.08.025. PMC7214773 31522887

[81] Vincze O, Tökési N, Oláh J, Hlavanda E, Zotter A, Horváth I, Lehotzky A, Tirián L, Medzihradszky KF, Kovács J, (2006). Tubulin polymerization promoting proteins (TPPPs): members of a new family with distinct structures and functions. Biochemistry 45, 13818–13826. 10.1021/bi061305e. 17105200

[82] Lánczky A, and Győrffy B. (2021). Web-Based Survival Analysis Tool Tailored for Medical Research (KMplot): Development and Implementation. J. Med. Internet Res. 23, e27633. 10.2196/27633. PMC8367126 34309564

[83] Gyorffy B, Lánczky A, and Szállási Z. (2012). Implementing an online tool for genome-wide validation of survival-associated biomarkers in ovarian-cancer using microarray data from 1287 patients. Endocr. Relat. Cancer 19, 197–208. 10.1530/ERC-11-0329. 22277193

[84] Zhang K, Erkan EP, Jamalzadeh S, Dai J, Andersson N, Kaipio K, Lamminen T, Mansuri N, Huhtinen K, Carpen O, (2022). Longitudinal single-cell RNA-seq analysis reveals stress-promoted chemoresistance in metastatic ovarian cancer. Sci. Adv. 8, eabm1831. 10.1126/sciadv.abm1831. PMC8865800 35196078

[85] Omura G, Blessing JA, Ehrlich CE, Miller A, Yordan E, Creasman WT, and Homesley HD. (1986). A randomized trial of cyclophosphamide and doxorubicin with or without cisplatin in advanced ovarian carcinoma. A Gynecologic Oncology Group Study. Cancer 57, 1725–1730. 10.1002/1097-0142(19860501)57:9<1725::aid-cncr2820570903>3.0.co;2-j. 3513943

[86] Omura GA, Brady MF, Homesley HD, Yordan E, Major FJ, Buchsbaum HJ, and Park RC. (1991). Long-term follow-up and prognostic factor analysis in advanced ovarian carcinoma: the Gynecologic Oncology Group experience. J. Clin. Oncol. 9, 1138–1150. 10.1200/JCO.1991.9.7.1138. 1904477

[87] Tulub AA, and Stefanov VE. (2001). Cisplatin stops tubulin assembly into microtubules. A new insight into the mechanism of antitumor activity of platinum complexes. Int. J. Biol. Macromol. 28, 191–198. 10.1016/s0141-8130(00)00159-8. 11251225

[88] Peyrot V, Briand C, Momburg R, and Sari JC. (1986). In vitro mechanism study of microtubule assembly inhibition by cis-dichlorodiammine-platinum(II). Biochem. Pharmacol. 35, 371–375. 10.1016/0006-2952(86)90207-8. 3947376

[89] Boekelheide K, Arcila ME, and Eveleth J. (1992). cis-diamminedichloroplatinum (II) (cisplatin) alters microtubule assembly dynamics. Toxicol. Appl. Pharmacol. 116, 146–151. 10.1016/0041-008x(92)90156-m. 1529448

[90] Békés M, Langley DR, and Crews CM. (2022). PROTAC targeted protein degraders: the past is prologue. Nat. Rev. Drug Discov. 21, 181–200. 10.1038/s41573-021-00371-6. PMC8765495 35042991

[91] Shih AJ, Menzin A, Whyte J, Lovecchio J, Liew A, Khalili H, Bhuiya T, Gregersen PK, and Lee AT. (2018). Identification of grade and origin specific cell populations in serous epithelial ovarian cancer by single cell RNA-seq. PLoS One 13, e0206785. 10.1371/journal.pone.0206785. PMC6211742 30383866

[92] Jekunen AP, Christen RD, Shalinsky DR, and Howell SB. (1994). Synergistic interaction between cisplatin and taxol in human ovarian carcinoma cells in vitro. Br. J. Cancer 69, 299–306. 10.1038/bjc.1994.55. PMC1968703 7905279

[93] Mohith A, Photiou A, and Retsas S. (1996). The combination of paclitaxel with cisplatin exhibits antagonism in vitro against human melanoma. Anticancer. Drugs 7, 493–498. 10.1097/00001813-199606000-00016. 8826619

[94] Vanhoefer U, Harstrick A, Wilke H, Schleucher N, Walles H, Schröder J, and Seeber S. (1995). Schedule-dependent antagonism of paclitaxel and cisplatin in human gastric and ovarian carcinoma cell lines in vitro. Eur. J. Cancer 31A, 92–97. 10.1016/0959-8049(94)00440-g. 7695986

[95] Muggia FM. (2004). Relevance of chemotherapy dose and schedule to outcomes in ovarian cancer. Semin. Oncol. 31, 19–24. 10.1053/j.seminoncol.2004.11.024. 15726535

[96] Siegal T, and Haim N. (1990). Cisplatin-induced peripheral neuropathy. Frequent off-therapy deterioration, demyelinating syndromes, and muscle cramps. Cancer 66, 1117–1123. 10.1002/1097-0142(19900915)66:6<1117::aid-cncr2820660607>3.0.co;2-o. 2169332

[97] Roelofs RI, Hrushesky W, Rogin J, and Rosenberg L. (1984). Peripheral sensory neuropathy and cisplatin chemotherapy. Neurology 34, 934–938. 10.1212/wnl.34.7.934. 6330613

[98] van der Hoop RG, van der Burg ME, ten Bokkel Huinink WW, van Houwelingen C, and Neijt JP. (1990). Incidence of neuropathy in 395 patients with ovarian cancer treated with or without cisplatin. Cancer 66, 1697–1702. 10.1002/1097-0142(19901015)66:8<1697::aid-cncr2820660808>3.0.co;2-g. 2119878

[99] van Zeijl LG, Conijn EA, Rodenburg M, Tange RA, and Brocaar MP. (1984). Analysis of hearing loss due to cis-diamminedichloroplatinum-II. Arch. Otorhinolaryngol. 239, 255–262. 10.1007/BF00464252. 6539589

[100] Paken J, Govender CD, Pillay M, and Sewram V. (2016). Cisplatin-Associated Ototoxicity: A Review for the Health Professional. J. Toxicol. 2016, 1809394. 10.1155/2016/1809394. PMC5223030 28115933

[101] Knight KRG, Kraemer DF, and Neuwelt EA. (2005). Ototoxicity in children receiving platinum chemotherapy: underestimating a commonly occurring toxicity that may influence academic and social development. J. Clin. Oncol. 23, 8588–8596. 10.1200/JCO.2004.00.5355. 16314621

[102] Patel RP, Kuhn S, Yin D, Hotz JM, Maher FA, Robey RW, Gottesman MM, and Horibata S. (2021). Cross-resistance of cisplatin selected cells to anti-microtubule agents: Role of general survival mechanisms. Transl. Oncol. 14, 100917. 10.1016/j.tranon.2020.100917. PMC7586247 33129114

[103] Chertkova AO, Mastop M, Postma M, van Bommel N, van der Niet S, Batenburg KL, Joosen L, Gadella TWJ, Okada Y, and Goedhart J. (2020). Robust and Bright Genetically Encoded Fluorescent Markers for Highlighting Structures and Compartments in Mammalian Cells. bioRxiv. 10.1101/160374.

[104] Schneider CA, Rasband WS, and Eliceiri KW. (2012). NIH Image to ImageJ: 25 years of image analysis. Nat. Methods 9, 671–675. 10.1038/nmeth.2089. PMC5554542 22930834

[105] Schindelin J, Arganda-Carreras I, Frise E, Kaynig V, Longair M, Pietzsch T, Preibisch S, Rueden C, Saalfeld S, Schmid B, (2012). Fiji: an open-source platform for biological-image analysis. Nat. Methods 9, 676–682. 10.1038/nmeth.2019. PMC3855844 22743772

[106] Applegate KT, Besson S, Matov A, Bagonis MH, Jaqaman K, and Danuser G. (2011). plusTipTracker: Quantitative image analysis software for the measurement of microtubule dynamics. J. Struct. Biol. 176, 168–184. 10.1016/j.jsb.2011.07.009. PMC3298692 21821130

[107] Jaqaman K, Loerke D, Mettlen M, Kuwata H, Grinstein S, Schmid SL, and Danuser G. (2008). Robust single-particle tracking in live-cell time-lapse sequences. Nat. Methods 5, 695–702. 10.1038/nmeth.1237. PMC2747604 18641657

[108] Chen J, Kholina E, Szyk A, Fedorov VA, Kovalenko I, Gudimchuk N, and Roll-Mecak A. (2021). alpha-tubulin tail modifications regulate microtubule stability through selective effector recruitment, not changes in intrinsic polymer dynamics. Dev. Cell 56, 2016–2028.e2014. 10.1016/j.devcel.2021.05.005. PMC8476856 34022132

[109] Harrell J FE (2026). rms: Regression Modeling Strategies, R package version 8.1-0. https://CRAN.R-project.org/package=rms.

[110] Warnes G, Bolker B, Bonebakker L, Gentleman R, Huber W, Liaw A, Lumley T, Maechler M, Magnusson A, Moeller S, (2025). gplots: Various R Programming Tools for Plotting Data, R package version 3.3.0. https://github.com/talgalili/gplots.

[111] Lehotzky A, Oláh J, Szunyogh S, Szabó A, Berki T, and Ovádi J. (2015). Zinc-induced structural changes of the disordered tppp/p25 inhibits its degradation by the proteasome. Biochim. Biophys. Acta 1852, 83–91. 10.1016/j.bbadis.2014.10.015. 25445539

[112] Horibata S, Vo TV, Subramanian V, Thompson PR, and Coonrod SA. (2015). Utilization of the Soft Agar Colony Formation Assay to Identify Inhibitors of Tumorigenicity in Breast Cancer Cells. J. Vis. Exp. e52727. 10.3791/52727. PMC4542786 26067809

[113] Castoldi M, and Popov AV. (2003). Purification of brain tubulin through two cycles of polymerization-depolymerization in a high-molarity buffer. Protein Expr. Purif. 32, 83–88. 10.1016/S1046-5928(03)00218-3. 14680943

[114] Gell C, Bormuth V, Brouhard GJ, Cohen DN, Diez S, Friel CT, Helenius J, Nitzsche B, Petzold H, Ribbe J, (2010). Microtubule dynamics reconstituted in vitro and imaged by single-molecule fluorescence microscopy. Methods Cell Biol. 95, 221–245. 10.1016/S0091-679X(10)95013-9. 20466138

[115] Toso RJ, Jordan MA, Farrell KW, Matsumoto B, and Wilson L. (1993). Kinetic stabilization of microtubule dynamic instability in vitro by vinblastine. Biochemistry 32, 1285–1293. 10.1021/bi00056a013. 8448138

[116] Ziółkowska NE, and Roll-Mecak A. (2013). In vitro microtubule severing assays. Methods Mol. Biol. 1046, 323–334. 10.1007/978-1-62703-538-5_19. 23868597

[117] Bieling P, Laan L, Schek H, Munteanu EL, Sandblad L, Dogterom M, Brunner D, and Surrey T. (2007). Reconstitution of a microtubule plus-end tracking system in vitro. Nature 450, 1100–1105. 10.1038/nature06386. 18059460

