# Supplementary material for: Cisplatin resistance in an ovarian cancer model is mediated by microtubule dynamics regulator TPPP3 in synergy with tubulin code rewiring
Source: Cell Rep. Author manuscript; Available in PMC 2026 Sep 26. (PMC13615893; doi:10.1016/j.celrep.2026.117414)
Supplement: Supplementary file 1 [file NIHMS2190712-supplement-1.pdf]

Cell Reports, Volume *45*

Supplemental information

Cisplatin resistance in an ovarian cancer model is mediated by microtubule dynamics regulator TPPP3 in synergy with tubulin code rewiring

Sachi Horibata, Kishore K. Mahalingan, Ruchi Patel, Yu Fan, Jordan M. Hotz, Adriana Ponton-Almodovar, Keith MacRenaris, Yan Li, Daoud Meerzaman, Michael M. Gottesman, and Antonina Roll-Mecak

Tubulin Isoform	Region with Glutamylation site	Number of glutamates	Average Intensity (n=4)			CP-R/CP-S	CP-RS/CP-S
			CP-S	CP-R	CP-RS		
βI	435-444	1	204020.4	230887.6	115724.9	0.99	0.57
βIIA/βIIB	431-445	1	353517.4	7343529.4	1335315.6	20.67	3.76
βI	427-444	1	13747888.3	13844856.5	4591399.9	1.09	0.35
βIIA/βIIB	427-445	1	62571.2	422431.3	ND	7.91	<0.01
βIVA	427-444	2	64128.3	162366.7	ND	1.64	<0.01
βIVB	427-444	1	3497296.4	10536533.6	1157655.0	2.85	0.31
βI	427-444	2	1824422.0	725505.8	416721.2	0.42	0.24
β6	427-446	1	721922.4	4544794.7	652762.8	6.60	0.94
α3D	431-450	1	509401.0	ND	1044076.2	<0.01	2.07
α1A/α1B	431-451	1	22120.3	93373.9	13557.4	3.95	0.59
βIII	427-450	1	585723.3	141628608.9	27029781.1	241.80	46.19
α1C	424-449	1	837823.6	779682.2	607407.6	0.92	0.70
α1A/α1B	424-451	1	10612963.5	53660034.2	49526609.9	5.37	4.58
α1A/α1B	424-451	2	893999.4	5171665.7	1007571.0	5.66	1.18
βI	417-444	1	9194986.0	3177270.2	ND	0.32	<0.01
βIVB	417-445	1	696204.4	1229293.0	ND	1.91	<0.01
βIII	417-450	1	160854.7	18351167.7	519403.4	114.09	3.23
βIIA/βIIB	123-154	1	ND	11164250.6	20875759.2	>100	>100

ND - None detected

Table S1: Label free quantification using tandem mass-spectrometry and comparison of α- and β-tubulin glutamylation in purified tubulin from the three ovarian cancer cell lines. (related to Figure 4)

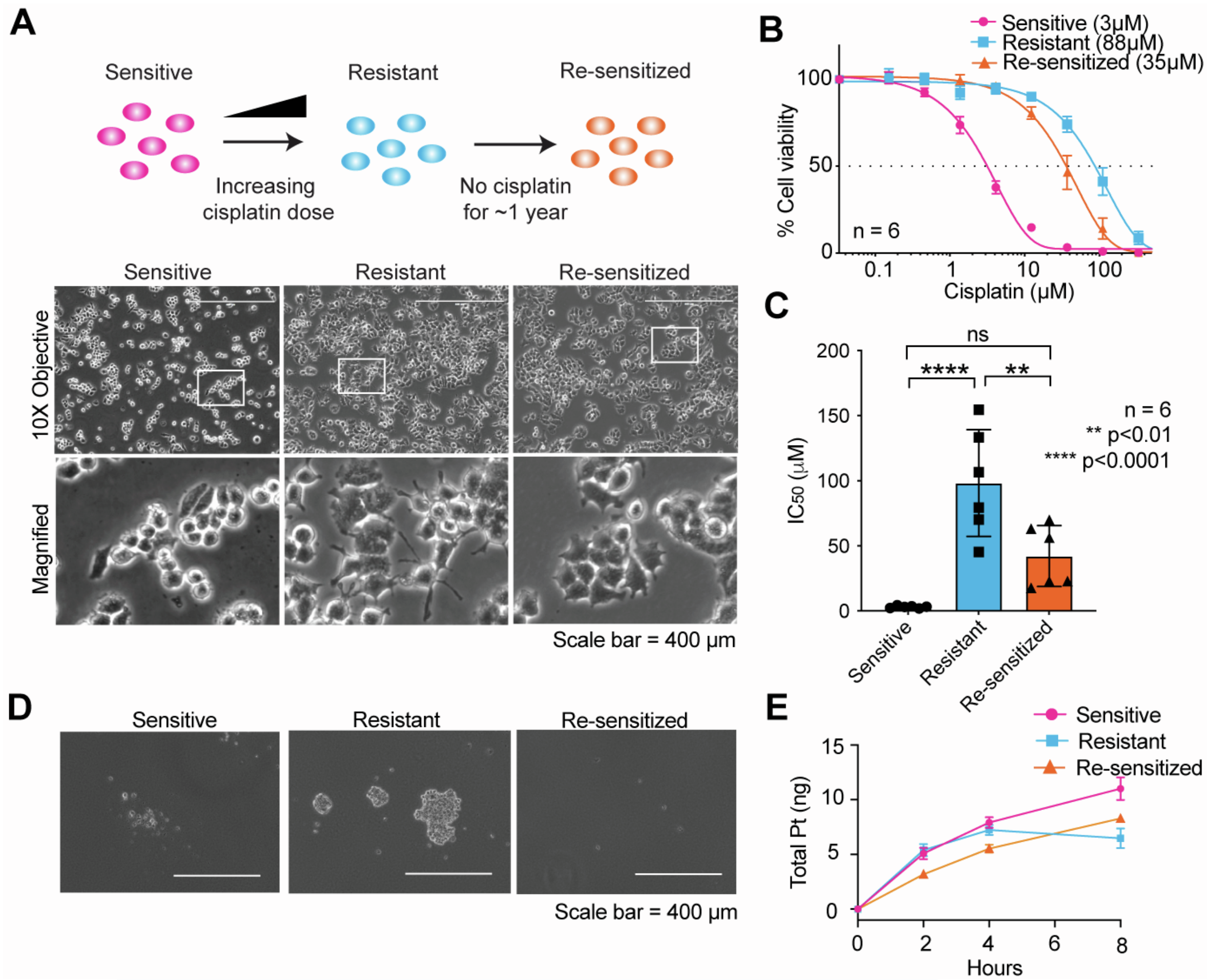

Figure S1. Validation of cisplatin resistant lines. (related to Figure 1)

(A) Schematic illustration of study design and accompanying wide-field images of sensitive, resistant, and partially re-sensitized ovarian cancer cells illustrating the cellular protrusions found in resistant cells but not in sensitive or partially re-sensitized cells. Scale bar = 400 μm.

(B-C) Cytotoxicity assay showing the cell viability curve (B) and the IC_{50} (C) of sensitive, resistant, and partially re-sensitized after cisplatin treatment. Statistical significance for IC_{50} calculated using one-way ANOVA. n values indicate 6 independent experiments. ns, not significant, **, $p < 0.001$ and ****, $p < 0.0001$ as determined by one-way ANOVA.

(D) Soft agar colony formation of sensitive, resistant, and re-sensitized ovarian cancer cells in the presence of 80 μM cisplatin shows that resistant cells are the only ones that can form spheroid colonies in the presence of cisplatin. Scale bar = 400 μm.

(E) Inductively coupled plasma mass spectrometry (ICP-MS) shows total platinum amount that enters cisplatin-sensitive, -resistant, and -re-sensitized cells upon treatment with 80 μM cisplatin at time 0, 2, 4, and 8 hours. Whole cell lysates were used for this assay; n = 3 independent experiments.

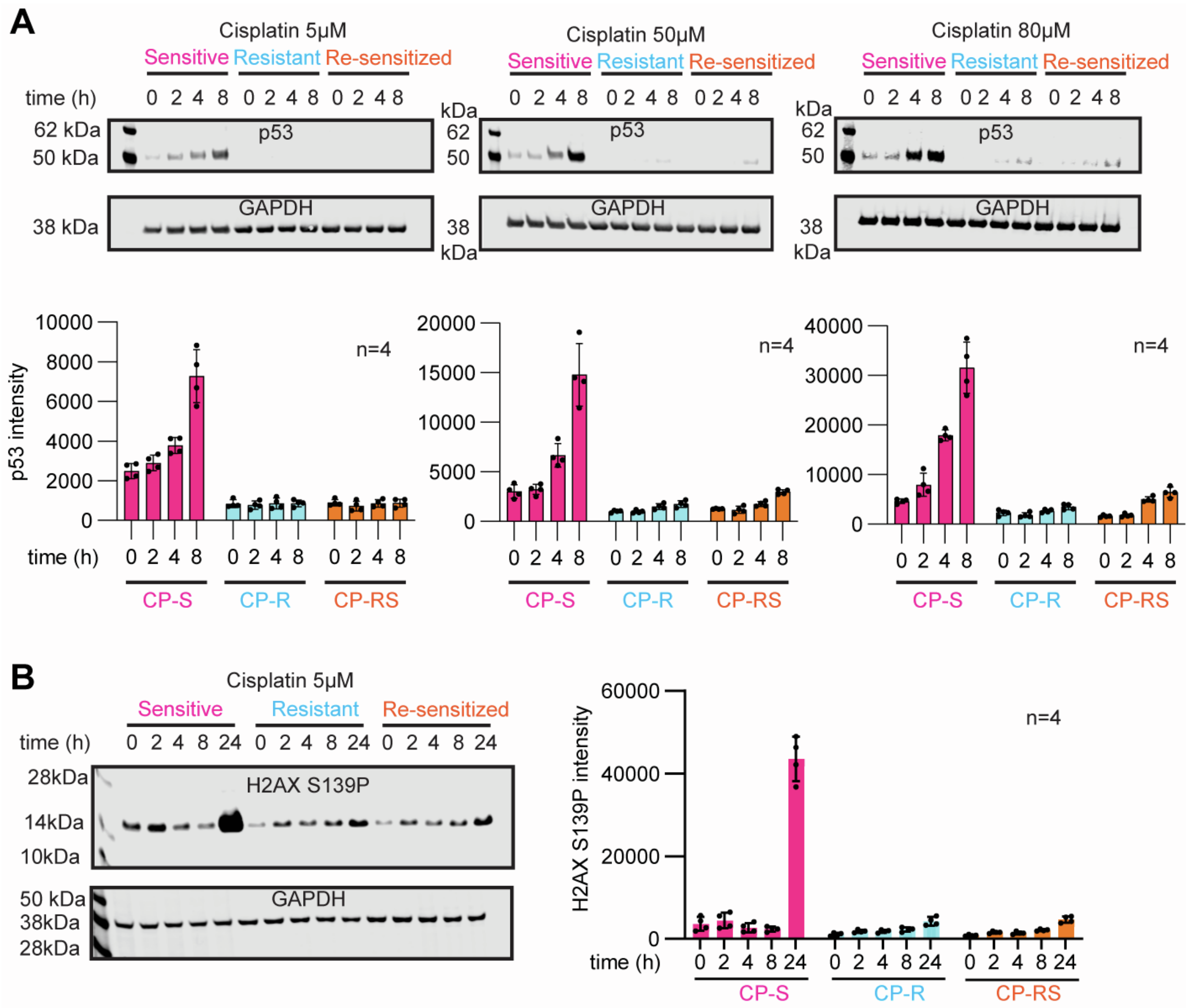

Figure S2. p53 and H2AX S139 phosphorylation levels after cisplatin treatment are similar between cisplatin resistant and re-sensitized cells. (related to Figure 1)

(A) Western blot images and quantification of p53 expression at different timepoints after treatment with 5, 50, and 80 μM cisplatin in sensitive (magenta), resistant (cyan), and re-sensitized (orange) cells.

(B) Western blot images and quantification of H2AX S139 phosphorylation at different time points after treatment with 5 μM of cisplatin in sensitive, resistant, and re-sensitized cells. n = 4 independent experiments.

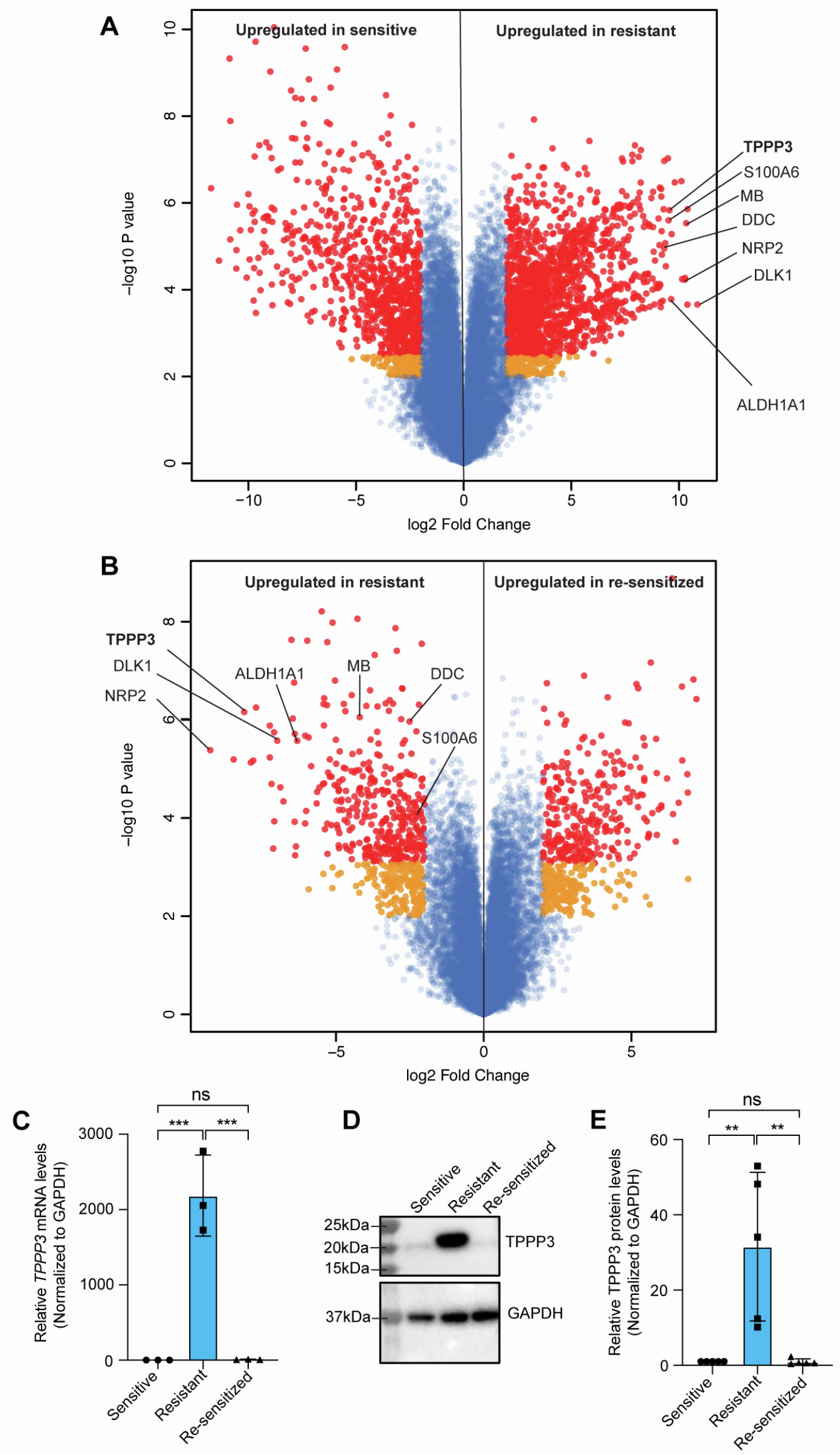

Figure S3. RNA sequencing analysis identifies TPPP3 as a target of chemoresistance. (related to Figure 1)

(A-B) Differential gene expression of RNA sequencing analysis for cisplatin-resistant versus sensitive cells (A) and cisplatin-re-sensitized versus resistant cells (B) displayed as -log10 *p*-value against the log2 fold-change. Genes with a *p*-value < 1e-2 and log2 fold-change > 2 are displayed as orange or red (significant genes) and *p*-value > 1e-2 or log2 fold-change < 2 are displayed as blue (not significant genes). The top seven genes upregulated in resistant cells and downregulated in sensitive and re-sensitized cells are labeled.

(C) qPCR analyses of TPPP3 in sensitive, resistant, and re-sensitized cells with 3 independent experiments validating the upregulation of TPPP3 in resistant cells. GAPDH is used as a reference control. Error bars, S.D. n = 3 independent experiments; ***, p < 0.0005 as determined by one way ANOVA.

(D-E) Western blot images (D) and quantification of TPPP3 protein expression level (E) in sensitive, resistant, and re-sensitized cells from 5 independent experiments. GAPDH is used as a reference control. Error bars, S.D. **, p < 0.005 as determined by one-way ANOVA.

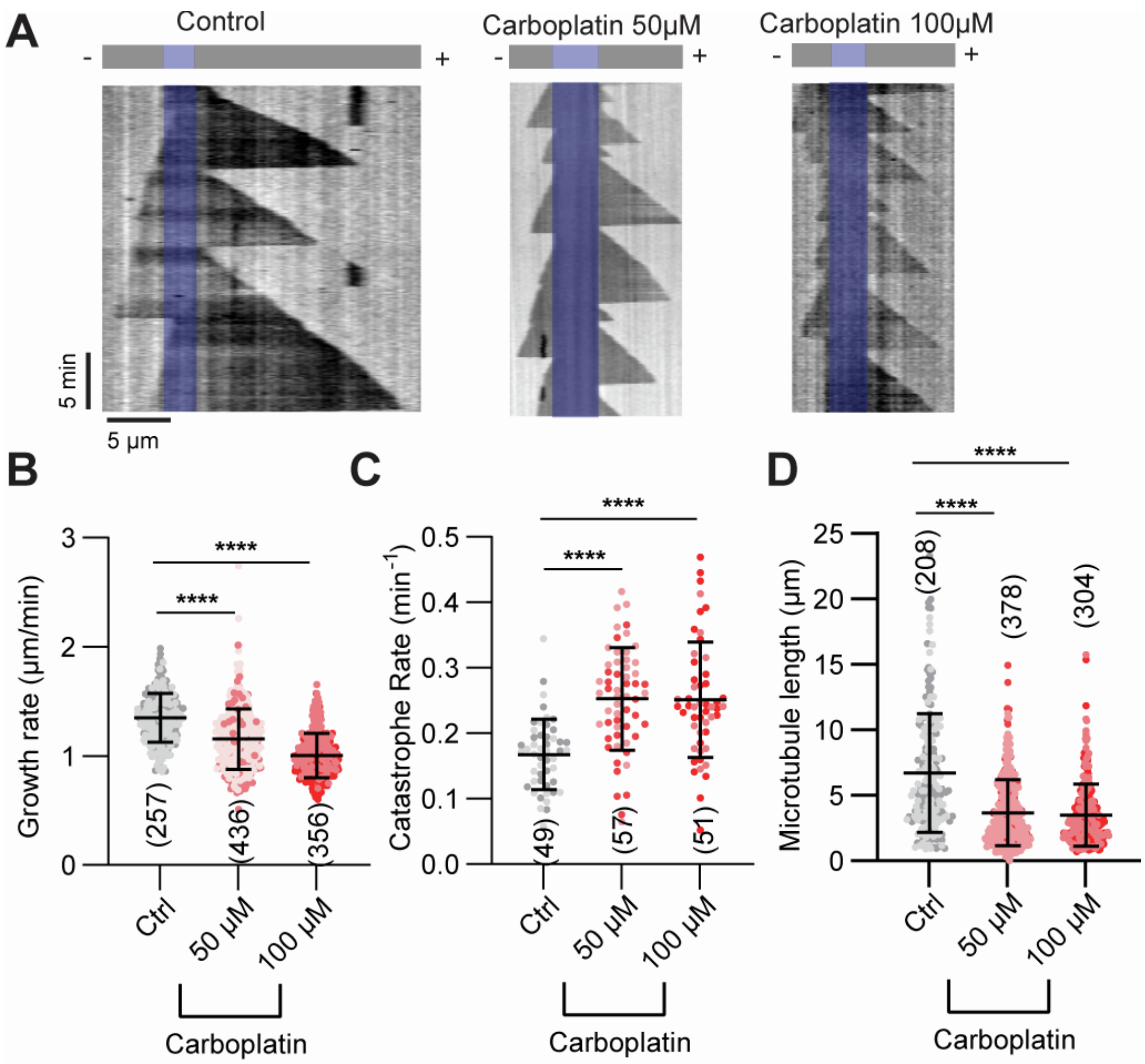

Figure S4. Effects of carboplatin on microtubule dynamics with purified tubulin *in vitro.* (related to Figure 2)

(A) Representative kymographs showing microtubule growth at 10μM brain tubulin in the presence of 50 and 100 μM carboplatin; GMPCPP-stabilized seeds highlighted in blue. Scale bar = 5 μm.

(B-D) Microtubule plus-end growth rates (B), microtubule plus-end catastrophe rates (C) and microtubule lengths (D) at 10μM porcine brain tubulin with different carboplatin concentrations. Error bars, S.D. Microtubule numbers indicated in parentheses throughout from 2 independent experiments. ****, $p < 0.0001$, as determined by one way ANOVA using Tukey's multiple comparison test.

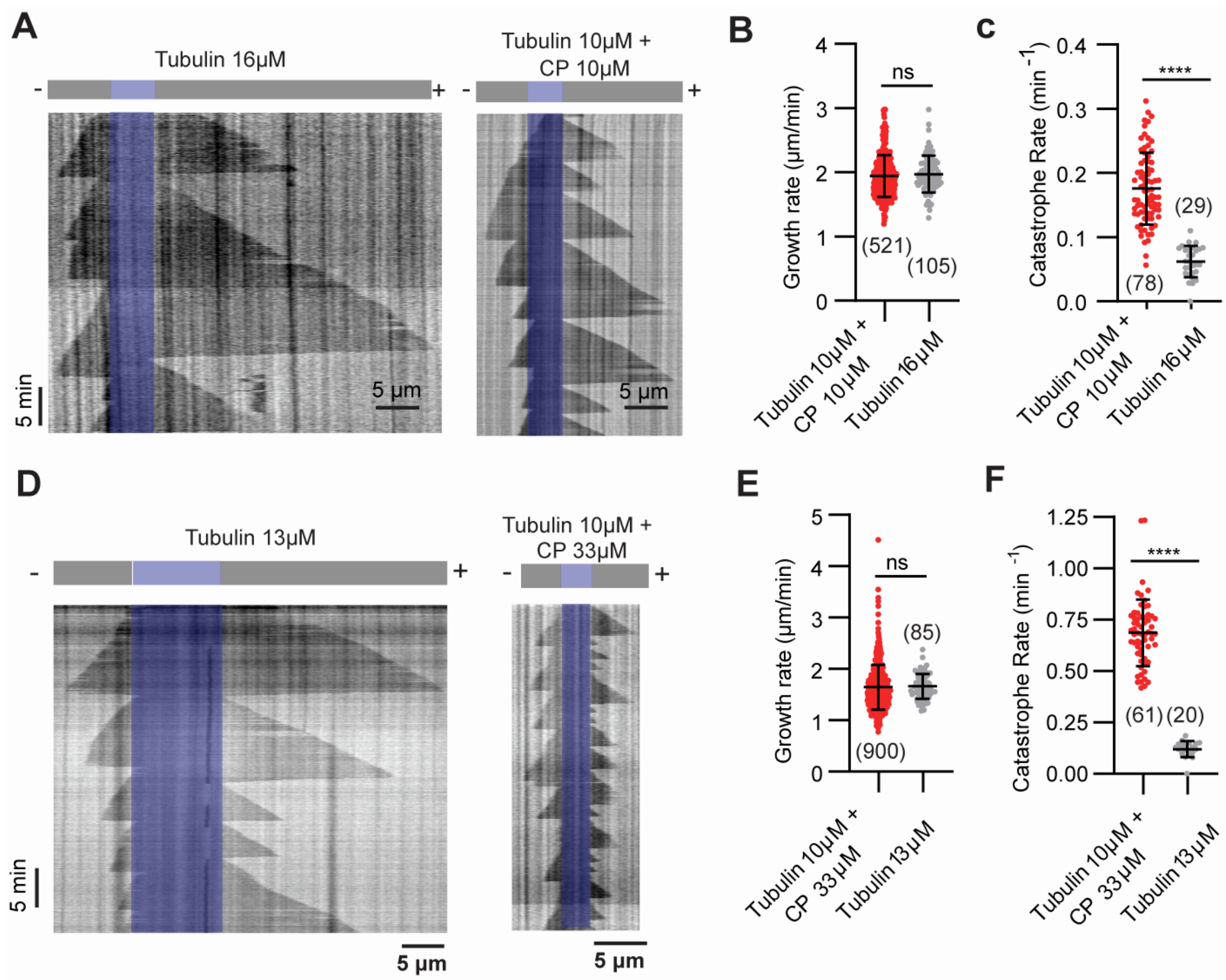

Figure S5. Cisplatin destabilizes microtubule ends and induces catastrophe. (related to Figure 2)

(A) Kymographs showing microtubule growth at 16 μM porcine brain tubulin, and 10 μM porcine brain tubulin in the presence of 10 μM cisplatin. GMPCPP-stabilized seeds are highlighted in blue. Microtubule growth rates under these conditions are matched within 10% S.D. Scale bar = 5 μm.

(B) Scatter plot showing microtubule growth rates at 10μM porcine brain tubulin in the presence of 10 μM cisplatin, and 16 μM porcine brain tubulin without cisplatin. Microtubule growth rates under these conditions are matched within 10% S.D. (1.94 ± 0.32 μm/min and 1.97 ± 0.28 μm/min, respectively).

(C) Catastrophe rates for growth-rate matched microtubules at 10μM porcine brain tubulin in the presence of 10 μM cisplatin and 16 μM porcine brain tubulin.

(D) Kymographs showing microtubule growth at 13 μM porcine brain tubulin, and 10 μM porcine brain tubulin in the presence of 33 μM cisplatin. GMPCPP-stabilized seeds are highlighted in blue. Scale bar = 5 μm.

(E) Scatter plot showing microtubule growth rates of 10μM porcine brain tubulin in the presence of 33 μM cisplatin, and 13 μM porcine brain tubulin without cisplatin. Microtubule growth rates under these conditions are matched within 10% S.D (1.66 ± 0.43 μm/min and 1.65 ± 0.24 μm/min respectively).

(F) Catastrophe rates for growth-rate matched 10μM porcine brain tubulin in the presence of 33 μM cisplatin, and 13 μM porcine brain tubulin without cisplatin. n values (growth events for (B), (E), microtubules for (C), (F)) shown in parentheses throughout from 2 independent experiments on different days. Error bars, S.D. **** $p < 0.0001$, ns, not significant as determined by Mann-Whitney test.

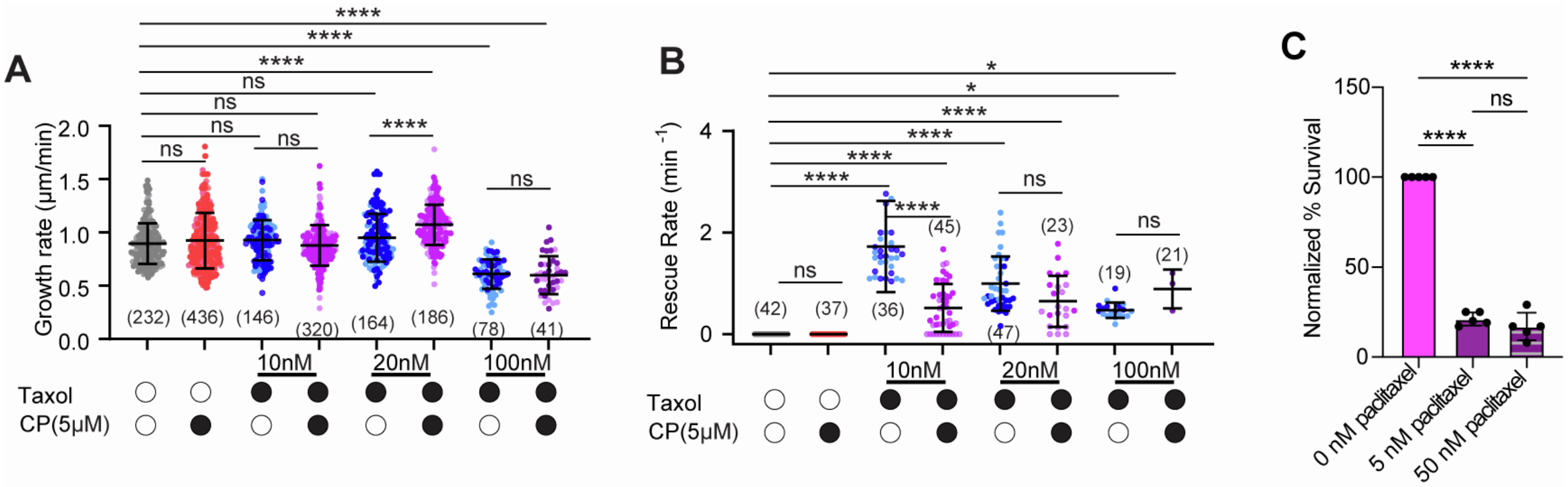

Figure S6. Clinical concentrations of cisplatin and paclitaxel exhibit antagonistic effects on microtubule dynamics *in vitro* (related to Figure 3)

(A-B) microtubule plus-end growth rates (A) and microtubule plus-end rescue rates (B) at 6 μM of porcine brain tubulin in the presence of cisplatin alone, paclitaxel alone, and cisplatin in combination with paclitaxel at different concentrations. Error bars, S.D.; n values, growth events for (A) and microtubules for (B) shown in parentheses throughout from 2, 3, 2, 2, 2, 2, 2 independent experiments on different days for control, 5 μM cisplatin, 10 nM paclitaxel, 10 nM paclitaxel and 5 μM cisplatin, 20 nM paclitaxel, 20 nM paclitaxel and 5 μM cisplatin, 100 nM paclitaxel, 100 nM paclitaxel and 5 μM cisplatin, respectively. ****, $p < 0.0001$, *, $p < 0.05$, and ns, non-significant, as determined by one-way ANOVA using Tukey's multiple comparison test.

(C) Cell viability of sensitive cells treated with 0, 5 or 50 nM paclitaxel; Error bars, S.D. ****, $p < 0.0001$, ns, non-significant as determined by one-way ANOVA.

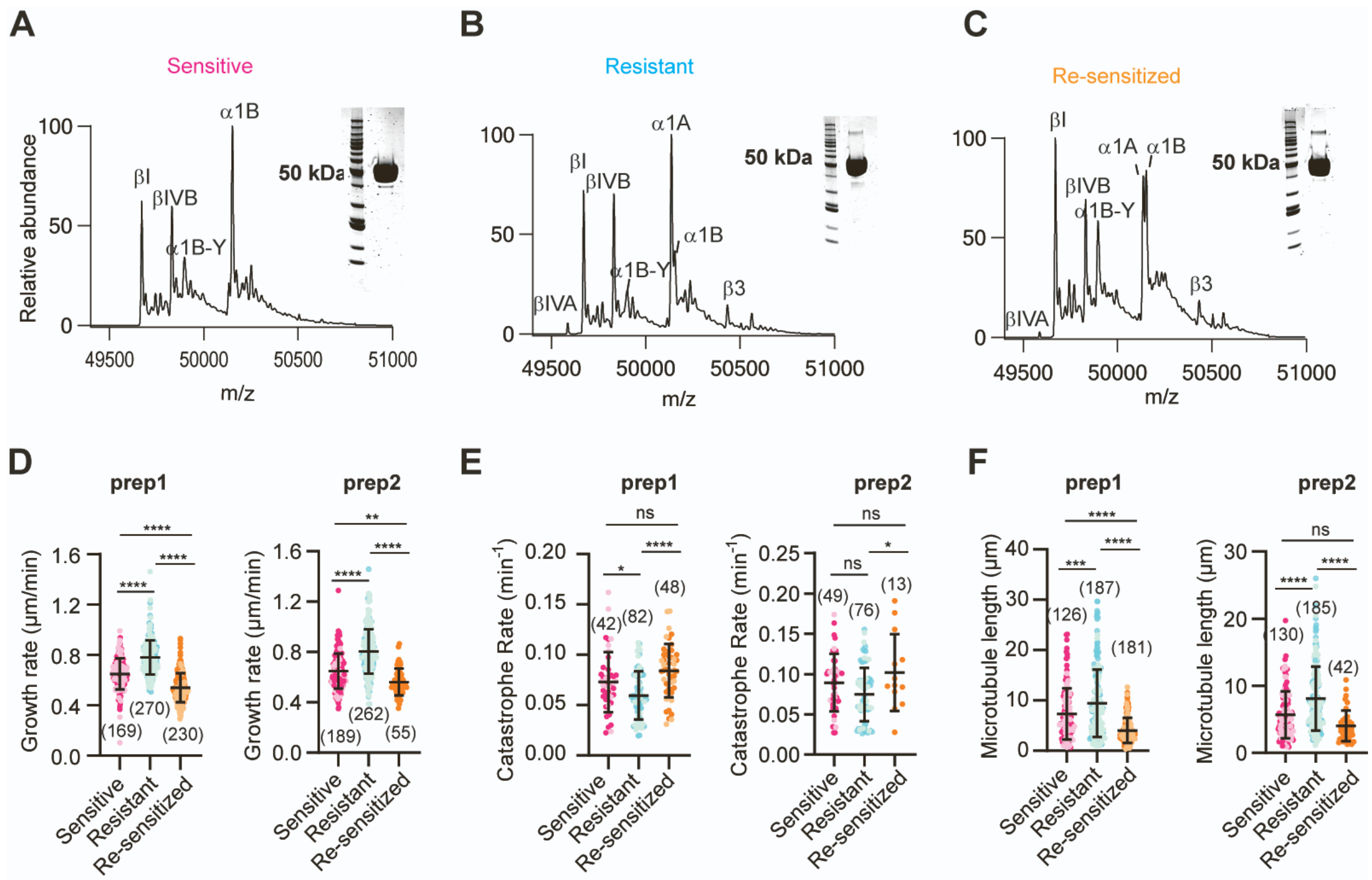

Figure S7. Purity and LC-MS analysis of purified tubulin from ovarian cancer cells. (related to Figure 4)

(A-C) Liquid Chromatography-Mass spectroscopy (LC-MS) spectra and Coomassie stained SDS-polyacrylamide gel (inset) of tubulin purified from cisplatin -sensitive (CP-S) (A) cisplatin-resistant (CP-R) (B), and cisplatin-re-sensitized (CP-RS) cells (C).

(D-F) Microtubule plus-end growth rates (D), microtubule plus-end catastrophe rates (E) and microtubule lengths (F) at 6μM of tubulin purified from sensitive (magenta), resistant (cyan), and re-sensitized (orange) cells from two independent tubulin preparations. Error bars, S.D. n values indicated in parentheses throughout. ****, $p < 0.0001$, **, $p < 0.01$, *, $p < 0.1$, and ns, non-significant, as determined by one-way ANOVA using Tukey's multiple comparison test.

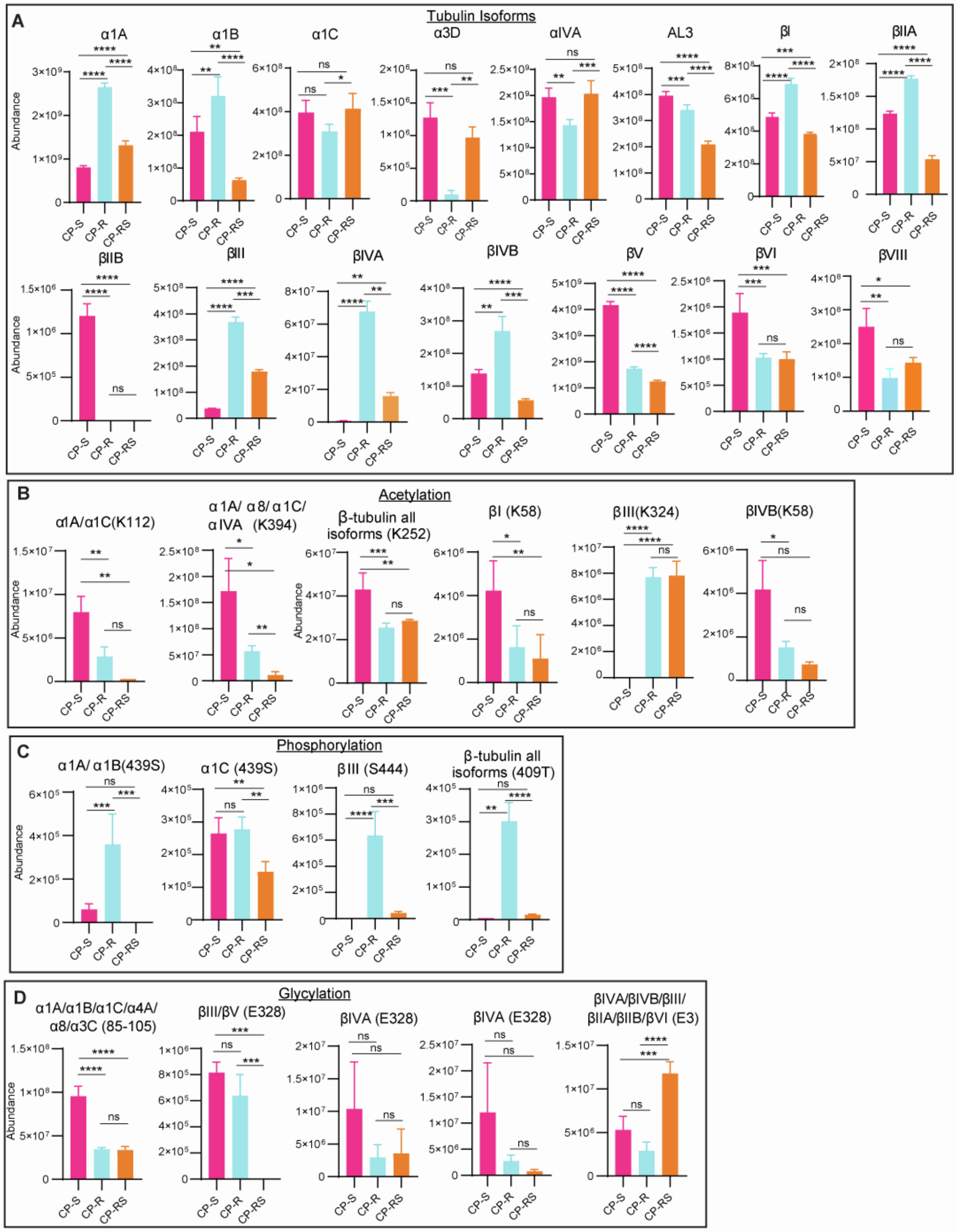

Figure S8. Relative levels of tubulin isoforms and tubulin posttranslational modifications among the three cancer cell lines. (related to Figure 4)

(A) Label free quantification of the abundance of different α- and β-tubulin isoforms present in the CP-Sensitive, CP-resistant, and CP-resensitized cancer cells.

(B-D) Label free quantification of (B) α- and β-tubulin acetylation (C) α- and β-tubulin phosphorylation, and (D) α- and β-tubulin glycylation in the CP-Sensitive, CP-resistant, and CP-re-sensitized cancer cells; n = 5 replicates. ****, $p < 0.0001$, ***, $p < 0.001$, **, $p < 0.01$, *, $p < 0.1$, and ns, non-significant, as determined by one way ANOVA using Tukey's multiple comparison test.

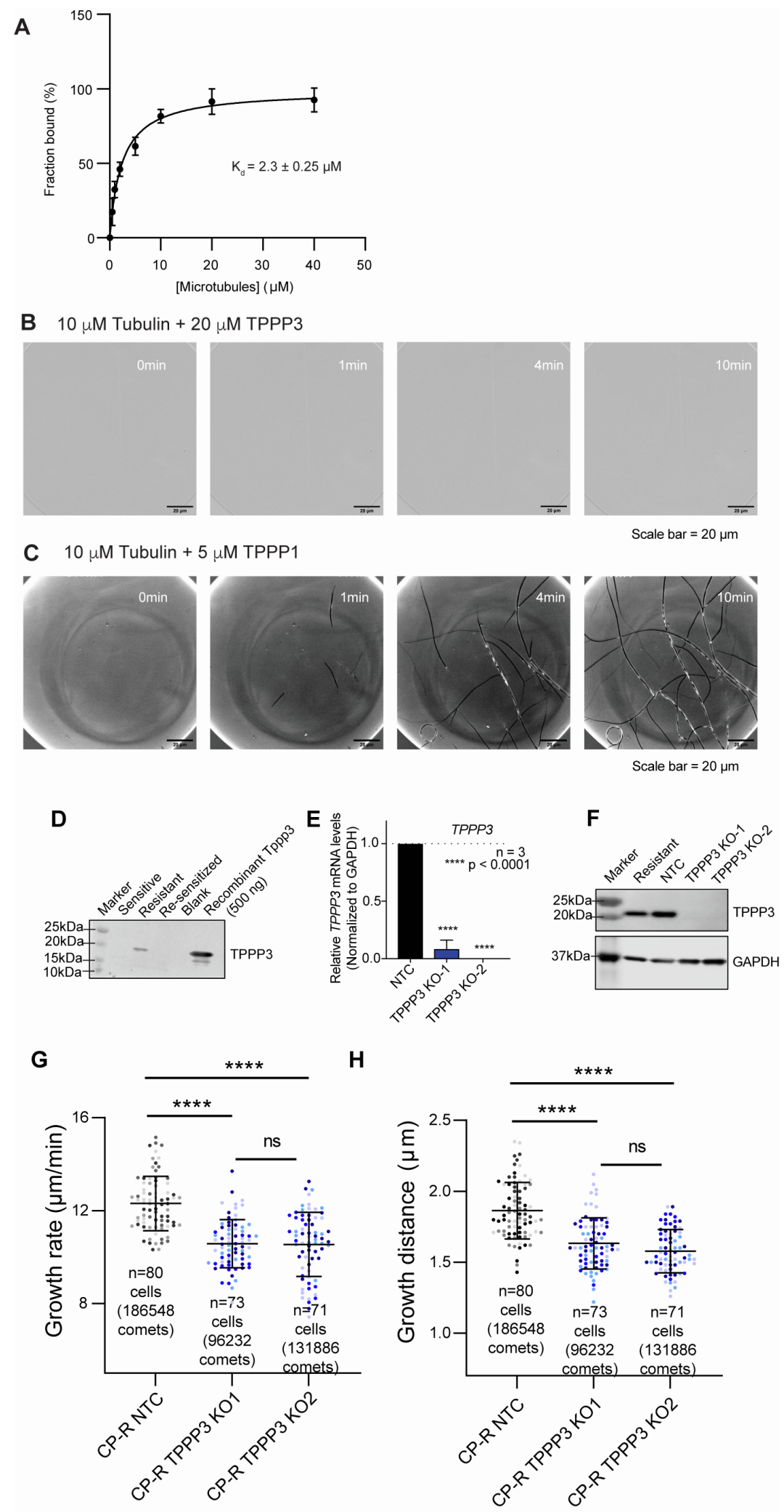

Figure S9. TPPP3 binds microtubules and regulates microtubule dynamics in cells (related to Figure 5)

(A) Graph showing TPPP3 binding to microtubules as quantified from microtubule pelleting assays (STAR Methods); n = 2 independent experiments.

(B-C) Interference reflection microscopy (IRM) images at different time points (t = 0, 1, 4 and 10 min) showing no microtubule nucleation in the presence of 20 μM TPPP3 (B) and robust microtubule nucleation induced by 5 μM TPPP1 (C). Scale bar = 20 μm.

(D) Western blot images of TPPP3 in cisplatin- sensitive, -resistant, -re-sensitized cells and 500ng of recombinant TPPP3. The recombinant protein was used to quantify the TPPP3 levels in cisplatin-resistant cells.

(E) qPCR of CRISPR-knock out of TPPP3 (TPPP3-KO) in resistant cells. qPCR was performed in 3 independent experiments and normalized to GAPDH. **** $p < 0.0001$.

(F) Western blot of CRISPR-knock out of TPPP3 (TPPP3-KO) in resistant cells. GAPDH was used as a reference.

(G-H) Microtubule growth rates (A) and growth distances (B) for cisplatin resistant non-targeting control (NTC) and TPPP3-KO cells; n = 80, 73 and 71 cells for the NTC, TPPP3-KO1 and TPPP3-KO2, respectively from three independent experiments. Data collected from independent experiments on different days are shown in different shades. The number of cells and EB3-mScarlet comets analyzed are indicated on the graph; ns, non-significant and **** denotes $p < 0.0001$, as determined by Mann-Whitney test.

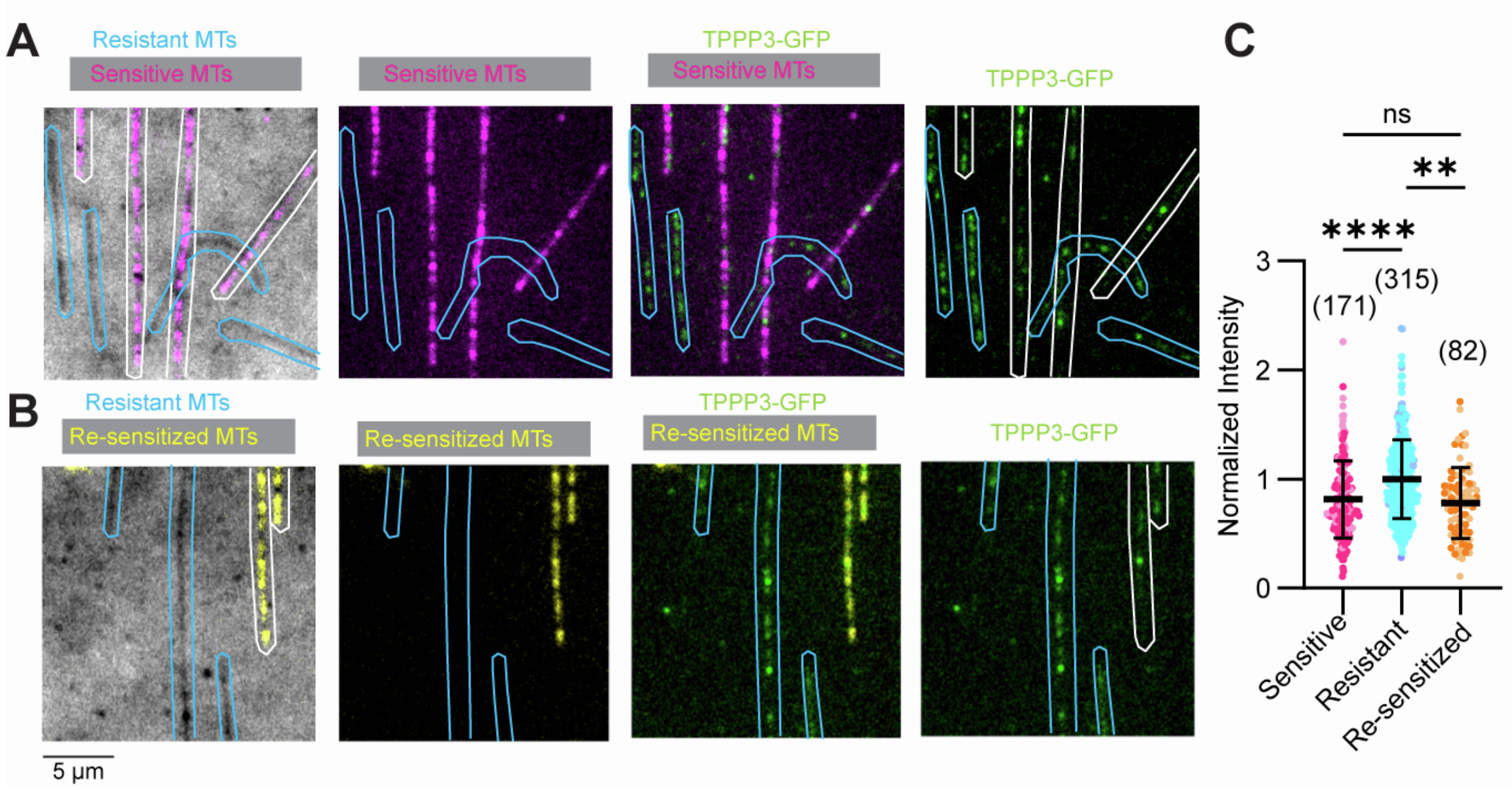

Figure S10. Differential binding of TPPP3 to microtubules from cisplatin-resistant, sensitized and re-sensitized cells. (related to Figure 5)

(A) Images showing TPPP3-GFP (green) recruitment to microtubules assembled from tubulin isolated from cisplatin-sensitive and cisplatin-resistant cells. Cisplatin-sensitive microtubules labeled with HiLyte647 (Methods), colored magenta and outlined in white, cisplatin-resistant microtubules outlined in cyan. Scale bar = 5 μm.

(B) Images showing TPPP3-GFP (green) recruitment to microtubules assembled from tubulin isolated from re-sensitized and cisplatin-resistant cells. Re-sensitized microtubules labeled with HiLyte647 (Methods), colored yellow and outlined in white, cisplatin-resistant microtubules outlined in cyan. Scale bar = 5 μm.

(C) Normalized measurement of TPPP3-GFP recruitment to microtubules assembled from tubulin purified from cisplatin-sensitive, resistant, and re-sensitized cells; n = 171, 315 and 82 microtubules for cisplatin-sensitive, resistant, and re-sensitized, respectively; 2 independent experiments on different days. Error bars, S.D.; ****, $p < 0.0001$, **, $p < 0.01$, ns, non-significant as determined by one way ANOVA using Tukey's multiple comparison test.

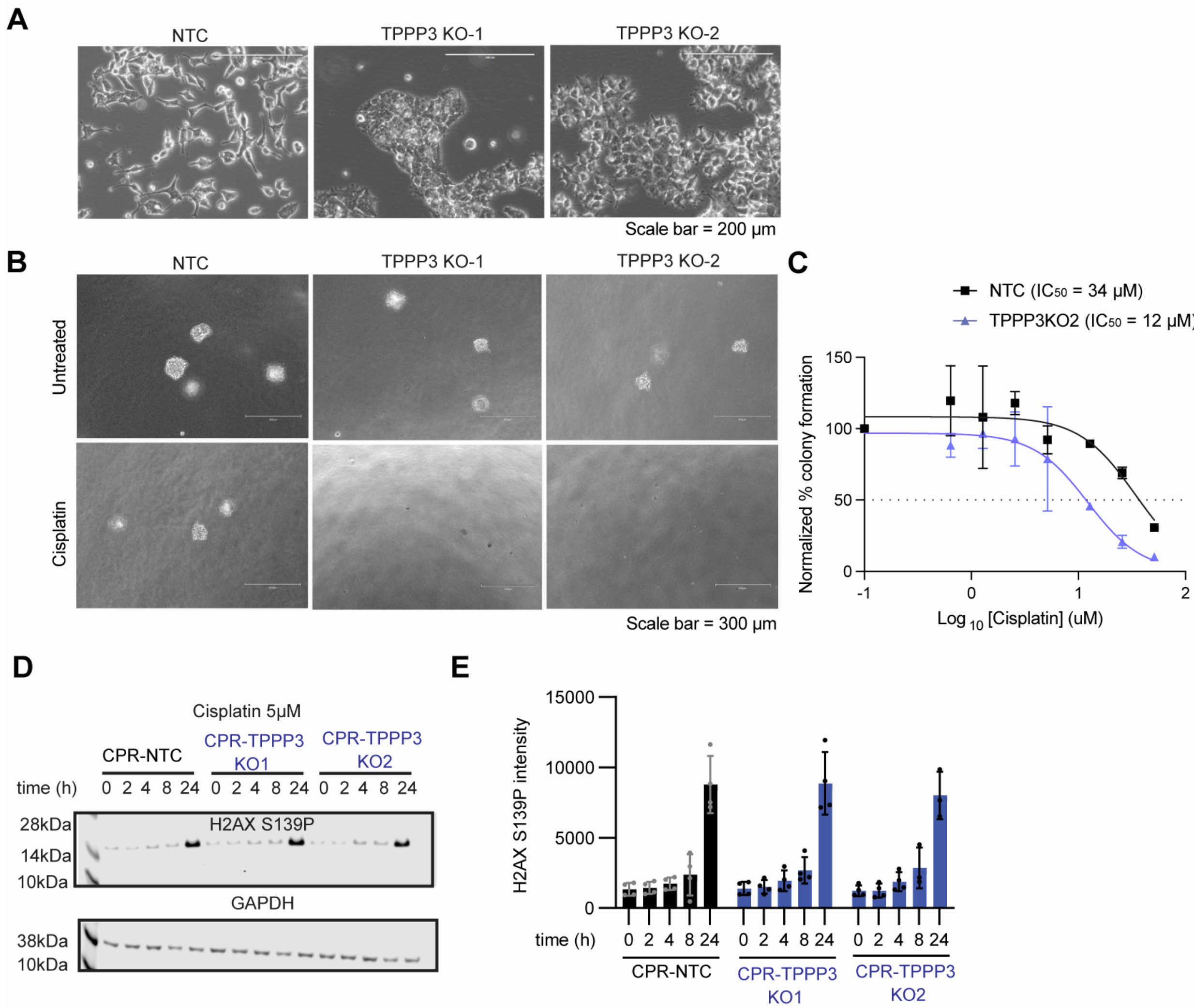

Figure S11. Generation of TPPP3-knock-out 1A9CP80 cell lines and response to cisplatin. (related to Figure 6)

(A) Representative wide-field images of non-targeting control (NTC) and TPPP3-KO cells. Scale bar = 200 µm.

(B) Representative wide-field images of NTC and TPPP3-KO cells grown in soft agar in the presence or absence of 25.6 µM cisplatin, a dose where >50% of NTC cells and <50% of TPPP3-KO cells survive based on the cisplatin dose assay. Scale bar = 300 µm.

(C) % Colony formation of NTC and TPPP3KO2 using the soft agar assay. n = 3 independent experiments.

(D-E) Western blot images (G) and quantification of H2AX S139P (H) at different timepoints after treatment with cisplatin in cisplatin resistant non-targeting control and cisplatin resistant TPPP3 knockout cells; n = 4 independent experiments on different days.

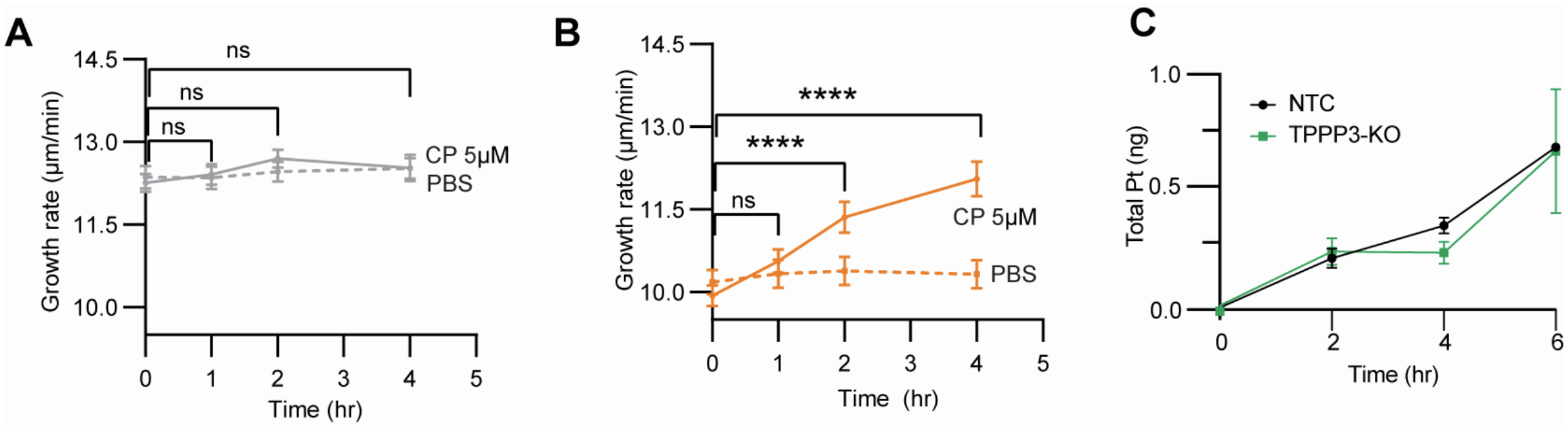

Figure S12. Effects of TPPP3 and cisplatin on microtubule dynamics in ovarian cancer cells. (related to Figure 6)

(A-B) Microtubule growth rates in non-targeting control (NTC) cisplatin-resistant cells (A) and cisplatin re-sensitized cells (B) measured at different time points after the addition of 5 μM cisplatin (thick lines) or PBS control (dotted lines) by tracking EB3-mScarlet comets; n = 40, 40, 38, and 31 cisplatin treated non-targeting control cells at 0, 1, 2, 4 hrs, respectively (90308, 73030, 64282, and 50605 EB3 comets analyzed); n = 40, 40, 40 and 40 PBS treated non-targeting control cells at 0, 1, 2, 4 hrs, respectively (96238, 76723, 72421, and 66657 EB3 comets analyzed); n = 36, 35, 31, and 16 cisplatin treated cisplatin re-sensitized cells at 0, 1, 2, 4 hrs, respectively (52651, 43362, 28730, and 15418 EB3 comets analyzed); n = 25, 25, 23, and 22 PBS treated non-targeting control cells at 0, 1, 2, 4 hrs, respectively (40237, 38946, 31057, and 22679 EB3 comets analyzed) from at least 2 independent experiments on different days. Error bars, SD. **** denotes $p < 0.0001$ and ns denotes non-significant, as determined by Mann-Whitney test.

(C) Inductively coupled plasma mass spectrometry shows total platinum associated with DNA in non-targeting control cisplatin-resistant and TPPP3 KO cisplatin-resistant cells upon treatment with 80 μM cisplatin at time 0, 2, 3, and 6 hours; n = 2 independent experiments.

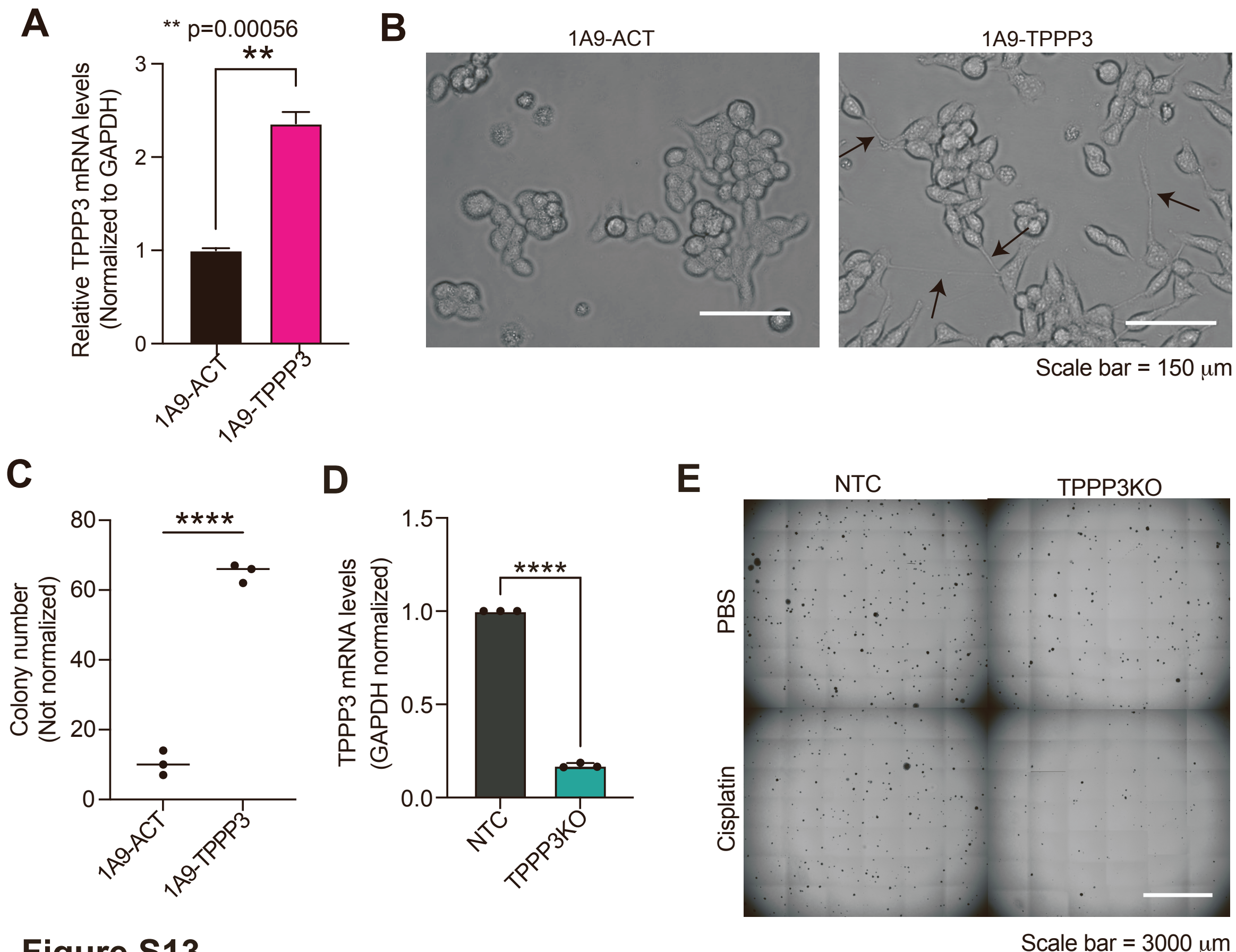

Figure S13

Figure S13. TPPP3-activation in cisplatin-sensitive cells induces resistance to cisplatin while TPPP3-knock-out in cisplatin-resistant OVCAR8-CP5 cells induces sensitivity to cisplatin. (related to Figure 6)

(A) qPCR analyses of TPPP3 in sensitive cells:1A9-ACT (activation control) and 1A9-TPPP3 activated cells. GAPDH was used as a reference control. n = 2 independent experiments. **, $p < 0.01$.

(B) Representative wide-field images of 1A9-ACT and 1A9-TPPP3 cells. Arrows indicate cell protrusions found in TPPP3-activated cells. Scale bar = 150 μm.

(C) TPPP3 promotes cell proliferation. Soft-agar cytotoxicity assay of 1A9-activation (1A(-ACT) and 1A9-TPPP3 activated (1A9-TPPP3) cells reveals increased cell proliferation when TPPP3 is overexpressed; **** $p < 0.0001$ as determined by a student's t-test.

(D) qPCR of non-targeting CRISPR resistant cells (NTC) and CRISPR-knock out of TPPP3 (TPPP3-KO) in OVCAR8-CP5 resistant cells. qPCR was performed in 3 independent experiments and normalized to GAPDH. **** $p <0.0001$.

(E) Representative wide-field images of NTC and TPPP3-KO OVCAR8-CP5 cells grown in soft agar in the presence or absence of 2.56 μM cisplatin. Scale bar = 3000 μm.
